# Supplementary material for: Histopathologic patterns and factors associated with cervical lesions at Jimma Medical Center, Jimma, Southwest Ethiopia: A two-year cross-sectional study
Source: PLoS One. 2024 Apr 18;19(4):e0301559. doi: 10.1371/journal.pone.0301559 (PMC11034979; doi:10.1371/journal.pone.0301559)
Supplement: S1 Checklist — (PDF) [file pone.0301559.s001.pdf]

Annex 2: - Checklist for Data collection

Please fill in the form below from the Biopsy Reports Containing Cervical tissue specimens and/or Diagnosis

1. Year of Biopsy

A. 2018---------------------------------

B. 2019---------------------------------

2, Age Of the patients

A. <30---------------------

B. 31-40-------------------

C. 41-50-------------------

D. 51-60-------------------

E. 61-70-------------------

F. >70----------------------

3. Place of residence/Address of patients

A. Jimma Town---

B. Woredas (Eg Limmu, Seka, Chora) ----------------------

C.SNNPR (Eg Mizan, Bonga, Tercha) ----------------------

4. Nature of specimen/type of biopsies

A. Punch Biopsies--

B. Hysterectomy specimen-------------------------------------

C.Cone Biopsies ---

D.Others---

5. Clinical features/presentation

A.Abnormal vaginal bleeding----------------------------------

B. Vaginal discharge--

C.Dyspareunia/pain during intercourse -----------------------

D.Others---

6. Duration of symptoms

A. <1month -------------------------------------

B. 1-3months-------------------------------------

C.3-6months -------------------------------------

D.6months-2years-------------------------------

E.>2years---

7. Type of Cervical Lesions

A. Precancerous cervical lesion (LSIL and HSIL) -------------------------------------

B. Cancerous cervical lesion (Cervical cancer) -----------------------------

C. Non-cancerous cervical lesion (Eg, Cervicitis, Polyp) ---------------------

D. Others--

8. Non-cancerous cervical lesion

A. Polyp--------------------------------------

B. Cervicitis----------------------------------

C. Others-------------------------------------

9. Precancerous and cancerous cervical lesion

A.LSIL--

B.HSIL --

D.carcinoma--------------------------

E.Others---

10. Histopathological classification of cervical cancer

A. Squamous cell carcinoma -----------------------------------

B. Adenocarcinoma ---

C Adenosquamous carcinoma -----------------------------------

D. Carcinoma, unspecified -------------------------------------

E. Others---

11. Histologic type of SCC

A. Keratinazing------------------------------------

B. Nonkeratinizing-----------------------------

C. Others--
