# Supplementary material for: Histopathologic patterns and factors associated with cervical lesions at Jimma Medical Center, Jimma, Southwest Ethiopia: A two-year cross-sectional study
Source: PLoS One. 2024 Apr 18;19(4):e0301559. doi: 10.1371/journal.pone.0301559 (PMC11034979; doi:10.1371/journal.pone.0301559)
Supplement: S1 Checklist — (PDF) [file pone.0301559.s002.pdf]

STROBE Statement—checklist of items that should be included in reports of observational studies

|  | Item No. | Recommendation | Page  No. | Relevant text from manuscript |
| --- | --- | --- | --- | --- |
| **Title and abstract** | 1 | (*a*) Indicate the study’s design with a commonly used term in the title or the abstract | Title page | Cross-sectional |
|  |  | (*b*) Provide in the abstract an informative and balanced summary of what was done and what was found | I | cervical cancer,histopathology, cervical lesions, |
| Introduction | | | |  |
| Background/rationale | 2 | Explain the scientific background and rationale for the investigation being reported | 1,4 | In Ethiopia, cervical cancer is the second most common female cancer. PAP smears are only performed in a few centers across the country |
| Objectives | 3 | State-specific objectives, including any prespecified hypotheses | 5 | to determine the histopathologic patterns of cervical lesions |
| Methods | | | |  |
| Study design | 4 | Present key elements of study design early in the paper | 6 | A 2-year facility-based cross-sectional study was conducted from May 1 to June 30, 2019. |
| Setting | 5 | Describe the setting, locations, and relevant dates, including periods of recruitment, exposure, follow-up, and data collection | 6 | The study was conducted at Jimma Medical Center, formerly called Jimma University Specialized Hospital (JUSH). |
| Participants | 6 | (*a*) *Cohort study*—Give the eligibility criteria, and the sources and methods of selection of participants. Describe methods of follow-up  *Case-control study*—Give the eligibility criteria, and the sources and methods of case ascertainment and control selection. Give the rationale for the choice of cases and controls  *Cross-sectional study*—Give the eligibility criteria, and the sources and methods of selection of participants | 7 | All female patients who submitted cervical tissue specimens to the pathology department from September 12, 2018, to September 12, 2020. |
|  |  | (*b*) *Cohort study*—For matched studies, give matching criteria and number of exposed and unexposed  *Case-control study*—For matched studies, give matching criteria and the number of controls per case |  |  |
| Variables | 7 | Clearly define all outcomes, exposures, predictors, potential confounders, and effect modifiers. Give diagnostic criteria, if applicable | 8 | ***Dependent variable***   - Histopathologic pattern of Cervical tissue specimens |
| Data sources/ measurement | 8* | For each variable of interest, give sources of data and details of methods of assessment (measurement). Describe the comparability of assessment methods if there is more than one group | 8 | Histopathology reports of biopsies submitted from cervical lesions that were routinely processed |
| Bias | 9 | Describe any efforts to address potential sources of bias | 8 | the checklist was pretested on 47 cases (10% of the total sample size) of hard-copy biopsy reports done in the year 2017 that were not included in the current study. |
| Study size | 10 | Explain how the study size was arrived at | 7 | Diagram showing the sampling procedure of the selected 469 cervical lesion biopsy records from 2018 to 2020 |

Continued on next page

| Quantitative variables | 11 | Explain how quantitative variables were handled in the analyses. If applicable, describe which groupings were chosen and why | 8 | Eligible 469 reports fulfilling inclusion and exclusion criteria were extracted and recorded into a prepared checklist containing study variables. |
| --- | --- | --- | --- | --- |
| Statistical methods | 12 | (*a*) Describe all statistical methods, including those used to control for confounding | 8 | Data were cleaned, coded, entered into EpiData v3.1, and exported to SPSS version 26 for analysis. |
|  |  | (*b*) Describe any methods used to examine subgroups and interactions | 8 | Cross tabulation, the chi-square test, and logistic regression with multivariate analysis were performed |
|  |  | (*c*) Explain how missing data were addressed | 7 | ***Exclusion Criteria***  Biopsy reports missing two or more independent variables |
|  |  | (*d*) *Cohort study*—If applicable, explain how the loss to follow-up was addressed  *Case-control study*—If applicable, explain how the matching of cases and controls was addressed  *Cross-sectional study*—If applicable, describe analytical methods taking account of the sampling strategy |  | Not Applicable |
|  |  | (*e*) Describe any sensitivity analyses |  | Not applicable |
| Results | | | | |
| Participants | 13* | (a) Report numbers of individuals at each stage of study—eg numbers potentially eligible, examined for eligibility, confirmed eligible, included in the study, completing follow-up, and analysed | 10 | From 2613 female biopsy reports, 543 biopsy reports of cervical lesion specimens were extracted |
|  |  | (b) Give reasons for non-participation at each stage | 10 | 74 were excluded because they fulfilled the exclusion criteria. |
|  |  | (c) Consider use of a flow diagram | 7 | Diagram showing the sampling procedure of the selected 469 cervical lesion biopsy records from 2018 to 2020 |
| Descriptive data | 14* | (a) Give characteristics of study participants (eg demographic, clinical, social) and information on exposures and potential confounders | 11,12 | Bar graph showing the distribution of cervical lesions among residents of Jimma town and surrounding areas |
|  |  | (b) Indicate number of participants with missing data for each variable of interest |  | Biopsy reports included in the study were complete. |
|  |  | (c) *Cohort study*—Summarise follow-up time (eg, average and total amount) |  | Not applicable |
| Outcome data | 15* | *Cohort study*—Report numbers of outcome events or summary measures over time |  | Not applicable |
|  |  | *Case-control study—*Report numbers in each exposure category, or summary measures of exposure |  | Not applicable |
|  |  | *Cross-sectional study—*Report numbers of outcome events or summary measures | 14 | Most (87%) of the specimens were precancerous and cancerous cervical lesions, while 13% were benign cervical lesions |
| Main results | 16 | (*a*) Give unadjusted estimates and, if applicable, confounder-adjusted estimates and their precision (eg, 95% confidence interval). Make clear which confounders were adjusted for and why they were included | 17 | Age (*p*=**0.001, COR=1.048**) and place of residence (*p*= **0.032, COR=0.531**) were tested at a P value less than 0.05 in bivariate logistic regression |
|  |  | (*b*) Report category boundaries when continuous variables were categorized | 11 | Figure 3: Bar graph showing cervical lesion status with age groups |
|  |  | (*c*) If relevant, consider translating estimates of relative risk into absolute risk for a meaningful time period |  | Not applicable |

Continued on next page

| Other analyses | 17 | Report other analyses done—eg analyses of subgroups and interactions, and sensitivity analyses | 17 | Multivariate logistic regression was performed on these variables |
| --- | --- | --- | --- | --- |
| Discussion | | | | |
| Key results | 18 | Summarise key results with reference to study objectives | 18 | Of the 469 biopsies submitted to JMC, the department of pathology, most (87%) of the specimens were precancerous and cancerous cervical lesions, while 13% were benign cervical lesions. |
| Limitations | 19 | Discuss limitations of the study, taking into account sources of potential bias or imprecision. Discuss both direction and magnitude of any potential bias | 9 | Confirmatory immunohistochemistry and HPV DNA testing could not be included in this study because of the limited setup. |
| Interpretation | 20 | Give a cautious overall interpretation of results considering objectives, limitations, multiplicity of analyses, results from similar studies, and other relevant evidence | 18 | Another study done at TASH, A.A., Ethiopia on women with histologically verified cancer of the cervix uteri also showed that the mean age was 49 years (21–91 years) (21). Our findings are consistent with this and other similar studies. |
| Generalisability | 21 | Discuss the generalisability (external validity) of the study results | 4 | The study will serve as a baseline for future studies to be carried out at the national or continental level during the post-HPV vaccination era. |
| Other information | |  | | |
| Funding | 22 | Give the source of funding and the role of the funders for the present study and, if applicable, for the original study on which the present article is based |  | There are no funders that offered grants for the research. Jimma and Mizan-Tepi Universities covered the cost of stationeries and data collectors. |

*Give information separately for cases and controls in case-control studies and, if applicable, for exposed and unexposed groups in cohort and cross-sectional studies.

**Note:** An Explanation and Elaboration article discusses each checklist item and gives methodological background and published examples of transparent reporting. The STROBE checklist is best used in conjunction with this article (freely available on the Web sites of PLoS Medicine at http://www.plosmedicine.org/, Annals of Internal Medicine at http://www.annals.org/, and Epidemiology at http://www.epidem.com/). Information on the STROBE Initiative is available at www.strobe-statement.org.
