# Supplementary material for: Histopathologic patterns and factors associated with cervical lesions at Jimma Medical Center, Jimma, Southwest Ethiopia: A two-year cross-sectional study
Source: PLoS One. 2024 Apr 18;19(4):e0301559. doi: 10.1371/journal.pone.0301559 (PMC11034979; doi:10.1371/journal.pone.0301559)
Supplement: S2 File — (DOCX) [file pone.0301559.s004.docx]

Abbreviations and Acronyms

| AOR | Adjusted Odds Ratio |  |
| --- | --- | --- |
| BMA | Bone marrow Aspiration |  |
| CA | Cancer/carcinoma |  |
| CIN | Cervical Intraepithelial Lesions |  |
| COR  EFMOH | Crude Odds Ratio  Ethiopian Federal Ministry of Health |  |
| FIGO | International Federation of Gynecology and Obstetrics |  |
| FIG | Figure |  |
| FNAC | Fine Needle Aspiration Cytology |  |
| GLOBOCAN | Global Cancer Incidence, Mortality, and Prevalence |  |
| H & E | Hematoxylin and Eosin |  |
| HIV | Human immunodeficiency virus |  |
| HSIL | High-grade squamous intraepithelial lesion |  |
| HPV | Human Papilloma Virus |  |
| IARC | International Agency for Research on Cancer |  |
| IRB | Institutional Review Board |  |
| JMC | Jimma Medical Center |  |
| JUSH | Jimma University Specialized Hospital |  |
| LEEP | Loop Electrical Excision Procedure |  |
| LSIL | low-grade squamous intraepithelial lesion |  |
| NCCP | National Cancer Control Plan of Ethiopia |  |
| PAP | Papanicolaou stain |  |
| PMB | Postmenopausal bleeding |  |
| SCC | Squamous Cell Carcinoma |  |
| SPHMMC | Saint Paul Hospital Millennium Medical College |  |
| SPSS  STD | Statistical Package for the Social Science  Sexually transmitted diseases |  |
| STI | Sexually transmitted infection |  |
| TASH  UOG | Tikur Anbessa Specialized Hospital  University of Gonder |  |
| VIA | Visual inspection with acetic acid |  |
| WHO | World Health Organization |  |
