# Supplementary material for: Histopathologic patterns and factors associated with cervical lesions at Jimma Medical Center, Jimma, Southwest Ethiopia: A two-year cross-sectional study
Source: PLoS One. 2024 Apr 18;19(4):e0301559. doi: 10.1371/journal.pone.0301559 (PMC11034979; doi:10.1371/journal.pone.0301559)
Supplement: Attachment — Submitted filename: PLOS one, commented paper.pdf [file pone.0301559.s006.pdf]

PLOS ONE

Histopathologic patterns and factors associated with cervical lesions at Jimma Medical Center, Jimma, Southwest Ethiopia: A two-year crosssectional study

--Manuscript Draft--

**Manuscript Number:**	PONE-D-23-17869R1
**Article Type:**	Research Article
**Full Title:**	Histopathologic patterns and factors associated with cervical lesions at Jimma Medical Center, Jimma, Southwest Ethiopia: A two-year crosssectional study
**Short Title:**	Histopathologic patterns and factors associated with cervical lesions at Jimma Medical Center
**Corresponding Author:**	Birhanu Hailu Tirkaso, M.D.<br>Mizan-Tepi University<br>Mizan, Southwestern Region ETHIOPIA
**Keywords:**	Histopathology,<br>Cervical Lesions,<br>Cervical Cancer,<br>Jimma Medical Center,<br>Ethiopia.
**Abstract:**	BackgroundThe cervix is the lower portion of the uterus, which connects this organ to the vagina through the endocervical canal.ObjectiveThis study aimed to determine the histopathologic patterns and factors associated with cervical lesions at Jimma Medical Center from September 12, 2018, to September 12, 2020.MethodsA 2-year facility-based cross-sectional study was conducted from May 1 to June 30, 2019.ResultIn this study, cervical cancer was the most common (71%) cause of cervical lesions. Squamous cell carcinoma was the most frequent cervical cancer diagnosed during the study, accounting for 96.4% of 331 cancerous cases, followed by adenocarcinoma (3.3%). High-grade squamous intraepithelial lesions were the most frequently diagnosed precancerous lesions, accounting for 68.4% of cases. Endocervical polyps were the most commonly diagnosed benign lesions, accounting for 59.3% of cases.ConclusionThe maximum age distribution of cervical lesions was in the 41–50-year age range. Squamous cell carcinoma was the most frequent type of cervical cancer. High-grade squamous intraepithelial lesions were the most frequently diagnosed precancerous cervical lesions. The most common benign cervical lesions were endocervical polyps.RecommendationWe recommend educating the community to improve health-seeking behavior and on possible preventive strategies for cervical cancer.
**Order of Authors:**	Birhanu Hailu Tirkaso, M.D.,Assistant Professor of Pathology
	Tesfaye Hurgesa Bayisa, MD,Assistant Professor of Pathology
	Tewodros Wubshet Desta, MD,Assistant Professor of Pathology
**Response to Reviewers:**	Response to Academic Editor:-Thank you very much for your insightful comments and I have tried to address the comments as requested.<br>1.The manuscript is now revised according to PLOS ONE's style requirements in accordance with the formatting guidelines provided. We have updated the title page and renamed the chapters as outlined in the PLOS ONE guidelines. Figures were also formatted according to the guidelines and included in the main document. The figures will also be uploaded as a separate file in case they are needed during manuscript processing.<br>2.Regarding the minor occurrence of overlapping text with previous publications, we revised the specific paragraphs and rephrased the text to avoid plagiarism and we have cited the paper mentioned by the academic editor. Please see pages 1-3. The revised manuscript is checked for plagiarism using online checkers and checked for similarity index using Turnitin before resubmission. The majority of the similarity index came from the same thesis of the corresponding author put on the institutional website by Jimma University to avoid topic redundancy for future graduates but was not formally published. One thing, we want to clarify is that this manuscript is the refined

Powered by Editorial Manager® and ProduXion Manager® from Aries Systems Corporation

	version of the manuscript posted on Jimma University's website: https://repository.ju.edu.et/handle/123456789/4080.<br>3.Concerning dual publications, the manuscript was not peer-reviewed and formally published anywhere. As a part of second-degree completion, Jimma University put the thesis of postgraduate graduates on its website so that each graduate student selects a unique research topic.We put the link to the institutional repository to indicate that our work is based on the material posted on the website. Because of this, this work does not constitute a dual publication.<br>4.The other issue raised was the data availability statement. All relevant data are within the manuscript and its Supporting Information files. Please update this for us.<br>Response to Reviewer #1:-Thank you so much for your nice comments,Pie charts have been replaced by bar graphs.<br>Response to Reviewer #2: Thank you for your in-depth revision.The discussion part has been expanded now to include the type of biopsies (Nature of specimen) by incorporating additional references. Because the focus of this thesis is to show the magnitude of the cervical cancer from Pathology point of view,it will be difficult for us to go beyond this to the details of surgical management options due to the limited set up.I am eternally grateful.
Additional Information:	
**Question**	**Response**
**Financial Disclosure**<br><br>Enter a financial disclosure statement that describes the sources of funding for the work included in this submission. Review the submission guidelines for detailed requirements. View published research articles from *PLOS ONE* for specific examples.<br><br>This statement is required for submission and **will appear in the published article** if the submission is accepted. Please make sure it is accurate.<br><br>**Unfunded studies**<br>Enter: *The author(s) received no specific funding for this work.*<br><br>**Funded studies**<br>Enter a statement with the following details:<br>• Initials of the authors who received each award<br>• Grant numbers awarded to each author<br>• The full name of each funder<br>• URL of each funder website<br>• Did the sponsors or funders play any role in the study design, data collection and analysis, decision to publish, or preparation of the manuscript?<br>• **NO** - Include this sentence at the end of your statement: *The funders had no role in study design, data collection and analysis, decision to publish, or preparation of the manuscript.*<br>• **YES** - Specify the role(s) played.	The author(s) received no specific funding for this work.

* typeset	
**Competing Interests**<br><br>Use the instructions below to enter a competing interest statement for this submission. On behalf of all authors, disclose any competing interests that could be perceived to bias this work—acknowledging all financial support and any other relevant financial or non-financial competing interests.<br><br>This statement is required for submission and **will appear in the published article** if the submission is accepted. Please make sure it is accurate and that any funding sources listed in your Funding Information later in the submission form are also declared in your Financial Disclosure statement.<br><br>View published research articles from *PLOS ONE* for specific examples.<br><br>**NO authors have competing interests**<br><br>Enter: *The authors have declared that no competing interests exist.*<br><br>**Authors with competing interests**<br><br>Enter competing interest details beginning with this statement:<br><br>*I have read the journal's policy and the authors of this manuscript have the following competing interests: [insert competing interests here]*<br><br>* typeset	The authors have declared that no competing interests exist.
**Ethics Statement**<br><br>Enter an ethics statement for this submission. This statement is required if the study involved:	Before data collection, the proposal for this study was submitted to Jimma University's research and ethical committee. Then, ethical clearance for the use of secondary data and a waiver of informed consent was obtained from the Institutional Review Board (IRB) of Jimma Medical center(JMC) and submitted to the responsible authorities of JMC. A formal letter of cooperation was also written to obtain permission from the head

• Human participants<br>• Human specimens or tissue<br>• Vertebrate animals or cephalopods<br>• Vertebrate embryos or tissues<br>• Field research<br><br>Write "N/A" if the submission does not require an ethics statement.<br><br>General guidance is provided below. Consult the submission guidelines for detailed instructions. **Make sure that all information entered here is included in the Methods section of the manuscript.**	of the pathology department before using the data. All the information collected from the study was handled confidentially by omitting the personal identification of the pathology reports, and data utilization was carried out in accordance with the ethical and scientific standards outlined in national and international guidelines.The Ethical clearance document is attached along with other supporting documents.

Powered by Editorial Manager® and ProduXion Manager® from Aries Systems Corporation

**Format for specific study types**<br><br>**Human Subject Research (involving human participants and/or tissue)**<br>• Give the name of the institutional review board or ethics committee that approved the study<br>• Include the approval number and/or a statement indicating approval of this research<br>• Indicate the form of consent obtained (written/oral) or the reason that consent was not obtained (e.g. the data were analyzed anonymously)<br><br>**Animal Research (involving vertebrate animals, embryos or tissues)**<br>• Provide the name of the Institutional Animal Care and Use Committee (IACUC) or other relevant ethics board that reviewed the study protocol, and indicate whether they approved this research or granted a formal waiver of ethical approval<br>• Include an approval number if one was obtained<br>• If the study involved *non-human primates*, add *additional details* about animal welfare and steps taken to ameliorate suffering<br>• If anesthesia, euthanasia, or any kind of animal sacrifice is part of the study, include briefly which substances and/or methods were applied<br><br>**Field Research**<br><br>Include the following details if this study involves the collection of plant, animal, or other materials from a natural setting:<br>• Field permit number<br>• Name of the institution or relevant body that granted permission	
**Data Availability**<br><br>Authors are required to make all data underlying the findings described fully available, without restriction, and from the time of publication. PLOS allows rare exceptions to address legal and ethical concerns. See the PLOS Data Policy and FAQ for detailed information.	Yes - all data are fully available without restriction

A Data Availability Statement describing where the data can be found is required at submission. Your answers to this question constitute the Data Availability Statement and **will be published in the article**, if accepted.<br><br>**Important:** Stating 'data available on request from the author' is not sufficient. If your data are only available upon request, select 'No' for the first question and explain your exceptional situation in the text box.<br><br>Do the authors confirm that all data underlying the findings described in their manuscript are fully available without restriction?	
**Describe where the data may be found in full sentences. If you are copying our sample text, replace any instances of XXX with the appropriate details.**<br><br>• If the data are **held or will be held in a public repository**, include URLs, accession numbers or DOIs. If this information will only be available after acceptance, indicate this by ticking the box below. For example: *All XXX files are available from the XXX database (accession number(s) XXX, XXX.).*<br>• If the data are all contained **within the manuscript and/or Supporting Information files**, enter the following: *All relevant data are within the manuscript and its Supporting Information files.*<br>• If neither of these applies but you are able to provide **details of access elsewhere**, with or without limitations, please do so. For example:<br><br>*Data cannot be shared publicly because of [XXX]. Data are available from the XXX Institutional Data Access / Ethics Committee (contact via XXX) for researchers who meet the criteria for access to confidential data.*<br><br>*The data underlying the results presented in the study are available from (include the name of the third party*	All relevant data are within the manuscript and its Supporting Information files.

and contact information or URL).<br>• This text is appropriate if the data are owned by a third party and authors do not have permission to share the data.<br><br>* typeset	
Additional data availability information:	Tick here if your circumstances are not covered by the questions above and you need the journal's help to make your data available.

Powered by Editorial Manager® and ProduXion Manager® from Aries Systems Corporation

Manuscript

Click here to access/download;Manuscript;Cervical lesions main document.docx

Histopathologic patterns and factors associated with cervical lesions at Jimma Medical Center, Jimma, Southwest Ethiopia: A two-year cross-sectional study

Corresponding Author: -

***Dr. Birhanu Hailu Tirkaso (MD, Pathologist)**

Assistant Professor of Pathology

Department of Biomedical Sciences, Mizan-Tepi University

Mizan, Southwestern Region, Ethiopia

P.o.box. 260

(+251) 47-336-0258

Email: - birhanuhailu973@gmail.com

birhanuhailu@mtu.edu.et

Coauthors: -

Dr. Tesfaye Hurgesa Bayisa (MD, Pathologist)

Assistant Professor of Pathology

Department of Pathology, Jimma University

Jimma, Oromia, Ethiopia

P.o.box 378

(+251) 471 11 0102

Email: -thurgesa123@gmail.com

Dr. Tewodros Wubshet Desta (MD, Pathologist)

Assistant Professor of Pathology

Department of Pathology, Dire Dawa University

Dire Dawa, Ethiopia

P.o.box 1362

Fax: - (+251) 251127971

Email: - theddo04@gmail.com

Abstract

Background: The cervix is the lower portion of the uterus, which connects this organ to the vagina through the endocervical canal.

Objective: This study aimed to determine the histopathologic patterns and factors associated with cervical lesions at Jimma Medical Center from September 12, 2018, to September 12, 2020.

Methods: A 2-year facility-based cross-sectional study was conducted from May 1 to June 30, 2019.

Result: In this study, cervical cancer was the most common (71%) cause of cervical lesions. Squamous cell carcinoma was the most frequent cervical cancer diagnosed during the study, accounting for 96.4% of 331 cancerous cases, followed by adenocarcinoma (3.3%). High-grade squamous intraepithelial lesions were the most frequently diagnosed precancerous lesions, accounting for 68.4% of cases. Endocervical polyps were the most commonly diagnosed benign lesions, accounting for 59.3% of cases.

Conclusion: The maximum age distribution of cervical lesions was in the 41–50-year age range. Squamous cell carcinoma was the most frequent type of cervical cancer. High-grade squamous intraepithelial lesions were the most frequently diagnosed precancerous cervical lesions. The most common benign cervical lesions were endocervical polyps.

Recommendation: We recommend educating the community to improve health-seeking behavior and on possible preventive strategies for cervical cancer.

Keywords: Histopathology, Cervical Lesions, Cervical Cancer, Jimma Medical Center, Ethiopia.

Abbreviations and Acronyms

AOR	Adjusted Odds Ratio
BMA	Bone marrow Aspiration
CA	Cancer/carcinoma
CIN	Cervical Intraepithelial Lesions
COR	Crude Odds Ratio
EFMOH	Ethiopian Federal Ministry of Health
FIGO	International Federation of Gynecology and Obstetrics
FIG	Figure
FNAC	Fine Needle Aspiration Cytology
GLOBOCAN	Global Cancer Incidence, Mortality, and Prevalence
H & E	Hematoxylin and Eosin
HIV	Human immunodeficiency virus
HSIL	High-grade squamous intraepithelial lesion
HPV	Human Papilloma Virus
IARC	International Agency for Research on Cancer
IRB	Institutional Review Board
JMC	Jimma Medical Center
JUSH	Jimma University Specialized Hospital
LEEP	Loop Electrical Excision Procedure
LSIL	low-grade squamous intraepithelial lesion
NCCP	National cancer control plan of Ethiopia
PAP	Papanicolaou stain
PMB	Postmenopausal bleeding
SCC	Squamous Cell Carcinoma
SPHMMC	Saint Paul Hospital Millennium Medical College
SPSS	Statistical Package for the Social Science
STD	Sexually transmitted diseases
STI	Sexually transmitted infection
TASH	Tikur Anbessa Specialized Hospital
UOG	University of Gonder
VIA	Visual inspection with acetic acid
WHO	World Health Organization

1. Introduction

1.1. Background

The cervix is the lower portion of the uterus, which connects this organ to the vagina through the endocervical canal. Most of the exocervix is covered by non-keratinizing squamous epithelium. The glandular mucosa of the endocervix is formed by a layer of columnar mucus-secreting cells; the area where the squamous and glandular epithelia meet is known as the squamocolumnar junction(1). The squamocolumnar junction undergoes eversion with the onset of puberty, causing columnar epithelium to become visible on the exocervix. The exposed columnar cells eventually undergo squamous metaplasia, forming a region called the transformation zone(2). The transformation zone is the area where the expected transformation to the metaplastic squamous epithelium and the abnormal transformation to CIN usually occur(3).

The range of neoplastic conditions of the cervix arises at or near the cervical transformation zone, ranging from precancerous intraepithelial neoplasia to epithelial malignancies, mixed epithelial and mesenchymal tumors, mesenchymal tumors, melanocytic tumors, and miscellaneous rare malignancies(4).

The epidemiology of precancerous lesions and squamous cell carcinoma (SCC) of the cervix follows the epidemiology of HPV infections. Adenocarcinoma currently comprises 10–25% of all cervical carcinomas in developed countries, compared to 5–10% three decades ago. This increase has resulted from a decline in squamous cell carcinomas secondary to screening programs and better identification of glandular lesions in cervical cytology samples. A two-tier system of low- and high-grade intraepithelial lesions is more biologically relevant and histologically more reproducible than the three-tier CIN 1, CIN 2, and CIN 3 terminology used in the prior edition and is therefore recommended. Our improved understanding of HPV biology supports the grouping of CIN 2 with CIN 3 under the HSIL designation. SCC of the usual type may be nonkeratinizing or keratinizing (5).

According to a 5-year retrospective study done on 75 cases in Nigeria, cervical cancer has an incidence of 65.2% of all gynecological cancers and 13.4% of all gynecological admissions. The modal age was in the 60-70 years range. SCC of varying differentiation was the most common histological type, accounting for 89.3 %, and adenocarcinoma accounted for only 8.0%. The modes of presentation were post-menopausal bleeding (84.0%), vaginal discharge (72.0%), contact bleeding (63.9%), and abdominal pain (56.2%)(6).

1.2. Problem statement

Worldwide, the overall burden of cancer is estimated to continue to rise, particularly in developing countries. It is estimated that twenty-one million people will be diagnosed and thirteen million will die of cancer by the year 2030. Although incidence rates for all cancers combined are twice as high in more developed compared to less developed countries, mortality rates are only 8% to 15% higher in more-developed countries. This disparity primarily reflects differences in the availability of and accessibility to diagnostics and treatment. Similarly, cancers are more often detected at a later stage in less-developed countries, which contributes to the disparity in mortality rates relative to the incidence. Although the national cancer control plan of Ethiopia/NCCP/ set ambitious objectives to expand the methods of preventive intervention, launching screening tests for early detection, and diagnosis and treatment with the provision of chemotherapy, surgery and palliative care, the effects on cervical cancer take years to show significant decline as observed in the developed nations (7).

Cervical cancer is one of the most common cancers in women, with approximately 0.5 million cases worldwide in 2008. Cervical cancers worldwide increased from an estimated 378,000 in 1980 to 500,000 per year in recent years, reflecting an average annual increase of 0.6%. Approximately 76% of recent cases occur in low-resource nations, with numbers increasing in all but high-income countries. The median age at death in wealthier nations is 55 years. The large disparities in incidence and mortality between heavily affected nations in Africa and Asia and lower rates in wealthier North American and European countries are largely attributable to effective programs to detect cancer precursors coupled with infrastructure to clinically manage precursor lesions (5) (8).

Cancer is one of the major non-communicable diseases (NCDs). Every year, NCDs and cancer cause over 60% of total global mortality. The incidence and mortality from NCDs are rising rapidly, while communicable diseases still remain the leading killers in many developing countries. This has resulted in a double disease burden, which is putting strain on the existing health system (7) (9).

According to GLOBOCAN 2018, the global cancer burden has risen to 18.1 million cases and 9.6 million cancer deaths. One in six women and one in five men worldwide develop cancer during their lifetime, and one in eleven women and one in eight men die from the disease. In the future, the incidence of cancer worldwide is expected to rise to 26.4 million, with 17 million deaths by 2030 (10,11).

A 2-year retrospective study done on 3231 samples (1263 biopsies and 1968 FNAC) at UOG: North-West Ethiopia showed that 540 (16.7%) were malignant cancer cases. Lymphomas, cervical cancer, and breast cancer are the three most common cancers in all age groups. Cervical cancer and breast cancer were the two most common cancer types in the hospital, with cervical cancer ranking first (12).

In 2015, an estimated 21,563 and 42,722 incident cancer cases were diagnosed in males and females, respectively, according to a study based on primary data on 8539 patients from Addis Abeba's population-based cancer registry and supplemented by 1,648 cancer case data from six regions. Cervical cancer was the second most common and prevalent cancer, making up 23% of cancer cases, preceded by breast cancer (33%) (13).

Cervical cancer accounts for 22.5% of all cancer cases in women in sub-Saharan Africa, and the majority of women who develop cervical cancer live in rural areas. According to the WHO, cervical cancer will kill >443,000 women per year worldwide by 2030, nearly 90% of them in sub-Saharan Africa (14).

1.3. Rationale of the Study

In Ethiopia, cervical cancer is the second most common female cancer. Throughout the world, HPV infections are the most common cause of cervical cancer. Out of more than 100 different HPV types, infection with HPV 16 and 18 has been associated with more than 70% of cervical cancers. Cervical cancer is a preventable and curable disease. It can be prevented by vaccination and screening and cured if identified at an early stage.

Despite the fact that high-risk HPV infection is a significant cause, only a small percentage of HPV-infected women develop invasive cancer, implying the presence of HPV infection co-factors that lead to cervical cancer development. According to studies carried out in different parts of Ethiopia, including the study area, knowledge about cervical cancer is very low. Although the NCCP launched nationwide HPV vaccinations in the 2016–2019 national cancer control plan, the effects take years to be seen in primary and secondary school students. Furthermore, PAP smears are only performed in a few centers across the country, and other health institutions in the country's outskirts perform a visual inspection with acetic acid (VIA). To my knowledge, no studies have included benign cervical conditions in the study area. The clinicopathologic studies done in the study area were conducted ten years ago.

1.4. Significance of the Study

This study augments the clinicopathologic studies done in southwestern Ethiopia from a histopathologic point of view, including benign cervical conditions. In addition, this study will serve as a baseline for future studies to be carried out at the national or continental level during the post-HPV vaccination era. The result of this study also serves as input for JMC, Zonal and Regional Health Bureaus, and the Ethiopian Federal Ministry of Health (EFMOH) in the evaluation and monitoring of the screening and preventive strategies.

2. Objectives

2.1. General objective

This study aimed to determine the histopathologic patterns and factors associated with cervical lesions at Jimma Medical Center from September 12, 2018, to September 12, 2020.

2.2. Specific objectives

- To describe histopathological patterns of cervical lesions at JMC
- To determine the clinical features of cervical lesions at JMC
- To identify factors associated with cervical lesions at JMC

3 Methods

3.1 Study area

The study was conducted at Jimma Medical Center, formerly called Jimma University Specialized Hospital (JUSH). JMC was established in 1938 in Jimma Town, located 352 kilometers southwest of Addis Ababa, the capital of Ethiopia. Jimma is the main town in southwestern Ethiopia with an estimated population of 120,960. It has a latitude of 7° 41′ 6′′ North, a longitude of 36° 49′ 53′′ East, and an elevation of 1738 meters (5702 feet). Currently, the hospital is a teaching and referral hospital in the southwestern part of the country, providing service for approximately 15,000 inpatients, 160,000 outpatient attendants, 11,000 emergency cases, and 4,500 deliveries in a year, coming from the catchment population of about 15 million (15–17).

The pathology department is one of the most important departments at JMC, providing services such as histopathology diagnostics, FNAC, fluid cytology, and hematopathology, with an annual average flow of 1,928 histopathology biopsy samples, over 5,000 FNAC, and over 200 BMA.

3.2 Study Design and Period

A 2-year facility-based cross-sectional study was conducted from May 1 to June 30, 2019.

3.3 Population

3.3.1 Target population

All female patients in the catchment area of Jimma Medical Center.

3.3.2 Source population

All female patients who submitted biopsy specimens to the pathology department for histopathology diagnosis from September 12, 2018 to September 12, 2020.

3.3.3 Study population

Histopathology reports of all female patients who submitted cervical tissue specimens to the pathology department from September 12, 2018 to September 12, 2020.

3.3.4 Study Unit

Histopathology reports of selected female patients who submitted cervical tissue specimens to the pathology department from September 12, 2018, to September 12, 2020, and fulfilled the inclusion criteria.

3.4 Inclusion and exclusion criteria

3.1.1 Inclusion Criteria

All female patients who submitted cervical tissue specimens to the pathology department from September 12, 2018 to September 12, 2020.

3.1.2 Exclusion Criteria

- Biopsy reports missing two or more independent variables
- Biopsy reports with inconclusive histopathologic diagnosis
- Repeated biopsy

3.5 Sample Size and sampling technique

Histopathology hard copy reports of 543 patients at JMC's pathology department from September 12, 2018, to September 12, 2020, were retrieved from the pathology department's data archive, and those reports that fulfilled the inclusion criteria were manually selected and then grouped by year after being retrieved from the entire hard copy of 3766 histopathology reports in the department's archives. Relevant information was collected via a checklist from 469 hard copies. See figure 1 below.

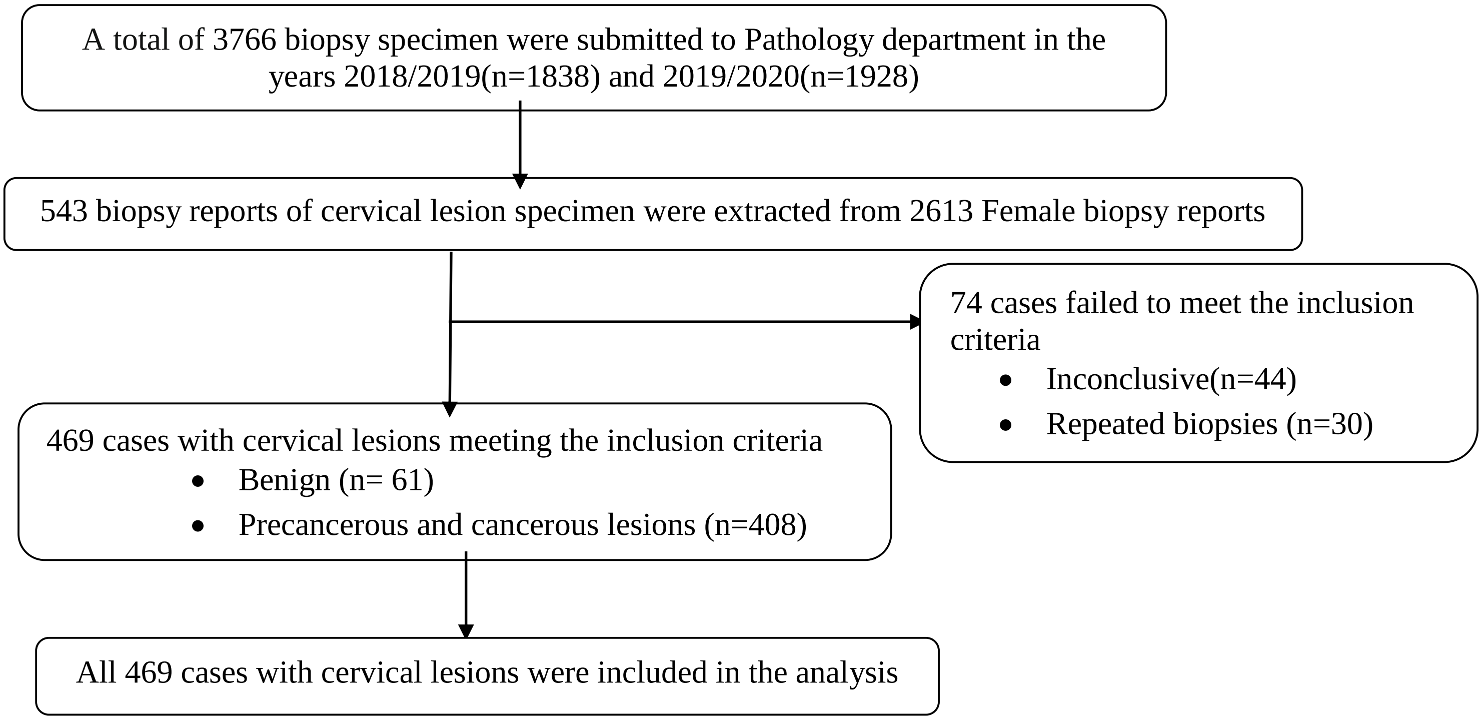

Figure 1: Diagram showing the sampling procedure of the selected 469 cervical lesion biopsy records from 2018 to 2020

3.6. Study variables

3.6.1. Dependent variable

- ❖ Histopathologic pattern of Cervical tissue specimens

3.6.2. Independent variable

- ♠ Age
- ♠ Place of Residence
- ♠ Type of biopsy
- ♠ Clinical features

3.7 Data collection procedures

Histopathology reports of biopsies submitted from cervical lesions that were routinely processed and from which paraffin sections were taken and stained with hematoxylin and eosin (H&E) for microscopic examination and histopathological diagnosis at JMC, pathology department, from September 12, 2018 to September 12, 2020, were retrieved from the pathology department data archive. Eligible 469 reports fulfilling inclusion and exclusion criteria were extracted and recorded into a prepared checklist containing study variables. All necessary preventive methods for coronavirus disease-19 (COVID-19) were used throughout as per the national protocol by using personal protective equipment, physical distancing, and disinfectants.

3.8 Data Processing and Analysis

Data were cleaned, coded, entered into EpiData v3.1, and exported to SPSS version 26 for analysis. Cross tabulation, the chi-square test, and logistic regression with multivariate analysis were performed to look for associations between the study variables. Variables with P values less than 0.5 were chosen for multivariable logistic regressions. A p-value of 0.05 was used as a cutoff point for identifying predictors for histopathologic patterns. The findings were presented using text, tables, and charts.

3.9 Data quality management

A checklist was adopted after reviewing different kinds of literature and books, and the checklist was pretested on 47 cases (10% of the total sample size) of hard-copy biopsy reports done in the year 2017 that were not included in the current study. Then the checklist was revised with some modifications to the variable, and the final revised checklist was used for data collection. Two days of training were given to the data collectors on how to locate, retrieve, categorize, and record the data, and initial data collection was accompanied by the principal investigator. The principal investigator subsequently followed and supervised while the data collectors were retrieving and recording the biopsy results from the pathology department data archive using checklists.

Following a thorough review of the checklist, data were entered into Epidata on a password-protected computer and exported to SPSS version 26 for analysis.

3.10 Ethical consideration

Before data collection, the proposal for this study was submitted to Jimma University's research and ethical committee. Then, ethical clearance for the use of secondary data and a waiver of informed consent was obtained from the Institutional Review Board (IRB) of JMC and submitted to the responsible authorities of JMC. A formal letter of cooperation was also written to obtain permission from the head of the pathology department before using the data. All the information collected from the study was handled confidentially by omitting the personal identification of the pathology reports, and data utilization was carried out in accordance with the ethical and scientific standards outlined in national and international guidelines.

3.11 Operational Definitions (1,5)

Cervical tissue specimens: specimens of patients with cervical complaints such as punch biopsies and hysterectomy specimens.

Cancerous cervical lesion: malignant lesions of the cervix.

Histopathologic pattern: a specific type of diagnosis made on biopsy specimens.

Noncancerous cervical lesion: benign conditions of the cervix.

Precancerous cervical lesion: precursor lesions of cervical cancer (LSIL and HSIL)

3.12 Limitations of the Study

Biopsy results are not computerized and compiled with the clinical data of patients; thus, most of the factors associated with cervical cancer cannot be assessed. Confirmatory immunohistochemistry and HPV DNA testing could not be included in this study because of the limited setup. Power analysis for sample size calculation was not performed.

4. Results

In 2018–2019 (n = 1838) and 2019–2020 (n = 1928), a total of 3766 biopsy specimens (2613 female and 1153 male) were submitted to the pathology department. From 2613 female biopsy reports, 543 biopsy reports of cervical lesion specimens were extracted, of which 74 were excluded because they fulfilled the exclusion criteria. Cervical biopsy specimens accounted for 14.4% of the total biopsy specimens and 20.8% of the total biopsy specimens from female patients who submitted biopsy specimens during the study period.

4.1. Sociodemographic profiles

Of the 469 cervical lesion cases, 244 cases (52%) were from 2018–2019 and 225 (48%) cases were from 2019–2020 (Figure 2).

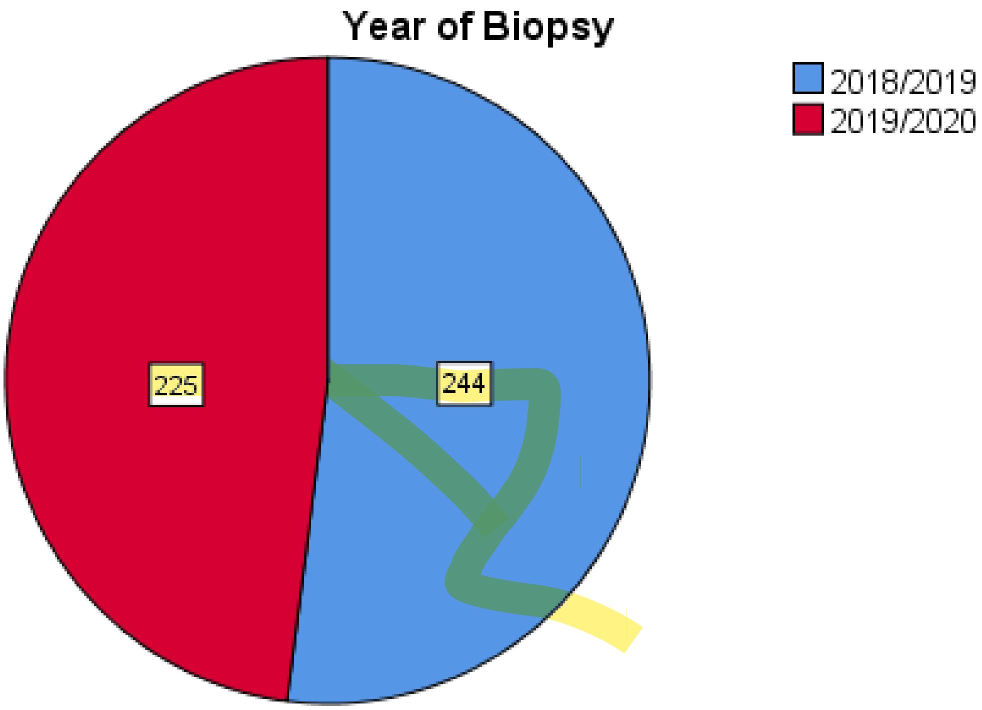

Figure 2: Pie chart showing the distribution of cervical lesions between 2018 and 2020

The age distributions have a minimum value of 22 years and a maximum value of 85 years, with a mean age of 47.06 years and a standard deviation of 11.019 years. The maximum age distribution of cervical lesions was in the 41–50-year age range, accounting for 154 (32.8%) biopsies, followed by 31–40 years with 143 (30.5%) biopsies, and 51–60 years with 102 (21.7%) biopsies.

More than half of precancerous and cancerous cervical lesions (65.46%) occurred after the age of 40 years, while benign lesions accounted for 47.5% after the age of 40 years. This was statistically significant. (X^2=23.54, DF =5 p=**0.001**).

As the patient's age increases, the risk of precancerous and cancerous cervical lesions increases, and the third and fourth decades of life were found to be associated with an increased risk of having precancerous and cancerous cervical lesions (Figure 3).

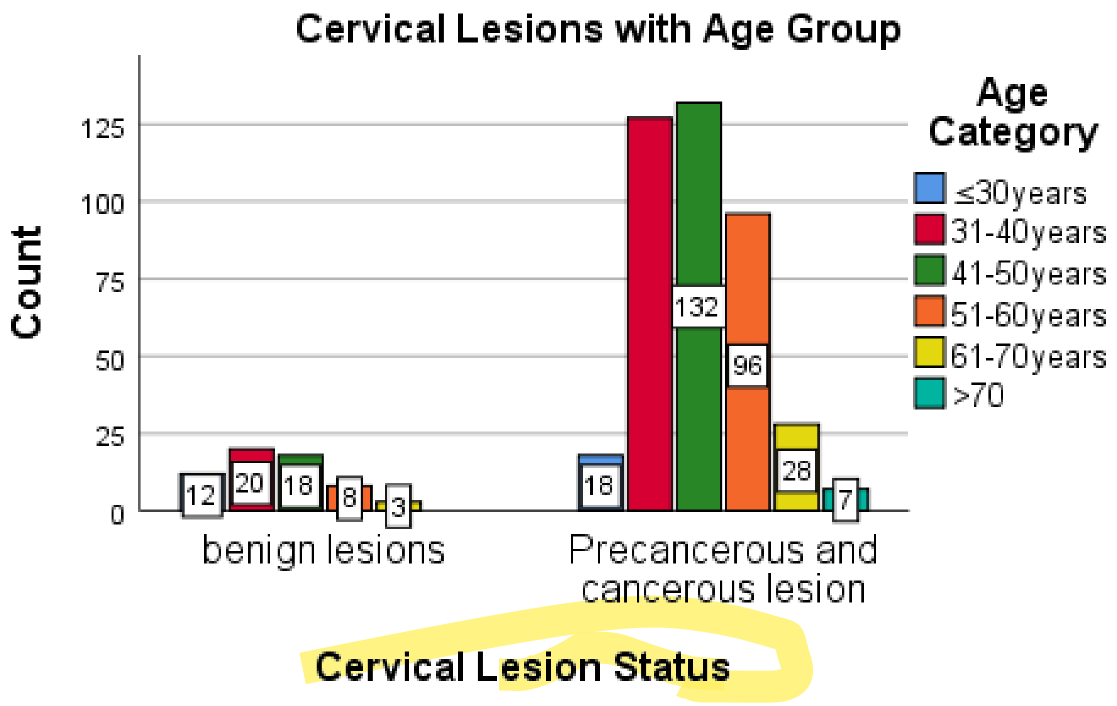

Figure 3: Bar graph showing cervical lesion status with age groups

Most of the patients (77.8%) were from surrounding areas with variable distances from Jimma town, while 104 (22.2%) were from Jimma town. The majority of patients coming from the periphery were from the Jimma zone, accounting for 58.4% (Figure 4). Most (88.8%) of the cases from the surrounding areas of Jimma were precancerous and cancerous cervical lesions, while 80.8% of the cases in Jimma town were precancerous and cancerous cervical lesions. This showed a strong association between precancerous and cancerous cervical lesions and residency. (X2 = 4.67, DF = 1, p = 0.027).

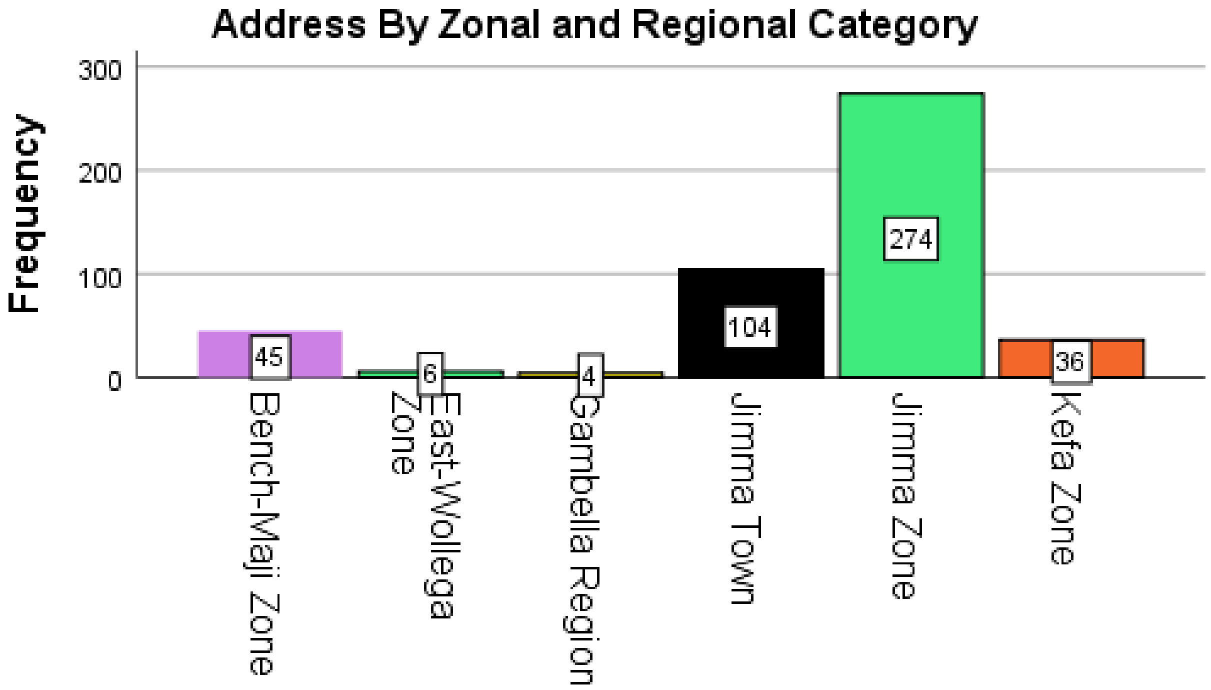

Figure 4: Bar graph showing the distribution of cervical lesions among residents of Jimma town and surrounding areas

4.2. Clinical data of patients with cervical lesions

The most common presenting symptom of patients with cervical lesions was vaginal bleeding alone, accounting for 206 (43.9%) patients, followed by vaginal bleeding with vaginal discharge (108 (23%) patients) and vaginal bleeding with lower abdominal pain (89 (19%) patients). (Table 1). The duration of symptoms in patients with cervical lesions ranged from four days up to sixteen years (Figure 5). The most common type of biopsy performed for patients with cervical lesions was a punch biopsy, accounting for 72.9% (Table 2). Most (90%) patients with cervical complaints were clinically diagnosed with cervical cancer (Figure 6)

Table 1: Clinical features/presentation of patients with cervical lesions

Clinical features/presentation	Frequency	Percent
Vaginal Bleeding	226	48.2
Vaginal Bleeding and Vaginal Discharge	108	23.0
Vaginal Bleeding and Lower abdominal pain	89	19.0
Vaginal Discharge	44	9.4
Cervical CA Screening	2	0.4
Total	469	100.0

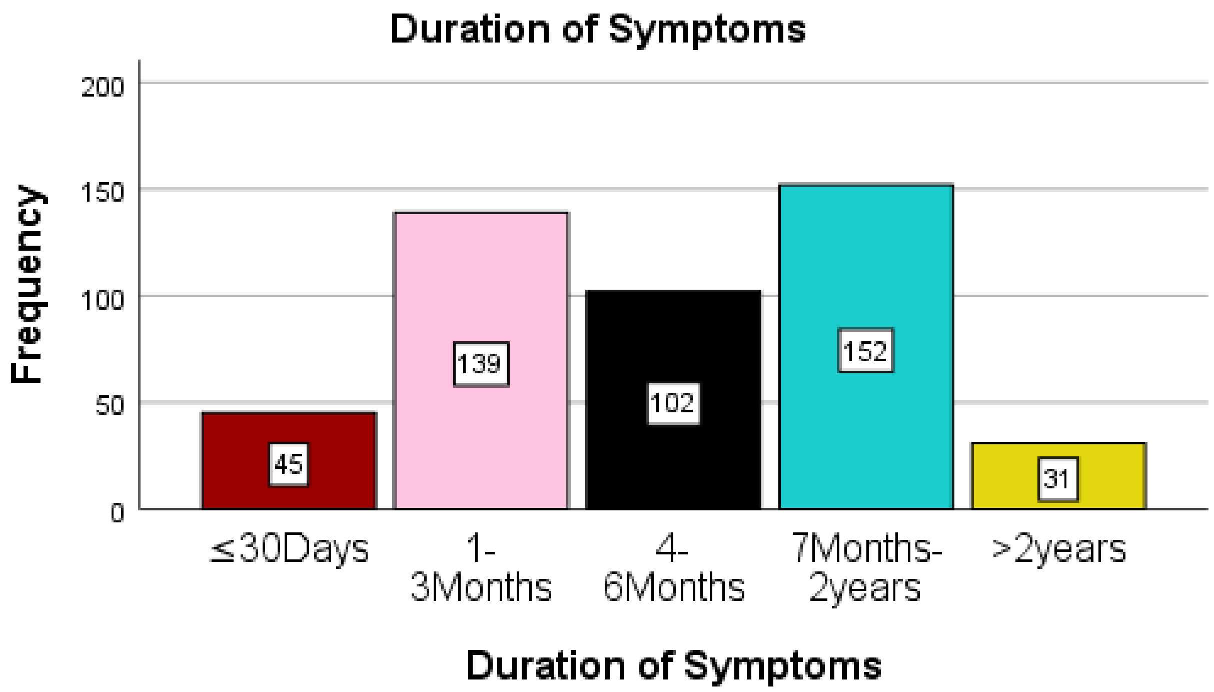

Figure 5: Bar graph showing the distribution of the duration of symptoms in patients with cervical lesions

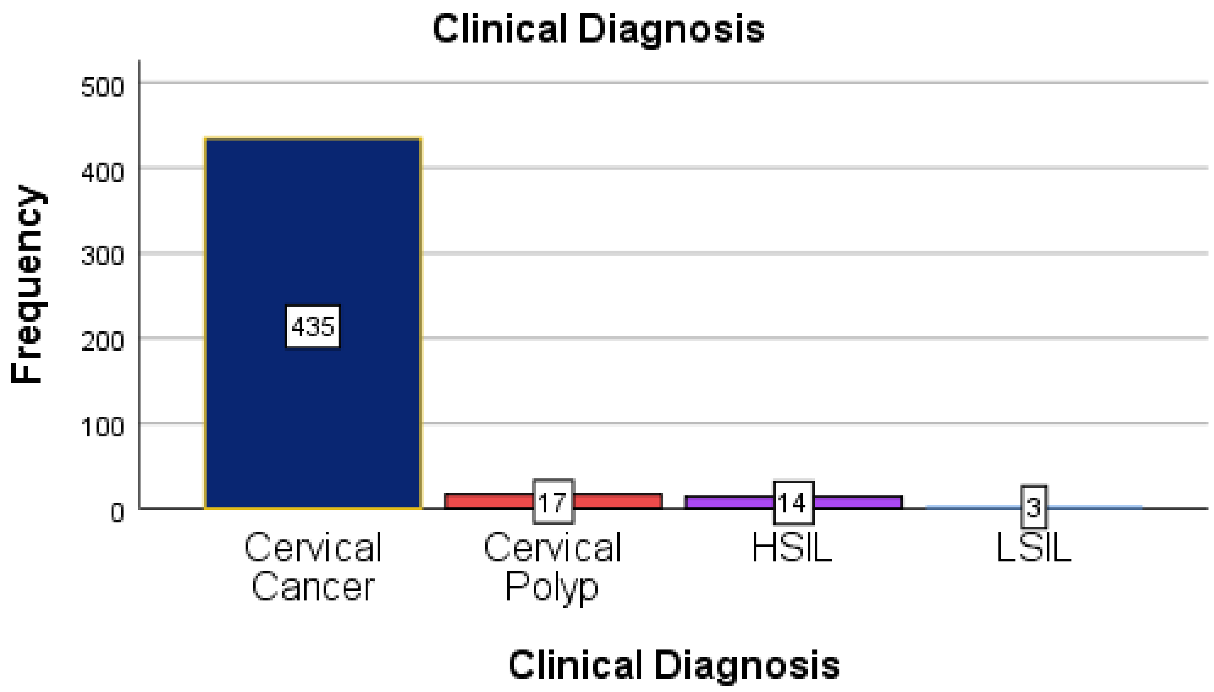

Figure 6: Bar graph showing the distribution of clinical diagnosis of patients with cervical lesions

Table 2: Type of biopsy of patients with cervical lesions

Type of Biopsy/Nature of specimen	Frequency	Percent
Cone Biopsy	3	0.6
Hysterectomy	30	6.4
LEEP	8	1.7
Punch Biopsy	428	91.3
Total	469	100.0

4.3. Histopathologic patterns of cervical lesions

Most (87%) of the specimens were precancerous and cancerous cervical lesions, while 13% were benign cervical lesions (Figure 7). The majority (71%) of cases were cancerous cervical lesions, followed by precancerous cervical lesions (16.4%) (Table 3). Squamous cell carcinoma (SCC) was the most frequent cervical cancer diagnosed during the study period, accounting for 96.4% (Table 4). HSIL was the most frequently diagnosed precancerous lesion, accounting for 68.4%. Endocervical polyps were the most commonly diagnosed benign lesions, accounting for 59.3% (Table 3). The most common histologic subtype of SCC was keratinizing SCC, accounting for 77.4% of all cases, while the remaining 22.6% were nonkeratinizing SCC (Figure 8).

Table 3: Numbers and percentages of cervical lesions

**Type of Cervical Lesion**	**Frequency**	**Causes**	**Percentage**
Precancerous cervical lesion	79	54(68.4%) HSIL<br>25(31.6%) LSIL	16.8%
Cancerous cervical lesion	331	See Table 4	70.6%
Noncancerous cervical lesion	59	35(59.3%) Endocervical polyp<br>18(30.5%) Squamous metaplasia<br>6(10.2%) Cervicitis	12.6%
Total	469		100%

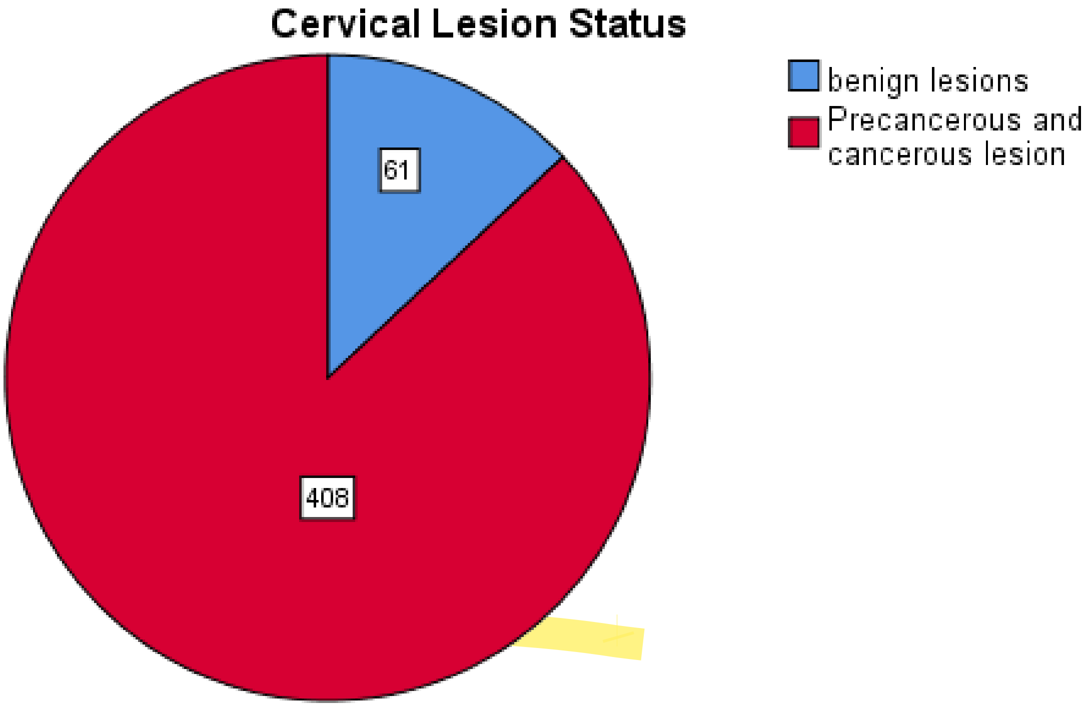

Figure 7: Pie chart showing the distribution of cervical lesion status

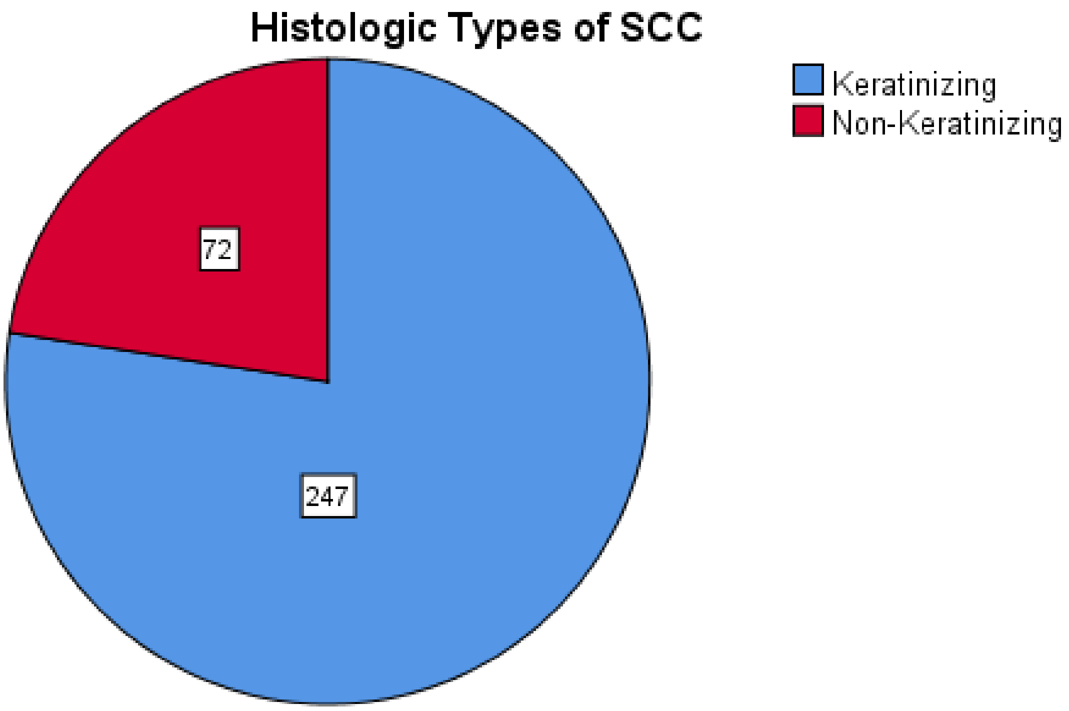

Figure 8: Pie chart showing histologic types of SCC

Table 4: Histopathological classification of cervical cancer

Histopathologic type	Frequency	Percentage
Squamous cell carcinoma	319	96.4%
Adenocarcinoma	11	3.3%
Adenosquamous carcinoma	1	0.3%
Total	331	100%

Cancerous cervical lesions tend to develop in older age groups, and benign conditions tend to develop in younger age groups. SCC occurred most frequently in the 41–50-year age group (N = 104), followed by the 31-40 year (N = 86) and 51-60 year age groups (N = 85). (Table 5)

Table 5: Pattern of the cervical lesion by age group

Age	Histopathologic Diagnosis								
Group	Cervicitis	Squamous Metaplasia	Endocervical Polyp	LSIL	HSIL	Squamous Cell Carcinoma	Adenocarcinoma	Adenosquamous Carcinoma	Total
20-30years	0	4	8	3	1	14	0	0	30
31-40years	2	6	13	11	23	86	1	1	143
41-50years	3	3	10	10	19	104	5	0	154
51-60years	0	3	4	1	6	85	3	0	102
>60years	1	2	0	0	5	30	2	0	40
Total	6	18	35	25	54	319	11	1	469

4.4. Factors associated with cervical lesions

Age (*p*=**0.001, COR=1.048**) and place of residence (*p*= **0.032, COR=0.531**) were tested at a P value less than 0.05 in bivariate logistic regression and selected as candidate variables for multivariate logistic regression. Cervical lesions were grouped into benign and HPV-related precancerous and cancerous lesions. Addresses were categorized into Jimma town, which is urban and surrounding areas, which were rural. Multivariate logistic regression analysis was performed on these variables using the step-wise method and the overall model was significant at a P value of 0.001. Age (*p*=**0.001, AOR=1.052**) and place of residency (*p*=**0.015, AOR=0.47**) were shown to be the independent predictors of precancerous and cancerous cervical lesions, hence older women are 1.052 times more likely to develop precancerous and cancerous cervical lesions than younger women and rural dwellers are 0.47 times more likely to develop precancerous and cancerous cervical lesions than urban dwellers which were statistically significant as shown in Tables 6,7 and 8.

Table 6: Bivariate logistic regression of cervical lesion status and age

Independent Variable	B-Coefficient	t-value	P-value	R square	COR
Age	0.154	3.369	0.001	0.024	1.048

COR: -Crude Odds Ratio

Table 7: Bivariate logistic regression of cervical lesion status and address

Independent Variable	Beta Coefficient	t-value	P-value	R square	COR
Address	0.099	2.145	0.032	0.010	0.531

COR: -Crude Odds Ratio

Table 8: Multivariate logistic regression of cervical lesion status and associated factors

Independent Variables	Beta Coefficient	t-value	P-value	AOR	R square
Age	0.162	3.561	0.001	1.052	0.036
Address	0.111	2.435	0.015	0.47	

AOR:- Adjusted Odds Ratio

5. Discussion

Cervical lesions tend to affect females across a wide age range. The age distributions have a minimum value of 22 years and a maximum value of 85 years, with a mean age of 47.06 years and a standard deviation of 11.019 years. This is consistent with Ameya G.'s study on 513 biopsies in Hawassa, Ethiopia, in which the age ranges were 17 to 85 years, with a mean and standard deviation of 42 and 11 years respectively (18). Our study is also similar to a study done on 306 cervical cancer cases in Nigeria, which showed patient ages diagnosed with cervical cancer to range from 18 to 85 years, with peak occurrence in the fifth decade (19). A study done on 1047 specimens in Lagos, Nigeria, also showed the age range of patients as 19–87 years, with a mean age of 49±13 (20). Another study done at TASH, A.A., Ethiopia on women with histologically verified cancer of the cervix uteri also showed that the mean age was 49 years (21–91 years) (21). Our findings are consistent with this and other similar studies.

Cervical lesions range from benign inflammatory conditions and polyps to malignant conditions. Of the 469 biopsies submitted to JMC, the department of pathology, most (87%) of the specimens were precancerous and cancerous cervical lesions, while 13% were benign cervical lesions. The majority (70.6%) of cases were cancerous cervical lesions, followed by precancerous cervical lesions (16.8%) and noncancerous cervical lesions (12.6%). A study performed in Malawi on 212 cervical lesions showed that 17% had precancerous lesions, 65% were cancerous, and 18% had both precancerous and cervical cancer, making a total of 83% cancerous lesions (22). Another study performed in India on 110 cases showed that a diagnosis of intraepithelial neoplasia was made in 32.7% of cases, and invasive carcinoma was found in 67.3% of cases (23). Therefore, the finding of our study has a slightly higher number of cancerous cases than this last finding and other similar studies. This is possibly because of the limited resources and services within the study area.

Squamous cell carcinoma (SCC) was the most frequent cervical cancer diagnosed during the study period, accounting for 96.4% of the 333 cancerous cases, followed by adenocarcinoma (3.3%) and adenosquamous carcinoma (0.3%). This is consistent with a study done at JUSH, Jimma, Ethiopia, on 154 cervical cancer cases, which showed that 91% of the cases were SCC, 5.84% were small cell carcinomas, 2.59% were adenocarcinomas, 0.64% were adenosquamous carcinomas (24). Our study is also consistent with a study performed in Cameron on 2078 cervical cancer cases, which

showed that the most common cervical cancer was SCC (81.18%), followed by adenocarcinoma (12.95%) (25). In another study done at TASH, Addis Ababa, Ethiopia, cancer of the uterine cervix was found to be the most common malignancy among all gynecological malignancies (26). Another study done at A.A., Ethiopia on women with postmenopausal bleeding also showed that about 94% of the cervical malignancies were squamous cell carcinoma(27). A study done at Saint Paul Hospital Millennium Medical College (SPHMMC), Addis Ababa, Ethiopia, on breast and gynecologic malignancies also showed that SCC of the cervix was the most common type of cervical malignancy detected, accounting for 90% of all cases, while adenocarcinoma (3.85%) was the second most observed type of cervical cancer (28). Similarly, according to a study done in Bangladesh on gynecologic malignancy, SCC was the most common histopathologic type in cervical and vulvar cancers (29). Another study done in Sudan on 372 patients showed that SCC (NOS) was the most common subtype (76.6%) and adenocarcinoma was the second most common subtype (12.6%) (30).

On the contrary, according to a study performed in India over a period of 2 years, the most frequent condition diagnosed on cervical biopsies and hysterectomy specimens was benign cervical polyp (66.3%), followed by carcinoma (23.7%) and squamous intraepithelial lesions (0.2%)(31). These last studies and other studies performed in Western countries show a higher number of benign cervical lesions because of early screening and the detection of precursor lesions. Although cervical cancer can be prevented by vaccination and screening and cured if identified at an early stage, this is not yet fully implemented in the study area.

Of the 79 precancerous cervical conditions diagnosed during the study period, HSIL was the most frequently diagnosed precancerous lesion, accounting for 68.4%, with the other 31.6% being LSIL. This is consistent with a study performed in Hawassa, Ethiopia, on 513 biopsies, which showed that among precancerous cervical lesions, HSIL was the predominant lesion (39.8%) (18). Similarly, a study performed in India showed that cervical intraepithelial neoplasia (CIN) 1 was diagnosed in 36.1% of patients, followed by CIN 2 (33.3%) and CIN 3 (30.6%) (23).

Of the 59 benign cases, endocervical polyps were the most commonly diagnosed benign lesion, accounting for 59.3%, followed by squamous metaplasia (30.5%) and cervicitis (10.2%). A study performed in Nigeria on 176 cervical specimens showed that inflammatory lesions accounted for 55 cases (59.8%) of non-neoplastic cervical lesions, followed by endocervical polyps, which accounted for 15 cases (16.3%) of non-neoplastic cervical lesions. Among the inflammatory lesions, chronic nonspecific cervicitis was the most commonly encountered lesion, constituting 40 cases (72.2%) of all inflammation (32). In a study performed at TASH, Addis Ababa, Ethiopia chronic cervicitis was the commonest benign lesion (16.8%) (33). Contrary to this and other similar studies, our study has the lowest number of cervicitis cases, possibly because the majority of cervicitis cases are managed clinically without histopathological confirmation once a clinical diagnosis is made as per the National STD Guidelines.

6. Conclusion

Cervical diseases include a wide range of disorders and occur across different age groups. The age distributions have a minimum value of 22 years and a maximum value of 85 years, with a mean age of 47.06 years. The most common presenting symptom in patients with cervical lesions was vaginal bleeding. The most common type of biopsy performed for patients with cervical lesions is a punch biopsy. Most (90%) patients with cervical complaints were clinically diagnosed with cervical cancer. Cervical lesions range from benign inflammatory conditions and polyps to malignant conditions. Most cervical lesions were precancerous and cancerous. Squamous cell carcinoma (SCC) was the most frequent type of cervical cancer. HSIL was the most frequently diagnosed precancerous cervical lesion. Endocervical polyps were the most commonly diagnosed benign cervical lesion. Precancerous and cancerous cervical lesions tend to increase with increasing age and in those patients who come from peripheral areas.

7. Recommendation

There was a rising burden of cervical cancer. This implies that there is a need to educate the community to improve health-seeking behavior and on possible preventive strategies for cervical cancer. It is also strongly recommended that JMC's Department of Pathology commence cervical cytological examinations to detect dysplasia before it turns into full-blown cancer. We recommend that the Federal Ministry of Health strengthen the ongoing nationwide HPV vaccinations, screenings, and interventions for cervical cancer at a much faster rate.

8. Declarations

Ethical consideration

Before data collection, the proposal for this study was submitted to Jimma University's research and ethical committee. Then, ethical clearance for the use of secondary data and a waiver of informed consent was obtained from the Institutional Review Board (IRB) of JMC and submitted to the responsible authorities of JMC. A formal letter of cooperation was also written to obtain permission from the head of the pathology department before using the data. All the information collected from the study was handled confidentially by omitting the personal identification of the pathology reports, and data utilization was carried out in accordance with the ethical and scientific standards outlined in national and international guidelines.

Institutional consent

To: -JUMC, Department of Pathology

My name is Dr. Birhanu Hailu Tirkaso, a final-year pathology resident at Jimma University. I am doing research titled "Histopathologic patterns and factors associated with cervical lesions at Jimma Medical Center, Jimma, Southwest Ethiopia: a 2-year cross-sectional study" as a partial fulfillment of the requirement for a specialty in human anatomic pathology. The objective of this research is to determine the histopathologic patterns of patients with cervical lesions and associated factors at Jimma Medical Center. The research requires the use of secondary data from histopathology reports of biopsies submitted from cervical lesions at JMC's pathology department in the years 2018–2019 and 2019–2020. Data will be collected from July 1 to July 31, 2020. I kindly request the Department of Pathology, JMC, to grant me permission for the collection of the secondary data for the specified period and for publication in the appropriate journal.

Acknowledgment

I thank Jimma University for allowing me to undertake the research. I extend my gratitude to Jimma Medical Center, Department of Pathology, for providing me with the necessary resources. I also thank my advisors and friends for giving me constructive comments and important information to prepare my proposal. Last but not least, my warm gratitude goes to all my family members for providing me with emotional support during this study.

Consent to publish

Not applicable.

Competing interests

I declare that I have no conflicts of interest.

Authors' contributions

Dr. Birhanu Hailu Tirkaso, as the principal investigator, conducted the research and prepared the manuscript, including the text, figures, and tables. Dr. Tesfaye Hurgesa Bayisa and Dr. Tewodros Wubshet Desta reviewed the manuscript.

Funding

The author received no financial support for the research, authorship, and/or publication of this research.

Availability of data and materials

The data that support the findings of this study are available from the corresponding author, upon reasonable request.

Disclosure

This paper is based on the thesis of Birhanu Hailu, the corresponding author. It has been published on the institutional website: https://repository.ju.edu.et/handle/123456789/4080.

References

1. John Goldberg, McKenney. "Rosai and Ackerman's Surgical Pathology" 11th eds. In: 11th ed. Philadelphia: Elsevier; 2018. p. 1260–93.
2. Kumar V., Abbas K. AJ. Robbins, and Cotrans Pathologic Basis of Diseases 9th ed. 9th editio. Philadelphia: Elsevier; 2015. 1001–1007 p.
3. Robboy SJ. Robboy's Pathology of the Female Reproductive Tract [Internet]. Elsevier Health sciences; 2009 [cited 2023 Jun 6]. 1001–1007 p. Available from: https://books.google.com.et/books?hl=en&lr=&id=ab545XL-MBEC&oi=fnd&pg=PR29&dq=3.+Robboy+S.,+Mutter+G.,Prat+J.,Et+al.+Robboy%27s+Pathology+of+Female+Reproductive+Tract.+2nd+Edition.+Philadelphia+:+Elsevier,+2009,+pp.+189-295.&ots=d4zyLlxZJo&sig=kp7dnNNhqn3zRa944GvDuN5EvYs&redir_esc=y#v=onepage&q&f=false
4. Fletcher CDM. Diagnostic Histopathology of Tumors. In: 4th ed. Philadelphia: Elsevier; 2013 [cited 2023 Jun 6]. p. 814–38. Available from: https://shop.elsevier.com/books/diagnostic-histopathology-of-tumors-2-volume-set/fletcher/978-0-323-42860-6
5. Herrington CS, Board WC of TE. Female Genital Tumours [Internet]. WHO Female Genital Tumours. 2020 [cited 2023 Jun 6]. 632 p. Available from: https://www.research.ed.ac.uk/en/publications/who-classification-of-tumours-female-genital-tumours
6. Ikechebelu JI, Onyiaorah I V., Ugboaja JO, Anyiam DCD, Eleje GU. Clinicopathological analysis of cervical cancer seen in a tertiary health facility in Nnewi, south-east Nigeria. http://dx.doi.org/103109/01443610903531394 [Internet]. 2010 Apr [cited 2023 Jun 6];30(3):299–301. Available from: https://www.tandfonline.com/doi/abs/10.3109/01443610903531394
7. Ethiopian Federal Ministry of Health. FEDERAL MINISTRY OF HEALTH ETHIOPIA DISEASE PREVENTION AND CONTROL DIRECTORATE NATIONAL CANCER CONTROL PLAN [Internet]. 2016 [cited 2023 Jun 6]. Available from: https://www.google.com/url?sa=D&q=https://www.iccp-portal.org/sites/default/files/plans/NCCP%2520Ethiopia%2520Final%2520261015.pdf&ust=1686138720000000&usg=AOvVaw0eDMP3rQOPzm9DhCUtnBPJ&hl=en&source=gmail
8. Ferlay J, Shin HR, Bray F, Forman D, Mathers C, Parkin DM. Estimates of worldwide burden of cancer in 2008: GLOBOCAN 2008. Int J Cancer [Internet]. 2010 Dec 15 [cited 2023 Jun 6];127(12):2893–917. Available from: https://onlinelibrary.wiley.com/doi/full/10.1002/ijc.25516
9. Ferlay J, Colombet M, Soerjomataram I, Mathers C, Parkin DM, Piñeros M, et al. Estimating the global cancer incidence and mortality in 2018: GLOBOCAN sources and methods. Int J Cancer. 2019 Apr 15;144(8):1941–53.
10. Ferlay J, Soerjomataram I, Dikshit R, Eser S, Mathers C, Rebelo M, et al. Cancer incidence and mortality worldwide: Sources, methods and major patterns in GLOBOCAN 2012. Int J Cancer. 2015 Mar 1;136(5):E359–86.
11. Information Centre on HPV and Cancer. Human Papillomavirus and Related Diseases Report WORLD [Internet]. Available from: www.hpvcentre.net

12. Deressa BT, Cihoric N, Badra EV, Tsikkinis A, Rauch D. Breast cancer care in northern Ethiopia – cross-sectional analysis. BMC Cancer [Internet]. 2019 Apr 25 [cited 2023 Jun 6];19(1). Available from: /pmc/articles/PMC6485046/
13. Memirie ST, Habtemariam MK, Asefa M, Deressa BT, Abayneh G, Tsegaye B, et al. Estimates of cancer incidence in Ethiopia in 2015 using population-based registry data. J Glob Oncol. 2018 Mar 1;2018(4):1–11.
14. Ntekim A, Ntekim A. Cervical Cancer in Sub Sahara Africa. Topics on Cervical Cancer With an Advocacy for Prevention [Internet]. 2012 Mar 2 [cited 2023 Jun 6]; Available from: https://www.intechopen.com/chapters/30747
15. Census 2007. Stastical Oromia [Internet]. [cited 2023 Jun 6]. Available from: http://www.csa.gov.et/census-report/complete-report/census-2007?start=5
16. Jimma University. Home – JU Official Website [Internet]. [cited 2023 Jun 6]. Available from: https://ju.edu.et/
17. Mapcarta. Jimma University Map - University - Ethiopia - Mapcarta [Internet]. [cited 2023 Jun 6]. Available from: https://mapcarta.com/33099124
18. Ameya G, Yerakly F. Characteristics of cervical disease among symptomatic women with histopathological sample at Hawassa University referral hospital, Southern Ethiopia. BMC Womens Health [Internet]. 2017 Sep 29 [cited 2023 Jun 6];17(1):1–7. Available from: https://link.springer.com/articles/10.1186/s12905-017-0444-5
19. Silas OA, Yakubu D, Jegede OO, Ajetunmobi OI, Mosugu O. A histopathologic review of cervical cancers in Jos University Teaching Hospital, Jos, Nigeria. Annals of Tropical Pathology [Internet]. 2018 [cited 2023 Jun 6];9(2):135. Available from: https://www.atpjournal.org/article.asp?issn=2251-0060;year=2018;volume=9;issue=2;spage=135;epage=138;aulast=Silas
20. Faduyile F, Soyemi S, Wright K, Osuolale F. Histopathological study of surgical cervical biopsies in Lagos, Nigeria. Trop J Obstet Gynaecol. 2017;34(2):124.
21. Kantelhardt EJ, Moelle U, Begoihn M, Addissie A, Trocchi P, Yonas B, et al. Cervical Cancer in Ethiopia: Survival of 1,059 Patients Who Received Oncologic Therapy. Oncologist [Internet]. 2014 Jul 1 [cited 2023 Jun 6];19(7):727–34. Available from: https://academic.oup.com/oncolo/article/19/7/727/6399365
22. Bangara FF, Marimo C, Kaile T, Simakando M, Tomoka T, Arabi G, et al. Histological Characterization of Invasive Cervical Cancer and Precursor Lesions in Malawian Women Presenting at Queen Elizabeth Central Hospital (QECH): A Cross-Sectional Study. J Virol Antivir Res. 2017;06(01).
23. Mamta Gupta PB. Histopathological Spectrum of Premalignant and Malignant Lesions of Uterine Cervix. National Journal of Laboratory Medicine [Internet]. 2018 [cited 2023 Jun 6];7(1). Available from: DOI: 10.7860/NJLM/2018/32340:2276
24. Saleem A, Bekele A, Fitzpatrick MB, Mahmoud EA, Lin AW, Velasco HE, et al. Knowledge and awareness of cervical cancer in Southwestern Ethiopia is lacking: A descriptive analysis. PLoS One [Internet]. 2019 Nov 1 [cited 2023 Jun 6];14(11):e0215117. Available from: https://journals.plos.org/plosone/article?id=10.1371/journal.pone.0215117
25. Paul ENJ, Charlotte TN, Jules OML, Godefroy S, Daniel ESJ, Michelle EFA, et al. Epidemiological and Histological Profile of Cervical Cancer in Cameroon: About 2078 Cases. Open J Obstet Gynecol [Internet]. 2016 [cited 2023 Jun 6];06(04):232–9. Available from: http://file.scirp.org/Html/

26. Tigeneh W, Molla A, Abreha A, Assefa M. Pattern of Cancer in Tikur Anbessa Specialized Hospital Oncology Center in Ethiopia from 1998 to 2010. Int J Cancer Res Mol Mech [Internet]. 1998;1. Available from: http://dx.doi.org/10.16966/2381-3318.103
27. Ergete W, Tesfaye A, Health EJ. The Ethiopian Journal of Health Development Histopathological findings of Post-Menopausal bleeding in Ethiopian women.
28. Hailu HE, Mondul AM, Rozek LS, Geleta T. Descriptive Epidemiology of breast and gynecological cancers among patients attending Saint Paul's Hospital Millennium Medical College, Ethiopia. PLoS One [Internet]. 2020 [cited 2023 Jun 6];15(3):e0230625. Available from: https://journals.plos.org/plosone/article?id=10.1371/journal.pone.0230625
29. Rahman MA, Siddika ST, Mazid M. Gynaecological Cancers in Surgical Specimens – A Hospital Based Analysis. Med Today. 2015 Jul 16;26(2):78–82.
30. Rayis DAM, Zulfu A, Yassin K, Merghani A. Histopathological pattern of gynaecological malignancies: National Health Laboratory (NHL), Sudan. GPH - International Journal of Health Sciences and Nursing [Internet]. 2018 Aug 30;1(1). Available from: http://gphjournal.org/index.php/hsn/article/view/45
31. Atul J. RJ, IE al. Histopathological study of tumors of Cervix at MGM Medical College. Medical College of India. 2014;1(1).
32. Forae G, Nwachokor F. Morphological spectrum of non-neoplastic lesions of the uterine cervix in Warri, South-South, Nigeria. Niger J Clin Pract. 2013;16(4):429.
33. Nigatu B, Gebrehiwot Y, Kiros K, Ergete W. Number 1 Ethiopian Journal of Reproductive Health.

Manuscript Click here to access/download;Manuscript;Manuscript.docx

Histopathologic patterns and factors associated with cervical lesions at Jimma Medical Center, Jimma, Southwest Ethiopia: a two-year cross-sectional study

Birhanu Hailu Tirkaso1*,
Tesfaye Hurgesa Bayisa2, and
Tewodros Wubshet Desta3

1Department of Biomedical Sciences, Mizan-Tepi University, Mizan, Southwestern Region, Ethiopia

2Department of Pathology, Jimma University, Jimma, Oromia, Ethiopia

3Department of Pathology, Dire Dawa University, Dire Dawa, Ethiopia

**Corresponding e-mail: birhanuhailu@mtu.edu.et*

Abstract

Background: The cervix is the lower portion of the uterus, which connects this organ to the vagina through the endocervical canal.

Objective: This study aimed to determine the histopathologic patterns and factors associated with cervical lesions at Jimma Medical Center from September 12, 2018, to September 12, 2020.

Methods: A 2-year facility-based cross-sectional study was conducted from May 1 to June 30, 2019.

Result: In this study, cervical cancer was the most common (71%) cause of cervical lesions. Squamous cell carcinoma was the most frequent cervical cancer diagnosed during the study, accounting for 96.4% of 331 cancerous cases, followed by adenocarcinoma (3.3%). High-grade squamous intraepithelial lesions were the most frequently diagnosed precancerous lesions, accounting for 68.4% of cases. Endocervical polyps were the most commonly diagnosed benign lesions, accounting for 59.3% of cases.

Conclusion: The maximum age distribution of cervical lesions was in the 41–50-year age range. Squamous cell carcinoma was the most frequent type of cervical cancer. High-grade squamous intraepithelial lesions were the most frequently diagnosed precancerous cervical lesions. The most common benign cervical lesions were endocervical polyps.

Recommendation: We recommend educating the community to improve health-seeking behavior and on possible preventive strategies for cervical cancer.

Keywords: Histopathology, Cervical Lesions, Cervical Cancer, Jimma Medical Center, Ethiopia.

1. Introduction

1.1. Background

The endocervical canal connects the uterus to the vagina through the cervix, which is the bottom part of the uterus. Non-keratinizing squamous epithelium covers the majority of the exocervix. A layer of columnar mucus-secreting cells makes up the glandular mucosa of the endocervix, and the intersection between the squamous and glandular epithelia is called the squamocolumnar junction(1,2). The squamocolumnar junction undergoes eversion with the onset of puberty, causing columnar epithelium to become visible on the exocervix. The exposed columnar cells eventually undergo squamous metaplasia, forming a region called the transformation zone(3). The transformation zone is the area where the expected transformation to the metaplastic squamous epithelium and the abnormal transformation to CIN usually occur(4).

The range of neoplastic conditions of the cervix arises at or near the cervical transformation zone, ranging from precancerous intraepithelial neoplasia to epithelial malignancies, mixed epithelial and mesenchymal tumors, mesenchymal tumors, melanocytic tumors, and miscellaneous rare malignancies(5).

The epidemiology of precancerous lesions and squamous cell carcinoma (SCC) of the cervix follows the epidemiology of HPV infections. In developed countries, adenocarcinoma now makes up 10–25% of all cervical carcinomas, up from 5–10% thirty years ago. This rise is due to a decrease in squamous cell carcinomas brought on by screening programs and improved glandular lesions detection in cervical cytology samples. It is advised to employ a two-tier system of low- and high-grade intraepithelial lesions rather than the three-tier CIN 1, CIN 2, and CIN 3 terminology since it is more biologically applicable and histologically repeatable. The pairing of CIN 2 and CIN 3 as an HSIL is supported by our enhanced understanding of HPV biology. The typical type of SCC can be either keratinizing or nonkeratinizing (6).

According to a 5-year retrospective study done on 75 cases in Nigeria, cervical cancer has an incidence of 65.2% of all gynecological cancers and 13.4% of all gynecological admissions. The modal age was in the 60-70 years range. SCC of varying differentiation was the most common

histological type, accounting for 89.3 %, and adenocarcinoma accounted for only 8.0%. The modes of presentation were post-menopausal bleeding (84.0%), vaginal discharge (72.0%), contact bleeding (63.9%), and abdominal pain (56.2%)(7).

1.2. Problem statement

The overall burden of cancer is thought to be increasing globally, especially in developing nations. Thirteen million people will pass away from cancer and twenty-one million will receive a diagnosis by the year 2030. Even while mortality rates are just 8% to 15% higher in more industrialized nations, incidence rates for all malignancies combined are more than twice as high in more developed nations compared to less developed ones. This mismatch mostly reflects variations in diagnostic and therapeutic modalities' accessibility and availability. Similar to how tumors are more frequently found in the advanced stages in less developed nations, which contributes to the discrepancy in fatality rates compared to incidence. The National Cancer Control Plan (NCCP) of Ethiopia has established challenging goals to advance preventative intervention techniques, introduce screening exams for early detection, and provide chemotherapy, surgery, and palliative care in diagnosis and treatment. As seen in industrialized countries, it takes years for these measures to significantly reduce the incidence of cervical cancer (8).

Cervical cancer is one of the most common cancers in women, with approximately 0.5 million cases worldwide in 2008. With an average annual growth of 0.6%, cervical cancer cases grew from an estimated 378,000 in 1980 to 500,000 per year in recent years. Roughly 76% of recent cases occur in low-resource countries, with numbers rising everywhere but in high-income nations. In countries with greater affluence, the median age at death is 55. Effective programs to detect cancer precursors along with infrastructure to clinically manage precursor lesions are largely responsible for the significant differences in incidence and mortality between the more severely affected nations in Africa and Asia and the lower rates in wealthier North American and European countries (6) (9).

Cancer is one of the major non-communicable diseases (NCDs). Together with NCDs, cancer causes over 60% of total global mortality every year. Although communicable diseases still remain the leading killers, the incidence and mortality from NCDs are rising rapidly in many developing

countries. This has resulted in a double disease burden, which is putting strain on the existing health system (8) (10).

According to GLOBOCAN 2018, the global cancer burden has risen to 18.1 million cases and 9.6 million cancer deaths. One in six women and one in five men worldwide develop cancer during their lifetime, and one in eleven women and one in eight men die from the disease. The incidence of cancer worldwide is expected to rise to 26.4 million, with 17 million deaths by 2030 (11,12).

A 2-year retrospective study done on 3231 samples (1263 biopsies and 1968 FNAC) at UOG: North-West Ethiopia showed that 540 (16.7%) were malignant cancer cases. Cervical cancer and breast cancer were the two most common cancer types in the hospital, with cervical cancer ranking first (13).

In 2015, an estimated 21,563 and 42,722 incident cancer cases were diagnosed in males and females, respectively, according to a study based on primary data on 8539 patients from Addis Abeba's population-based cancer registry and supplemented by 1,648 cancer case data from six regions. Cervical cancer was the second most common and prevalent cancer, making up 23% of cancer cases, preceded by breast cancer (33%) (14).

Cervical cancer accounts for 22.5% of all cancer cases in women in sub-Saharan Africa, and the majority of women who develop cervical cancer live in rural areas. WHO estimated that cervical cancer will kill more than 443,000 women per year worldwide by 2030, nearly 90% of them in Sub-Saharan Africa (15).

1.3. Rationale of the Study

In Ethiopia, cervical cancer is the second most common female cancer. Throughout the world, HPV infections are the most common cause of cervical cancer. Out of more than 100 different HPV types, infection with HPV 16 and 18 has been associated with more than 70% of cervical cancers. Cervical cancer is a preventable and curable disease. It can be prevented by vaccination and screening and cured if identified early.

Despite the fact that high-risk HPV infection is a significant cause, only a small percentage of HPV-infected women develop invasive cancer, implying the presence of HPV infection co-factors that lead to cervical cancer development. According to studies carried out in different parts of Ethiopia, including the study area, knowledge about cervical cancer is very low. Although the NCCP launched nationwide HPV vaccinations in the 2016–2019 national cancer control plan, the effects take years to be seen in primary and secondary school students. Furthermore, PAP smears are only performed in a few centers across the country, and other health institutions in the country's outskirts perform a visual inspection with acetic acid (VIA). To my knowledge, no studies have included benign cervical conditions in the study area. The clinicopathologic studies done in the study area were conducted ten years ago.

1.4. Significance of the Study

This study augments the clinicopathologic studies done in southwestern Ethiopia from a histopathologic point of view, including benign cervical conditions. In addition, this study will serve as a baseline for future studies to be carried out at the national or continental level during the post-HPV vaccination era. The result of this study also serves as input for JMC, Zonal and Regional Health Bureaus, and the Ethiopian Federal Ministry of Health (EFMOH) in evaluating and monitoring the screening and preventive strategies.

2 Methods

2.1 Study area

The study was conducted at Jimma Medical Center, formerly called Jimma University Specialized Hospital (JUSH). JMC was established in 1938 in Jimma Town, located 352 kilometers southwest of Addis Ababa, the capital of Ethiopia. Jimma is the main town in southwestern Ethiopia with an estimated population of 120,960. It has a latitude of 7° 41′ 6′ ′ North, a longitude of 36° 49′ 53′ ′ East, and an elevation of 1738 meters (5702 feet). Currently, the hospital is a teaching and referral hospital in the southwestern part of the country, providing service for approximately 15,000 inpatients, 160,000 outpatient attendants, 11,000 emergency cases, and 4,500 deliveries in a year, coming from the catchment population of about 15 million (16–18).

The pathology department is one of the most important departments at JMC, providing services such as histopathology diagnostics, FNAC, fluid cytology, and hematopathology, with an annual average flow of 1,928 histopathology biopsy samples, over 5,000 FNAC, and over 200 BMA.

2.2 Study Design and Period

A 2-year facility-based cross-sectional study was conducted from May 1 to June 30, 2019.

2.3 Population

2.3.1 Target population

All female patients in the catchment area of Jimma Medical Center.

2.3.2 Source population

All female patients who submitted biopsy specimens to the pathology department for histopathology diagnosis from September 12, 2018 to September 12, 2020.

2.3.3 Study population

Histopathology reports of all female patients who submitted cervical tissue specimens to the pathology department from September 12, 2018, to September 12, 2020.

2.3.4 Study Unit

Histopathology reports of selected female patients who submitted cervical tissue specimens to the pathology department from September 12, 2018, to September 12, 2020, and fulfilled the inclusion criteria.

2.4 Inclusion and exclusion criteria

2.4.1 *Inclusion Criteria*

All female patients who submitted cervical tissue specimens to the pathology department from September 12, 2018 to September 12, 2020.

2.4.2 *Exclusion Criteria*

- Biopsy reports missing two or more independent variables
- Biopsy reports with inconclusive histopathologic diagnosis
- Repeated biopsy

2.5 Sample Size and sampling technique

Histopathology hard copy reports of 543 patients at JMC's pathology department from September 12, 2018, to September 12, 2020, were retrieved from the pathology department's data archive, and those reports that fulfilled the inclusion criteria were manually selected and then grouped by year after being retrieved from the entire hard copy of 3766 histopathology reports in the department's archives. Relevant information was collected via a checklist from 469 hard copies. See Figure 1 below.

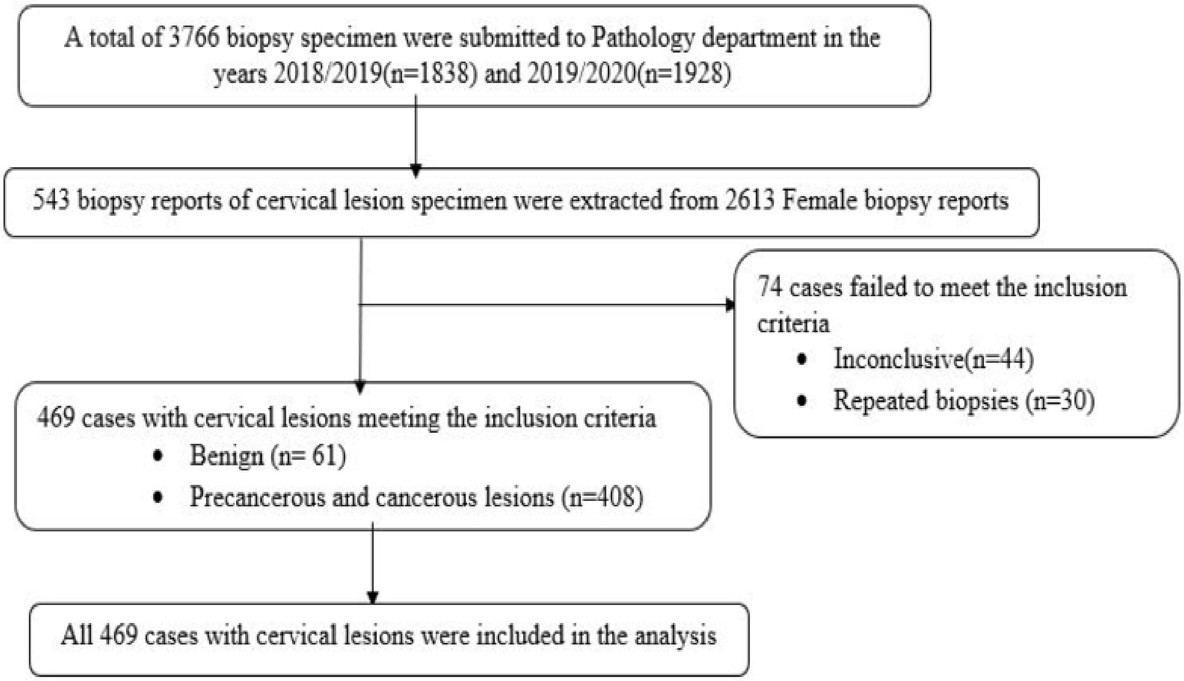

Figure 1: Diagram showing the sampling procedure of the selected 469 cervical lesion biopsy records from 2018 to 2020

2.6. Study variables

2.6.1. Dependent variable

- ❖ Histopathologic pattern of Cervical tissue specimens

2.6.2. Independent variable

- ♠ Age
- ♠ Place of Residence
- ♠ Type of biopsy
- ♠ Clinical features

2.7 Data collection procedures

Histopathology reports of biopsies submitted from cervical lesions that were routinely processed and from which paraffin sections were taken and stained with hematoxylin and eosin (H&E) for microscopic examination and histopathological diagnosis at JMC, pathology department, from September 12, 2018, to September 12, 2020, were retrieved from the pathology department data archive. Eligible 469 reports fulfilling inclusion and exclusion criteria were extracted and recorded into a prepared checklist containing study variables. All necessary preventive methods for coronavirus disease-19 (COVID-19) were used throughout as per the national protocol by using personal protective equipment, physical distancing, and disinfectants.

2.8 Data Processing and Analysis

Data were cleaned, coded, entered into EpiData v3.1, and exported to SPSS version 26 for analysis. Cross tabulation, the chi-square test, and logistic regression with multivariate analysis were performed to look for associations between the study variables. Variables with P values less than 0.5 were chosen for multivariable logistic regressions. A p-value of 0.05 was used as a cutoff point for identifying predictors for histopathologic patterns. The findings were presented using text, tables, and charts.

2.9 Data quality management

A checklist was adopted after reviewing different kinds of literature and books, and the checklist was pretested on 47 cases (10% of the total sample size) of hard-copy biopsy reports done in the year 2017 that were not included in the current study. Then the checklist was revised with some modifications to the variable, and the final revised checklist was used for data collection. Two days of training were given to the data collectors on how to locate, retrieve, categorize, and record the data, and initial data collection was accompanied by the principal investigator. The principal investigator subsequently followed and supervised while the data collectors were retrieving and recording the biopsy results from the pathology department data archive using checklists.

Following a thorough review of the checklist, data were entered into Epidata on a password-protected computer and exported to SPSS version 26 for analysis.

2.10 Ethical consideration

Before data collection, the proposal for this study was submitted to Jimma University's research and ethical committee. Then, ethical clearance for the use of secondary data and a waiver of informed consent was obtained from the Institutional Review Board (IRB) of JMC and submitted to the responsible authorities of JMC. A formal letter of cooperation was also written to obtain permission from the head of the pathology department before using the data. All the information collected from the study was handled confidentially by omitting the personal identification of the pathology reports, and data utilization was carried out in accordance with the ethical and scientific standards outlined in national and international guidelines.

2.11 Operational Definitions (1,6)

Cervical tissue specimens: specimens of patients with cervical complaints such as punch biopsies and hysterectomy specimens.

Cancerous cervical lesion: malignant lesions of the cervix.

Histopathologic pattern: a specific type of diagnosis made on biopsy specimens.

Noncancerous cervical lesion: benign conditions of the cervix.

Precancerous cervical lesion: precursor lesions of cervical cancer (LSIL and HSIL)

2.12 Limitations of the Study

Biopsy results are not computerized and compiled with the clinical data of patients; thus, most of the factors associated with cervical cancer cannot be assessed. Confirmatory immunohistochemistry and HPV DNA testing could not be included in this study because of the limited setup. Power analysis for sample size calculation was not performed.

3. Results and discussions

3.1 Results

In 2018–2019 (n = 1838) and 2019–2020 (n = 1928), a total of 3766 biopsy specimens (2613 female and 1153 male) were submitted to the pathology department. From 2613 female biopsy reports, 543 biopsy reports of cervical lesion specimens were extracted, of which 74 were excluded because they fulfilled the exclusion criteria. Cervical biopsy specimens accounted for 14.4% of the total biopsy specimens and 20.8% of the total biopsy specimens from female patients who submitted biopsy specimens during the study period.

3.1.1. Sociodemographic profiles

Of the 469 cervical lesion cases, 244 cases (52%) were from 2018–2019 and 225 (48%) cases were from 2019–2020 (Figure 2)

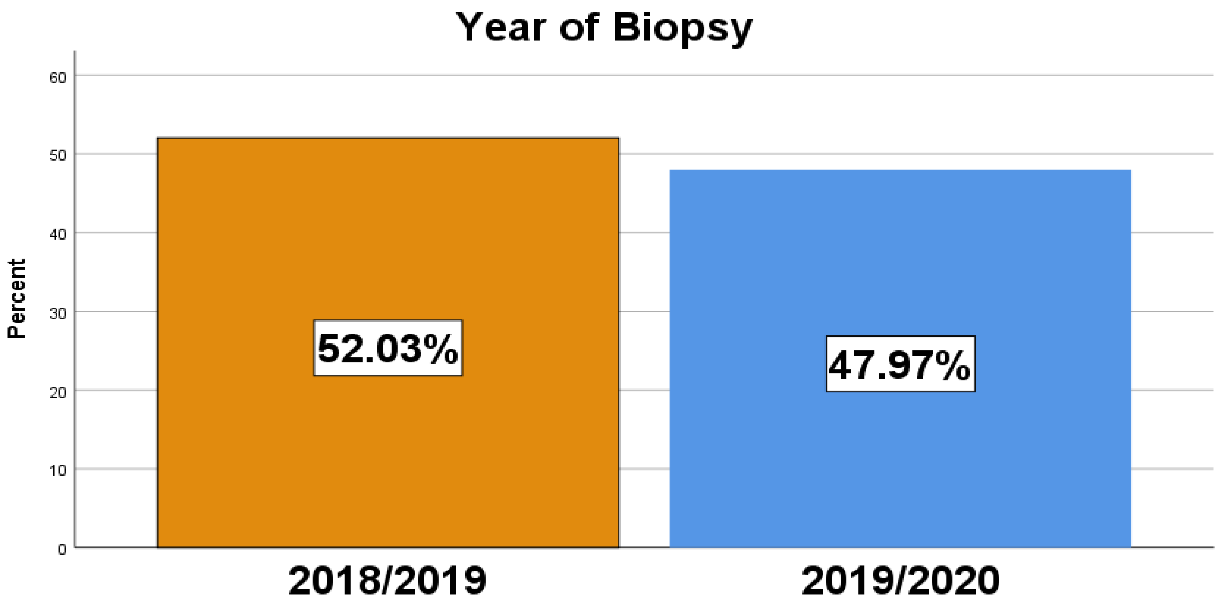

Figure 2: Pie chart showing the distribution of cervical lesions between 2018 and 2020

The age distributions have a minimum value of 22 years and a maximum value of 85 years, with a mean age of 47.06 years and a standard deviation of 11.019 years. The maximum age distribution of cervical lesions was in the 41–50-year age range, accounting for 154 (32.8%) biopsies, followed by 31–40 years with 143 (30.5%) biopsies, and 51–60 years with 102 (21.7%) biopsies.

More than half of precancerous and cancerous cervical lesions (65.46%) occurred after the age of 40 years, while benign lesions accounted for 47.5% after the age of 40 years. This was statistically significant. (X^2=23.54, DF =5 *p*=**0.001**).

As the patient's age increases, the risk of precancerous and cancerous cervical lesions increases, and the third and fourth decades of life were found to be associated with an increased risk of having precancerous and cancerous cervical lesions (Figure 3).

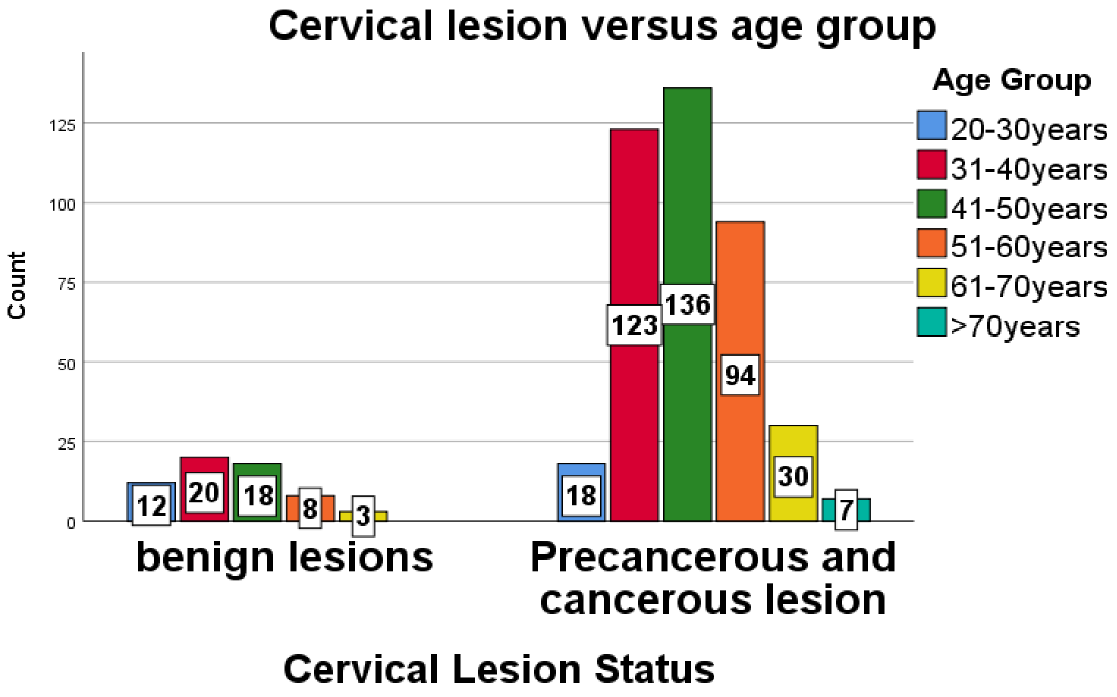

Figure 3: Bar graph showing cervical lesion status with age groups

Most of the patients (77.8%) were from surrounding areas with variable distances from Jimma town, while 104 (22.2%) were from Jimma town. The majority of patients coming from the periphery were from the Jimma zone, accounting for 58.4% (Figure 4). Most (88.8%) of the cases from the surrounding areas of Jimma were precancerous and cancerous cervical lesions, while 80.8% of the cases in Jimma town were precancerous and cancerous cervical lesions. This showed a strong association between precancerous and cancerous cervical lesions and residency. (X2 = 4.67, DF = 1, p = 0.027).

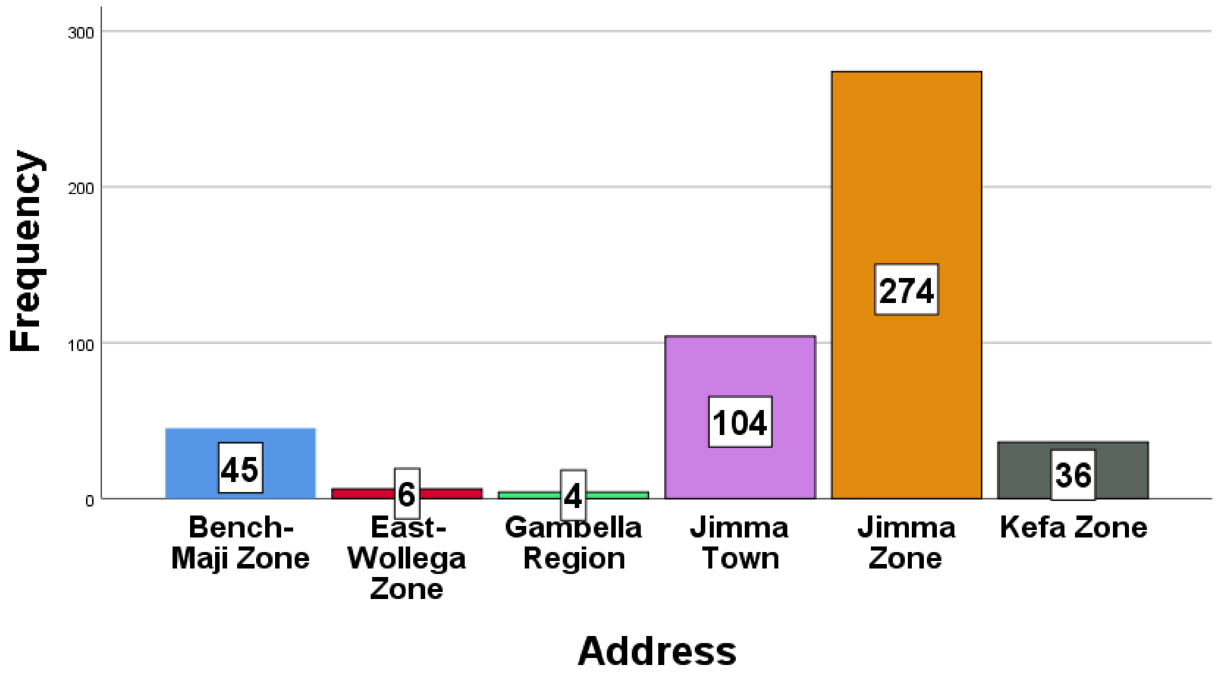

Figure 4: Bar graph showing the distribution of cervical lesions by address

3.1.2. Clinical data of patients with cervical lesions

The most common presenting symptom of patients with cervical lesions was vaginal bleeding alone, accounting for 206 (43.9%) patients, followed by vaginal bleeding with vaginal discharge (108 (23%) patients) and vaginal bleeding with lower abdominal pain (89 (19%) patients). (Table 1). The duration of symptoms in patients with cervical lesions ranged from four days up to sixteen years (Figure 5). The most common type of biopsy performed for patients with cervical lesions was a punch biopsy, accounting for 91.3% followed by abdominal hysterectomy, accounting for 6.4% (Table 2). Most (90%) patients with cervical complaints were clinically diagnosed with cervical cancer (Figure 6)

Table 1: Clinical features/presentation of patients with cervical lesions

Clinical features/presentation	Frequency	Percent
Vaginal Bleeding	226	48.2
Vaginal Bleeding and Vaginal Discharge	108	23.0
Vaginal Bleeding and Lower abdominal pain	89	19.0
Vaginal Discharge	44	9.4
Cervical CA Screening	2	0.4
Total	469	100.0

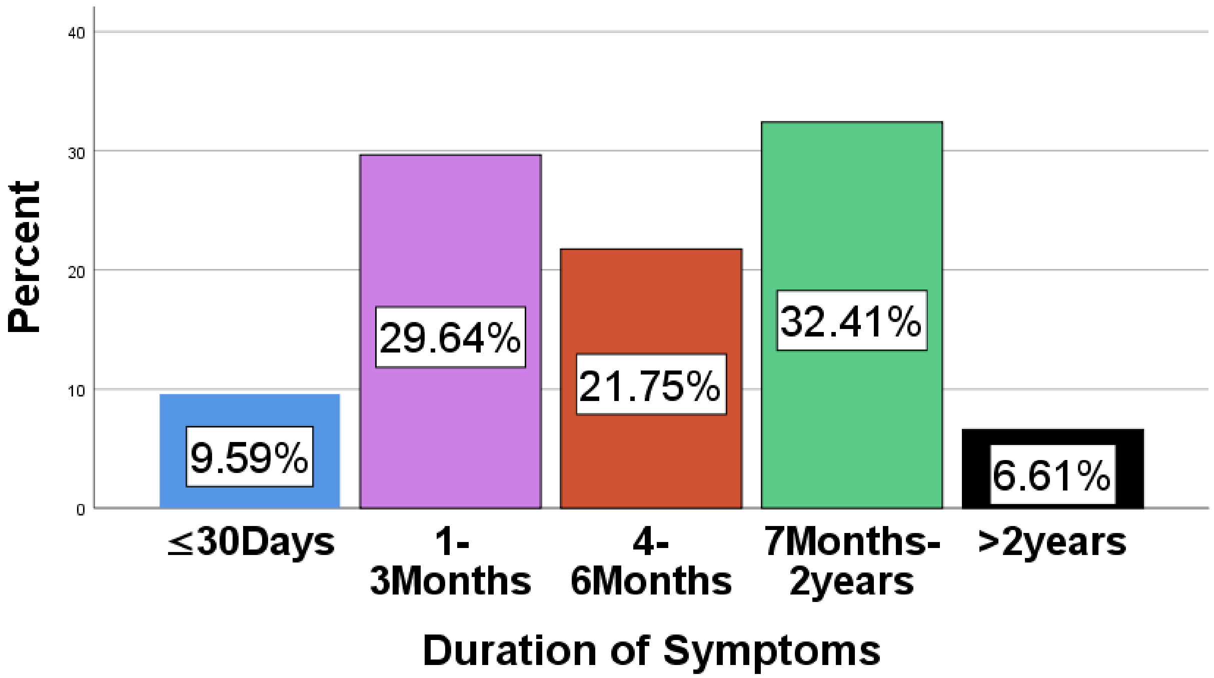

Figure 5: Bar graph showing the distribution of the duration of symptoms in patients with cervical lesions

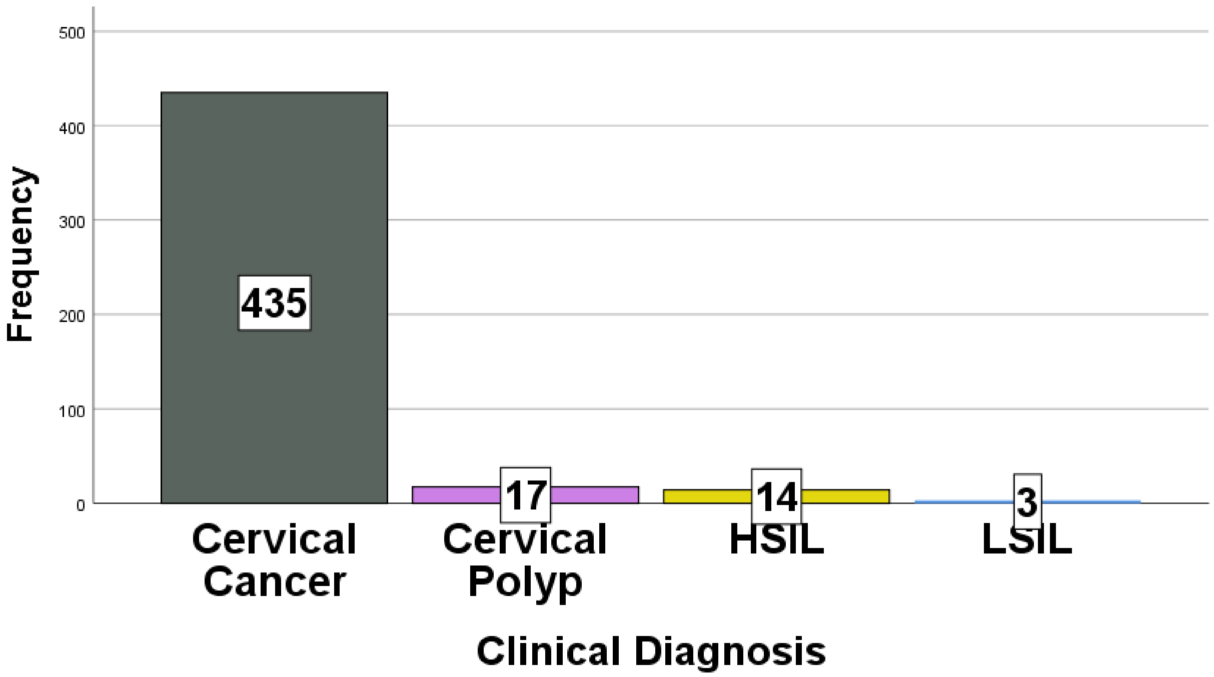

Figure 6: Bar graph showing the distribution of clinical diagnosis of patients with cervical lesions

Table 2: Type of biopsy of patients with cervical lesions

Type of Biopsy/Nature of specimen	Frequency	Percent
Cone Biopsy	3	0.6
Hysterectomy	30	6.4
LEEP	8	1.7
Punch Biopsy	428	91.3
Total	469	100.0

3.1.3. Histopathologic patterns of cervical lesions

Most (87%) of the specimens were precancerous and cancerous cervical lesions, while 13% were benign cervical lesions (Figure 7). The majority (71%) of cases were cancerous cervical lesions, followed by precancerous cervical lesions (16.4%) (Table 3). Squamous cell carcinoma (SCC) was the most frequent cervical cancer diagnosed during the study period, accounting for 96.4% (Table 4). HSIL was the most frequently diagnosed precancerous lesion, accounting for 68.4%. Endocervical polyps were the most commonly diagnosed benign lesions, accounting for 59.3% (Table 3). The most common histologic subtype of SCC was keratinizing SCC, accounting for 77.4% of all cases, while the remaining 22.6% were nonkeratinizing SCC (Figure 8).

Table 3: Numbers and percentages of cervical lesions

**Type of Cervical Lesion**	**Frequency**	**Causes**	**Percentage**
Precancerous cervical lesion	79	54(68.4%) HSIL<br>25(31.6%) LSIL	16.8%
Cancerous cervical lesion	331	See Table 4	70.6%
Noncancerous cervical lesion	59	35(59.3%) Endocervical polyp<br>18(30.5%) Squamous metaplasia<br>6(10.2%) Cervicitis	12.6%
Total	469		100%

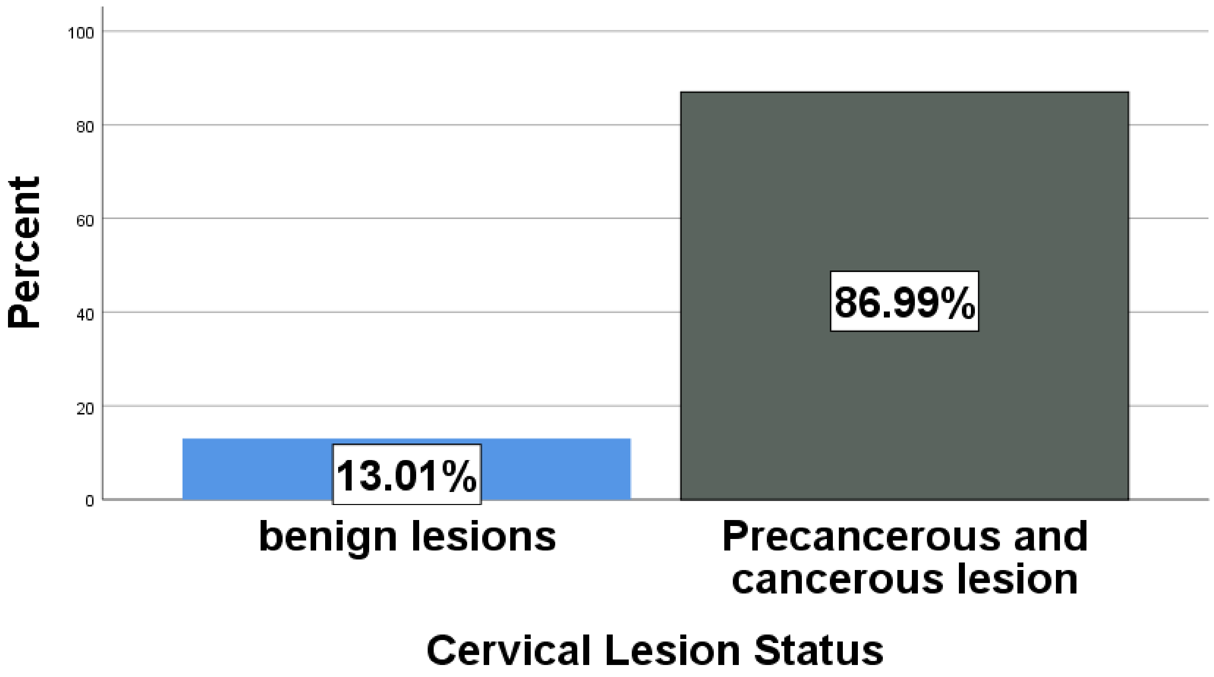

Figure 7: Bar graph showing the distribution of cervical lesion status

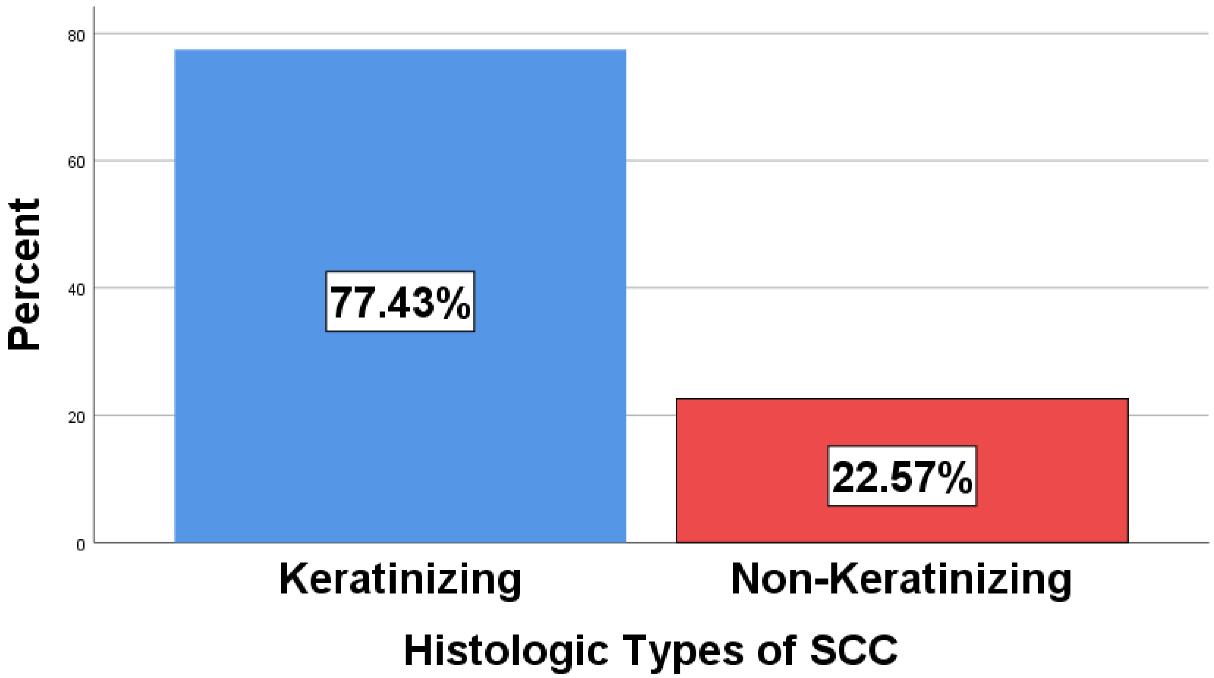

Figure 8: Bar graph showing histologic types of SCC

Table 4: Histopathological classification of cervical cancer

Histopathologic type	Frequency	Percentage
Squamous cell carcinoma	319	96.4%
Adenocarcinoma	11	3.3%
Adenosquamous carcinoma	1	0.3%
Total	331	100%

Cancerous cervical lesions tend to develop in older age groups, and benign conditions tend to develop in younger age groups. SCC occurred most frequently in the 41–50-year age group (N = 104), followed by the 31-40 year (N = 86) and 51–60-year age groups (N = 85). (Table 5)

Table 5: Pattern of the cervical lesion by age group

Age	Histopathologic Diagnosis								
Group	Cervicitis	Squamous Metaplasia	Endocervical Polyp	LSIL	HSIL	Squamous Cell Carcinoma	Adenocarcinoma	Adenosquamous Carcinoma	Total
20-30years	0	4	8	3	1	14	0	0	30
31-40years	2	6	13	11	23	86	1	1	143
41-50years	3	3	10	10	19	104	5	0	154
51-60years	0	3	4	1	6	85	3	0	102
>60years	1	2	0	0	5	30	2	0	40
Total	6	18	35	25	54	319	11	1	469

3.1.4. Factors associated with cervical lesions

Age (*p*=**0.001, COR=1.048**) and place of residence (*p*= **0.032, COR=0.531**) were tested at a P value less than 0.05 in bivariate logistic regression and selected as candidate variables for multivariate logistic regression. Cervical lesions were grouped into benign and HPV-related precancerous and cancerous lesions. Addresses were categorized into Jimma town, which is urban and surrounding areas, which were rural. Multivariate logistic regression analysis was performed on these variables using the step-wise method and the overall model was significant at a P value of 0.001. Age (*p*=**0.001, AOR=1.052**) and place of residency (*p*=**0.015, AOR=0.47**) were shown to be the independent predictors of precancerous and cancerous cervical lesions, hence older women are 1.052 times more likely to develop precancerous and cancerous cervical lesions than younger women and rural dwellers are 0.47 times more likely to develop precancerous and cancerous cervical lesions than urban dwellers which were statistically significant as shown in Tables 6,7 and 8.

Table 6: Bivariate logistic regression of cervical lesion status and age

Independent Variable	B-Coefficient	t-value	P-value	R square	COR
Age	0.154	3.369	0.001	0.024	1.048

COR: -Crude Odds Ratio

Table 7: Bivariate logistic regression of cervical lesion status and address

Independent Variable	Beta Coefficient	t-value	P-value	R square	COR
Address	0.099	2.145	0.032	0.010	0.531

COR: -Crude Odds Ratio

Table 8: Multivariate logistic regression of cervical lesion status and associated factors

Independent Variables	Beta Coefficient	t-value	P-value	AOR	R square
Age	0.162	3.561	0.001	1.052	0.036
Address	0.111	2.435	0.015	0.47	

AOR: - Adjusted Odds Ratio

3.2 Discussion

The most common type of biopsy performed for patients with cervical lesions was a punch biopsy, accounting for 91.3% followed by total abdominal hysterectomy accounting for 6.4%. In a study performed in India, of the total 110 cases, fifty-one were hysterectomy specimens including 25 Wertheim's hysterectomies, 16 total abdominal hysterectomies, and 10 vaginal hysterectomies. Fifty-nine specimens were cervical biopsies(19). Additionally, in a study done in Italy, charts of 224 "low-risk" patients were retrieved and, 50 patients undergoing radical hysterectomy were matched with 100 patients undergoing open radical hysterectomy(20). Similarly, in a study done in Italy on precancerous cervical lesions, 2966 patients underwent conization for CIN2-3 during the study period(21). These and other studies performed in the best centers show a higher number of hysterectomies and conizations because of the well-established chemotherapy set-up for advanced cancers and early screening and the detection of precursor lesions. In the study area, there is a high number of punch biopsies than hysterectomies, because patients will be referred to black lion hospital, Addis Ababa, Ethiopia for further management once the diagnosis of cervical cancer is made by punch biopsies.

Cervical lesions tend to affect females across a wide age range. The age distributions have a minimum value of 22 years and a maximum value of 85 years, with a mean age of 47.06 years and a standard deviation of 11.019 years. This is consistent with Ameya G.'s study on 513 biopsies in Hawassa, Ethiopia, in which the age ranges were 17 to 85 years, with a mean and standard deviation of 42 and 11 years respectively (22). Our study is also similar to a study done on 306 cervical cancer cases in Nigeria, which showed patient ages diagnosed with cervical cancer to range from 18 to 85 years, with peak occurrence in the fifth decade (23). A study done on 1047 specimens in Lagos, Nigeria, also showed the age range of patients as 19–87 years, with a mean age of 49±13 (24). Another study done at TASH, A.A., Ethiopia on women with histologically verified cancer of the cervix uteri also showed that the mean age was 49 years (21–91 years) (25). Our findings are consistent with this and other similar studies.

Cervical lesions range from benign inflammatory conditions and polyps to malignant conditions. Of the 469 biopsies submitted to JMC, the department of pathology, most (87%) of the specimens were precancerous and cancerous cervical lesions, while 13% were benign cervical lesions. The majority (70.6%) of cases were cancerous cervical lesions, followed by precancerous cervical lesions (16.8%) and noncancerous cervical lesions (12.6%). A study performed in Malawi on 212 cervical lesions showed that 17% had precancerous lesions, 65% were cancerous, and 18% had both precancerous and cervical cancer, making a total of 83% cancerous lesions (26). Another study performed in India on 110 cases showed that a diagnosis of intraepithelial neoplasia was made in 32.7% of cases, and invasive carcinoma was found in 67.3% of cases (27). Therefore, the finding of our study has a slightly higher number of cancerous cases than this last finding and other similar studies. This is possibly because of the limited resources and services within the study area.

Squamous cell carcinoma (SCC) was the most frequent cervical cancer diagnosed during the study period, accounting for 96.4% of the 333 cancerous cases, followed by adenocarcinoma (3.3%) and adenosquamous carcinoma (0.3%). This is consistent with a study done at JUSH, Jimma, Ethiopia, on 154 cervical cancer cases, which showed that 91% of the cases were SCC, 5.84% were small cell carcinomas, 2.59% were adenocarcinomas, 0.64% were adenosquamous carcinomas (28). Our study is also consistent with a study performed in Cameron on 2078 cervical cancer cases, which showed that the most common cervical cancer was SCC (81.18%), followed by adenocarcinoma (12.95%) (29). In another study done at TASH, Addis Ababa, Ethiopia, cancer of the uterine cervix was found to be the most common malignancy among all gynecological malignancies (30). Another study done at A.A., Ethiopia on women with postmenopausal bleeding also showed that about 94% of the cervical malignancies were squamous cell carcinoma(31). A study done at Saint Paul Hospital Millennium Medical College (SPHMMC), Addis Ababa, Ethiopia, on breast and gynecologic malignancies also showed that SCC of the cervix was the most common type of cervical malignancy detected, accounting for 90% of all cases, while adenocarcinoma (3.85%) was the second most observed type of cervical cancer (32). Similarly, according to a study done in Bangladesh on gynecologic malignancy, SCC was the most common histopathologic type in cervical and vulvar cancers (33). Another study done in Sudan on 372 patients showed that SCC (NOS) was the most common subtype (76.6%) and adenocarcinoma was the second most common subtype (12.6%) (34).

On the contrary, according to a study performed in India over a period of 2 years, the most frequent condition diagnosed on cervical biopsies and hysterectomy specimens was benign cervical polyp (66.3%), followed by carcinoma (23.7%) and squamous intraepithelial lesions (0.2%)(35). These last studies and other studies performed in Western countries show a higher number of benign cervical lesions because of early screening and the detection of precursor lesions. Although cervical cancer can be prevented by vaccination and screening and cured if identified early, this is not yet fully implemented in the study area.

Of the 79 precancerous cervical conditions diagnosed during the study period, HSIL was the most frequently diagnosed precancerous lesion, accounting for 68.4%, with the other 31.6% being LSIL. This is consistent with a study performed in Hawassa, Ethiopia, on 513 biopsies, which showed that among precancerous cervical lesions, HSIL was the predominant lesion (39.8%) (22). Similarly, a study performed in India showed that cervical intraepithelial neoplasia (CIN) 1 was diagnosed in 36.1% of patients, followed by CIN 2 (33.3%) and CIN 3 (30.6%) (27).

Of the 59 benign cases, endocervical polyps were the most commonly diagnosed benign lesion, accounting for 59.3%, followed by squamous metaplasia (30.5%) and cervicitis (10.2%). A study performed in Nigeria on 176 cervical specimens showed that inflammatory lesions accounted for 55 cases (59.8%) of non-neoplastic cervical lesions, followed by endocervical polyps, which accounted for 15 cases (16.3%) of non-neoplastic cervical lesions. Among the inflammatory lesions, chronic nonspecific cervicitis was the most commonly encountered lesion, constituting 40 cases (72.2%) of all inflammation (36). In a study performed at TASH, Addis Ababa, Ethiopia chronic cervicitis was the commonest benign lesion (16.8%) (37). Contrary to this and other similar studies, our study has the lowest number of cervicitis cases, possibly because the majority of cervicitis cases are managed clinically without histopathological confirmation once a clinical diagnosis is made as per the National STD Guidelines.

4. Conclusion and recommendation

4.1. Conclusion

Cervical diseases include a wide range of disorders and occur across different age groups. The age distributions have a minimum value of 22 years and a maximum value of 85 years, with a mean age of 47.06 years. The most common presenting symptom in patients with cervical lesions was vaginal bleeding. The most common type of biopsy performed for patients with cervical lesions is a punch biopsy. Most (90%) patients with cervical complaints were clinically diagnosed with cervical cancer. Cervical lesions range from benign inflammatory conditions and polyps to malignant conditions. Most cervical lesions were precancerous and cancerous. Squamous cell carcinoma (SCC) was the most frequent type of cervical cancer. HSIL was the most frequently diagnosed precancerous cervical lesion. Endocervical polyps were the most commonly diagnosed benign cervical lesion. Precancerous and cancerous cervical lesions tend to increase with increasing age and in those patients who come from peripheral areas.

4.2. Recommendation

There was a rising burden of cervical cancer. This implies that there is a need to educate the community to improve health-seeking behavior and on possible preventive strategies for cervical cancer. It is also strongly recommended that JMC's Department of Pathology commence cervical cytological examinations to detect dysplasia before it turns into full-blown cancer. We recommend that the Federal Ministry of Health strengthen the ongoing nationwide HPV vaccinations, screenings, and interventions for cervical cancer at a much faster rate.

Acknowledgment

I thank Jimma University for allowing me to undertake the research. I extend my gratitude to Jimma Medical Center, Department of Pathology, for providing me with the necessary resources. I also thank my advisors and friends for giving me constructive comments and important information to prepare my proposal and thesis. Last but not least, my warm gratitude goes to all my family members for providing me with emotional support during this study.

References

1. John Goldberg LLJMcKenney. “Rosai and Ackerman’s Surgical Pathology” 11th eds. In: 11th ed. Philadelphia: Elsevier, 2018. p. 1260–93.
2. Iqbal B, Masood I, Aiman A, Yasir M, Mushtaq I, Verma V. Study of Metaplastic Lesions of Different Parts of The Female Genital Tract: A Prospective Study. Internet Journal of Pathology. 2010 Jul;
3. Kumar V., Abbas K. AJ. Robbins, and Cotrans Pathologic Basis of Diseases 9th ed. 9th editio. Philadelphia: Elsevier; 2015. 1001–1007 p.
4. Robboy SJ. Robboy’s Pathology of the Female Reproductive Tract [Internet]. Elsevier Health Sciences; 2009 [cited 2023 Jun 6]. 1001–1007 p. Available from: https://books.google.com.et/books?hl=en&lr=&id=ab545XL-MBEC&oi=fnd&pg=PR29&dq=3.+Robboy+S.,+Mutter+G.,Prat+J.,Et+al.+Robboy%27s+Pathology+of+Female+Reproductive+Tract.+2nd+Edition.+Philadelphia+:+Elsevier,+2009,+pp.+189-295.&ots=d4zyLlxZJo&sig=kp7dnNNhqn3zRa944GvDuN5EvYs&redir_esc=y#v=onepage&q&f=false
5. Fletcher CDM. Diagnostic Histopathology of Tumors. In: 4th ed. Philadelphia: Elsevier; 2013 [cited 2023 Jun 6]. p. 814–38. Available from: https://shop.elsevier.com/books/diagnostic-histopathology-of-tumors-2-volume-set/fletcher/978-0-323-42860-6
6. Herrington CS, Board WC of TE. Female Genital Tumours [Internet]. WHO Female Genital Tumours. 2020 [cited 2023 Jun 6]. 632 p. Available from: https://www.research.ed.ac.uk/en/publications/who-classification-of-tumours-female-genital-tumours
7. Ikechebelu JI, Onyiaorah I V., Ugboaja JO, Anyiam DCD, Eleje GU. Clinicopathological analysis of cervical cancer seen in a tertiary health facility in Nnewi, south-east Nigeria. http://dx.doi.org/103109/01443610903531394 [Internet]. 2010 Apr [cited 2023 Jun 6];30(3):299–301. Available from: https://www.tandfonline.com/doi/abs/10.3109/01443610903531394
8. Ethiopian Federal Ministry of Health. FEDERAL MINISTRY OF HEALTH ETHIOPIA DISEASE PREVENTION AND CONTROL DIRECTORATE NATIONAL CANCER CONTROL PLAN [Internet]. 2016 [cited 2023 Jun 6]. Available from: https://www.google.com/url?sa=D&q=https://www.iccp-portal.org/sites/default/files/plans/NCCP%2520Ethiopia%2520Final%2520261015.pdf&ust=1686138720000000&usg=AOvVaw0eDMP3rQOPzm9DhCUtnBPJ&hl=en&source=gmail
9. Ferlay J, Shin HR, Bray F, Forman D, Mathers C, Parkin DM. Estimates of worldwide burden of cancer in 2008: GLOBOCAN 2008. Int J Cancer [Internet]. 2010 Dec 15 [cited 2023 Jun 6];127(12):2893–917. Available from: https://onlinelibrary.wiley.com/doi/full/10.1002/ijc.25516
10. Ferlay J, Colombet M, Soerjomataram I, Mathers C, Parkin DM, Piñeros M, et al. Estimating the global cancer incidence and mortality in 2018: GLOBOCAN sources and methods. Int J Cancer. 2019 Apr 15;144(8):1941–53.
11. Ferlay J, Soerjomataram I, Dikshit R, Eser S, Mathers C, Rebelo M, et al. Cancer incidence and mortality worldwide: Sources, methods and major patterns in GLOBOCAN 2012. Int J Cancer. 2015 Mar 1;136(5): E359–86.

12. Information Centre on HPV and Cancer. Human Papillomavirus and Related Diseases Report WORLD [Internet]. Available from: www.hpvcentre.net
13. Deressa BT, Cihoric N, Badra EV, Tsikkinis A, Rauch D. Breast cancer care in northern Ethiopia – cross-sectional analysis. BMC Cancer [Internet]. 2019 Apr 25 [cited 2023 Jun 6];19(1). Available from: /pmc/articles/PMC6485046/
14. Memirie ST, Habtemariam MK, Asefa M, Deressa BT, Abayneh G, Tsegaye B, et al. Estimates of cancer incidence in Ethiopia in 2015 using population-based registry data. J Glob Oncol. 2018 Mar 1;2018(4):1–11.
15. Ntekim A, Ntekim A. Cervical Cancer in Sub Sahara Africa. Topics on Cervical Cancer with an Advocacy for Prevention [Internet]. 2012 Mar 2 [cited 2023 Jun 6]; Available from: https://www.intechopen.com/chapters/30747
16. Census 2007. Stastical Oromia [Internet]. [cited 2023 Jun 6]. Available from: http://www.csa.gov.et/census-report/complete-report/census-2007?start=5
17. Jimma University. Home – JU Official Website [Internet]. [cited 2023 Jun 6]. Available from: https://ju.edu.et/
18. Mapcarta. Jimma University Map - University - Ethiopia - Mapcarta [Internet]. [cited 2023 Jun 6]. Available from: https://mapcarta.com/33099124
19. Gupta M, Basavaraj P. Histopathological Spectrum of Premalignant and Malignant Lesions of Uterine Cervix. National Journal of Laboratory Medicine [Internet]. 2018; 7:8. Available from: http://files/346/Gupta y Basavaraj - 2018 - Histopathological Spectrum of Premalignant and Mal.pdf
20. Di Donato V, Bogani G, Casarin J, Ghezzi F, Malzoni M, Falcone F, et al. Ten-year outcomes following laparoscopic and open abdominal radical hysterectomy for “low-risk” early-stage cervical cancer: A propensity-score based analysis. Gynecol Oncol. 2023 Jul; 174:49–54.
21. Giannini A, Di Donato V, Sopracordevole F, Ciavattini A, Ghelardi A, Vizza E, et al. Outcomes of High-Grade Cervical Dysplasia with Positive Margins and HPV Persistence after Cervical Conization. Vaccines (Basel). 2023 Mar 18;11(3).
22. Ameya G, Yerakly F. Characteristics of cervical disease among symptomatic women with histopathological sample at Hawassa University referral hospital, Southern Ethiopia. BMC Womens Health [Internet]. 2017 Sep 29 [cited 2023 Jun 6];17(1):1–7. Available from: https://link.springer.com/articles/10.1186/s12905-017-0444-5
23. Silas OA, Yakubu D, Jegede OO, Ajetunmobi OI, Mosugu O. A histopathologic review of cervical cancers in Jos University Teaching Hospital, Jos, Nigeria. Annals of Tropical Pathology [Internet]. 2018 [cited 2023 Jun 6];9(2):135. Available from: https://www.atpjournal.org/article.asp?issn=2251-0060;year=2018;volume=9;issue=2;spage=135;epage=138;aulast=Silas
24. Faduyile F, Soyemi S, Wright K, Osuolale F. Histopathological study of surgical cervical biopsies in Lagos, Nigeria. Trop J Obstet Gynaecol. 2017;34(2):124.
25. Kantelhardt EJ, Moelle U, Begoihn M, Addissie A, Trocchi P, Yonas B, et al. Cervical Cancer in Ethiopia: Survival of 1,059 Patients Who Received Oncologic Therapy. Oncologist [Internet]. 2014 Jul 1 [cited 2023 Jun 6];19(7):727–34. Available from: https://academic.oup.com/oncolo/article/19/7/727/6399365
26. Bangara FF, Marimo C, Kaile T, Simakando M, Tomoka T, Arabi G, et al. Histological Characterization of Invasive Cervical Cancer and Precursor Lesions in Malawian Women

Presenting at Queen Elizabeth Central Hospital (QECH): A Cross-Sectional Study. J Virol Antivir Res. 2017;06(01).
27. Mamta Gupta PB. Histopathological Spectrum of Premalignant and Malignant Lesions of Uterine Cervix. National Journal of Laboratory Medicine [Internet]. 2018 [cited 2023 Jun 6];7(1). Available from: DOI: 10.7860/NJLM/2018/32340:2276
28. Saleem A, Bekele A, Fitzpatrick MB, Mahmoud EA, Lin AW, Velasco HE, et al. Knowledge and awareness of cervical cancer in Southwestern Ethiopia is lacking: A descriptive analysis. PLoS One [Internet]. 2019 Nov 1 [cited 2023 Jun 6];14(11): e0215117. Available from:https://journals.plos.org/plosone/article?id=10.1371/journal.pone.0215117
29. Paul ENJ, Charlotte TN, Jules OML, Godefroy S, Daniel ESJ, Michelle EFA, et al. Epidemiological and Histological Profile of Cervical Cancer in Cameroon: About 2078 Cases. Open J Obstet Gynecol [Internet]. 2016 [cited 2023 Jun 6];06(04):232–9. Available from: http://file.scirp.org/Html/
30. Tigeneh W, Molla A, Abreha A, Assefa M. Pattern of Cancer in Tikur Anbessa Specialized Hospital Oncology Center in Ethiopia from 1998 to 2010. Int J Cancer Res Mol Mech [Internet]. 1998;1. Available from: http://dx.doi.org/10.16966/2381-3318.103
31. Ergete W, Tesfaye A, Health EJ. The Ethiopian Journal of Health Development Histopathological findings of post-Menopausal bleeding in Ethiopian women.
32. Hailu HE, Mondul AM, Rozek LS, Geleta T. Descriptive Epidemiology of breast and gynecological cancers among patients attending Saint Paul’s Hospital Millennium Medical College, Ethiopia. PLoS One [Internet]. 2020 [cited 2023 Jun 6];15(3): e0230625. Available from: https://journals.plos.org/plosone/article?id=10.1371/journal.pone.0230625
33. Rahman MA, Siddika ST, Mazid M. Gynecological Cancers in Surgical Specimens – A Hospital Based Analysis. Med Today. 2015 Jul 16;26(2):78–82.
34. Rayis DAM, Zulfu A, Yassin K, Merghani A. Histopathological pattern of gynecological malignancies: National Health Laboratory (NHL), Sudan. GPH - International Journal of Health Sciences and Nursing [Internet]. 2018 Aug 30;1(1). Available from: http://gphjournal.org/index.php/hsn/article/view/45
35. Atul J. RJ, IE al. Histopathological study of tumors of Cervix at MGM Medical College. Medical College of India. 2014;1(1).
36. Forae G, Nwachokor F. Morphological spectrum of non-neoplastic lesions of the uterine cervix in Warri, South-South, Nigeria. Niger J Clin Pract. 2013;16(4):429.
37. Nigatu B, Gebrehiwot Y, Kiros K, Ergete W. Number 1 Ethiopian Journal of Reproductive Health.

Click here to access/download
Supporting Information
Biopsy form.pdf

Click here to access/download
Supporting Information
Ethical Approval.PDF

Click here to access/download
Supporting Information
Questionner checklist.pdf

Click here to access/download
Supporting Information
STROBE-checklist-v4-combined-PlosMedicine.docx

SPSS Data

Click here to access/download
Supporting Information
Cervix Data for Analysis.sav

Click here to access/download
Supporting Information - Compressed/ZIP File Archive
Cervic Figure Collection.zip

Histopathologic patterns and factors associated with cervical lesions at Jimma Medical Center, Jimma, Southwest Ethiopia: a two-year cross-sectional study

Birhanu Hailu Tirkaso1*,
Tesfaye Hurgesa Bayisa2, and
Tewodros Wubshet Desta3

1Department of Biomedical Sciences, Mizan-Tepi University, Mizan, Southwestern Region, Ethiopia

2Department of Pathology, Jimma University, Jimma, Oromia, Ethiopia

3Department of Pathology, Dire Dawa University, Dire Dawa, Ethiopia

**Corresponding e-mail: birhanuhailu@mtu.edu.et*

Abstract

Background: The cervix is the lower portion of the uterus, which connects this organ to the vagina through the endocervical canal.

Objective: This study aimed to determine the histopathologic patterns and factors associated with cervical lesions at Jimma Medical Center from September 12, 2018, to September 12, 2020.

Methods: A 2-year facility-based cross-sectional study was conducted from May 1 to June 30, 2019.

Result: In this study, cervical cancer was the most common (71%) cause of cervical lesions. Squamous cell carcinoma was the most frequent cervical cancer diagnosed during the study, accounting for 96.4% of 331 cancerous cases, followed by adenocarcinoma (3.3%). High-grade squamous intraepithelial lesions were the most frequently diagnosed precancerous lesions, accounting for 68.4% of cases. Endocervical polyps were the most commonly diagnosed benign lesions, accounting for 59.3% of cases.

Conclusion: The maximum age distribution of cervical lesions was in the 41–50-year age range. Squamous cell carcinoma was the most frequent type of cervical cancer. High-grade squamous intraepithelial lesions were the most frequently diagnosed precancerous cervical lesions. The most common benign cervical lesions were endocervical polyps.

Recommendation: We recommend educating the community to improve health-seeking behavior and on possible preventive strategies for cervical cancer.

Keywords: Histopathology, Cervical Lesions, Cervical Cancer, Jimma Medical Center, Ethiopia.

1. Introduction

1.1. Background

The endocervical canal connects the uterus to the vagina through the cervix, which is the bottom part of the uterus. Non-keratinizing squamous epithelium covers the majority of the exocervix. A layer of columnar mucus-secreting cells makes up the glandular mucosa of the endocervix, and the intersection between the squamous and glandular epithelia is called the squamocolumnar junction(1,2). The squamocolumnar junction undergoes eversion with the onset of puberty, causing columnar epithelium to become visible on the exocervix. The exposed columnar cells eventually undergo squamous metaplasia, forming a region called the transformation zone(3). The transformation zone is the area where the expected transformation to the metaplastic squamous epithelium and the abnormal transformation to CIN usually occur(4).

The range of neoplastic conditions of the cervix arises at or near the cervical transformation zone, ranging from precancerous intraepithelial neoplasia to epithelial malignancies, mixed epithelial and mesenchymal tumors, mesenchymal tumors, melanocytic tumors, and miscellaneous rare malignancies(5).

The epidemiology of precancerous lesions and squamous cell carcinoma (SCC) of the cervix follows the epidemiology of HPV infections. In developed countries, adenocarcinoma now makes up 10–25% of all cervical carcinomas, up from 5–10% thirty years ago. This rise is due to a decrease in squamous cell carcinomas brought on by screening programs and improved glandular lesions detection in cervical cytology samples. It is advised to employ a two-tier system of low- and high-grade intraepithelial lesions rather than the three-tier CIN 1, CIN 2, and CIN 3 terminology since it is more biologically applicable and histologically repeatable. The pairing of CIN 2 and CIN 3 as an HSIL is supported by our enhanced understanding of HPV biology. The typical type of SCC can be either keratinizing or nonkeratinizing (6).

According to a 5-year retrospective study done on 75 cases in Nigeria, cervical cancer has an incidence of 65.2% of all gynecological cancers and 13.4% of all gynecological admissions. The

modal age was in the 60-70 years range. SCC of varying differentiation was the most common histological type, accounting for 89.3 %, and adenocarcinoma accounted for only 8.0%. The modes of presentation were post-menopausal bleeding (84.0%), vaginal discharge (72.0%), contact bleeding (63.9%), and abdominal pain (56.2%)(7).

1.2. Problem statement

The overall burden of cancer is thought to be increasing globally, especially in developing nations. Thirteen million people will pass away from cancer and twenty-one million will receive a diagnosis by the year 2030. Even while mortality rates are just 8% to 15% higher in more industrialized nations, incidence rates for all malignancies combined are more than twice as high in more developed nations compared to less developed ones. This mismatch mostly reflects variations in diagnostic and therapeutic modalities' accessibility and availability. Similar to how tumors are more frequently found in the advanced stages in less developed nations, which contributes to the discrepancy in fatality rates compared to incidence. The National Cancer Control Plan (NCCP) of Ethiopia has established challenging goals to advance preventative intervention techniques, introduce screening exams for early detection, and provide chemotherapy, surgery, and palliative care in diagnosis and treatment. As seen in industrialized countries, it takes years for these measures to significantly reduce the incidence of cervical cancer (8).

Cervical cancer is one of the most common cancers in women, with approximately 0.5 million cases worldwide in 2008. With an average annual growth of 0.6%, cervical cancer cases grew from an estimated 378,000 in 1980 to 500,000 per year in recent years. Roughly 76% of recent cases occur in low-resource countries, with numbers rising everywhere but in high-income nations. In countries with greater affluence, the median age at death is 55. Effective programs to detect cancer precursors along with infrastructure to clinically manage precursor lesions are largely responsible for the significant differences in incidence and mortality between the more severely affected nations in Africa and Asia and the lower rates in wealthier North American and European countries (6) (9).

Cancer is one of the major non-communicable diseases (NCDs). Together with NCDs, cancer causes over 60% of total global mortality every year. Although communicable diseases still remain the leading killers, the incidence and mortality from NCDs are rising rapidly in many developing

countries. This has resulted in a double disease burden, which is putting strain on the existing health system (8) (10).

According to GLOBOCAN 2018, the global cancer burden has risen to 18.1 million cases and 9.6 million cancer deaths. One in six women and one in five men worldwide develop cancer during their lifetime, and one in eleven women and one in eight men die from the disease. The incidence of cancer worldwide is expected to rise to 26.4 million, with 17 million deaths by 2030 (11,12).

A 2-year retrospective study done on 3231 samples (1263 biopsies and 1968 FNAC) at UOG: North-West Ethiopia showed that 540 (16.7%) were malignant cancer cases. Cervical cancer and breast cancer were the two most common cancer types in the hospital, with cervical cancer ranking first (13).

In 2015, an estimated 21,563 and 42,722 incident cancer cases were diagnosed in males and females, respectively, according to a study based on primary data on 8539 patients from Addis Abeba's population-based cancer registry and supplemented by 1,648 cancer case data from six regions. Cervical cancer was the second most common and prevalent cancer, making up 23% of cancer cases, preceded by breast cancer (33%) (14).

Cervical cancer accounts for 22.5% of all cancer cases in women in sub-Saharan Africa, and the majority of women who develop cervical cancer live in rural areas. WHO estimated that cervical cancer will kill more than 443,000 women per year worldwide by 2030, nearly 90% of them in sub-Saharan Africa (15).

1.3. Rationale of the Study

In Ethiopia, cervical cancer is the second most common female cancer. Throughout the world, HPV infections are the most common cause of cervical cancer. Out of more than 100 different HPV types, infection with HPV 16 and 18 has been associated with more than 70% of cervical cancers. Cervical cancer is a preventable and curable disease. It can be prevented by vaccination and screening and cured if identified early.

Despite the fact that high-risk HPV infection is a significant cause, only a small percentage of HPV-infected women develop invasive cancer, implying the presence of HPV infection co-factors that lead to cervical cancer development. According to studies carried out in different parts of Ethiopia, including the study area, knowledge about cervical cancer is very low. Although the NCCP launched nationwide HPV vaccinations in the 2016–2019 national cancer control plan, the effects take years to be seen in primary and secondary school students. Furthermore, PAP smears are only performed in a few centers across the country, and other health institutions in the country's outskirts perform a visual inspection with acetic acid (VIA). To my knowledge, no studies have included benign cervical conditions in the study area. The clinicopathologic studies done in the study area were conducted ten years ago.

1.4. Significance of the Study

This study augments the clinicopathologic studies done in southwestern Ethiopia from a histopathologic point of view, including benign cervical conditions. In addition, this study will serve as a baseline for future studies to be carried out at the national or continental level during the post-HPV vaccination era. The result of this study also serves as input for JMC, Zonal and Regional Health Bureaus, and the Ethiopian Federal Ministry of Health (EFMOH) in evaluating and monitoring the screening and preventive strategies.

2 Materials and methods

2.1 Study area

The study was conducted at Jimma Medical Center, formerly called Jimma University Specialized Hospital (JUSH). JMC was established in 1938 in Jimma Town, located 352 kilometers southwest of Addis Ababa, the capital of Ethiopia. Jimma is the main town in southwestern Ethiopia with an estimated population of 120,960. It has a latitude of 7° 41′ 6′′ North, a longitude of 36° 49′ 53′′ East, and an elevation of 1738 meters (5702 feet). Currently, the hospital is a teaching and referral hospital in the southwestern part of the country, providing service for approximately 15,000 inpatients, 160,000 outpatient attendants, 11,000 emergency cases, and 4,500 deliveries in a year, coming from the catchment population of about 15 million (16–18).

The pathology department is one of the most important departments at JMC, providing services such as histopathology diagnostics, FNAC, fluid cytology, and hematopathology, with an annual average flow of 1,928 histopathology biopsy samples, over 5,000 FNAC, and over 200 BMA.

2.2 Study Design and Period

A 2-year facility-based cross-sectional study was conducted from May 1 to June 30, 2019.

2.3 Population

2.3.1 Target population

All female patients in the catchment area of Jimma Medical Center.

2.3.2 Source population

All female patients who submitted biopsy specimens to the pathology department for histopathology diagnosis from September 12, 2018, to September 12, 2020.

2.3.3 Study population

Histopathology reports of all female patients who submitted cervical tissue specimens to the pathology department from September 12, 2018, to September 12, 2020.

2.3.4 Study Unit

Histopathology reports of selected female patients who submitted cervical tissue specimens to the pathology department from September 12, 2018, to September 12, 2020, and fulfilled the inclusion criteria.

2.4 Inclusion and exclusion criteria

2.4.1 Inclusion Criteria

All female patients who submitted cervical tissue specimens to the pathology department from September 12, 2018, to September 12, 2020.

2.4.2 Exclusion Criteria

- Biopsy reports missing two or more independent variables
- Biopsy reports with inconclusive histopathologic diagnosis
- Repeated biopsy

2.5 Sample Size and sampling technique

Histopathology hard copy reports of 543 patients at JMC's pathology department from September 12, 2018, to September 12, 2020, were retrieved from the pathology department's data archive, and those reports that fulfilled the inclusion criteria were manually selected and then grouped by year after being retrieved from the entire hard copy of 3766 histopathology reports in the department's archives. Relevant information was collected via a checklist from 469 hard copies. See Figure 1 below.

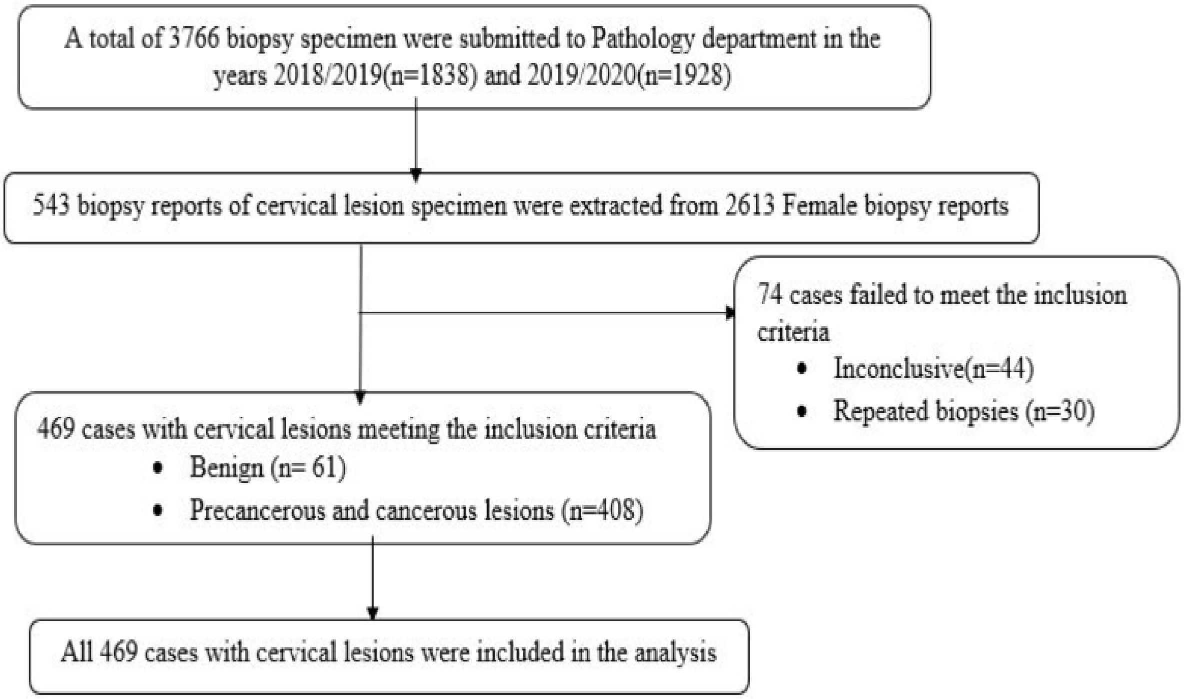

Figure 1: Diagram showing the sampling procedure of the selected 469 cervical lesion biopsy records from 2018 to 2020

2.6. Study variables

2.6.1. Dependent variable

- Histopathologic pattern of Cervical tissue specimens

2.6.2. Independent variable

- Age
- Place of Residence
- Type of biopsy
- Clinical features

2.7 Data collection procedures

Histopathology reports of biopsies submitted from cervical lesions that were routinely processed and from which paraffin sections were taken and stained with hematoxylin and eosin (H&E) for microscopic examination and histopathological diagnosis at JMC, pathology department, from September 12, 2018, to September 12, 2020, were retrieved from the pathology department data archive. Eligible 469 reports fulfilling inclusion and exclusion criteria were extracted and recorded into a prepared checklist containing study variables. All necessary preventive methods for coronavirus disease-19 (COVID-19) were used throughout as per the national protocol by using personal protective equipment, physical distancing, and disinfectants.

2.8 Data Processing and Analysis

Data were cleaned, coded, entered into EpiData v3.1, and exported to SPSS version 26 for analysis. Cross tabulation, the chi-square test, and logistic regression with multivariate analysis were performed to look for associations between the study variables. Variables with P values less than 0.5 were chosen for multivariable logistic regressions. A p-value of 0.05 was used as a cutoff point for identifying predictors for histopathologic patterns. The findings were presented using text, tables, and charts.

2.9 Data quality management

A checklist was adopted after reviewing different kinds of literature and books, and the checklist was pretested on 47 cases (10% of the total sample size) of hard-copy biopsy reports done in the year 2017 that were not included in the current study. Then the checklist was revised with some modifications to the variable, and the final revised checklist was used for data collection. Two days of training were given to the data collectors on how to locate, retrieve, categorize, and record the data, and initial data collection was accompanied by the principal investigator. The principal investigator subsequently followed and supervised while the data collectors were retrieving and recording the biopsy results from the pathology department data archive using checklists.

Following a thorough review of the checklist, data were entered into Epidata on a password-protected computer and exported to SPSS version 26 for analysis.

2.10 Ethical consideration

Before data collection, the proposal for this study was submitted to Jimma University's research and ethical committee. Then, ethical clearance for the use of secondary data and a waiver of informed consent was obtained from the Institutional Review Board (IRB) of JMC and submitted to the responsible authorities of JMC. A formal letter of cooperation was also written to obtain permission from the head of the pathology department before using the data. All the information collected from the study was handled confidentially by omitting the personal identification of the pathology reports, and data utilization was carried out in accordance with the ethical and scientific standards outlined in national and international guidelines.

2.11 Operational Definitions (1,6)

Cervical tissue specimens: specimens of patients with cervical complaints such as punch biopsies and hysterectomy specimens.

Cancerous cervical lesion: malignant lesions of the cervix.

Histopathologic pattern: a specific type of diagnosis made on biopsy specimens.

Noncancerous cervical lesion: benign conditions of the cervix.

Precancerous cervical lesion: precursor lesions of cervical cancer (LSIL and HSIL)

2.12 Limitations of the Study

Biopsy results are not computerized and compiled with the clinical data of patients; thus, most of the factors associated with cervical cancer cannot be assessed. Confirmatory immunohistochemistry and HPV DNA testing could not be included in this study because of the limited setup. Power analysis for sample size calculation was not performed.

3. Results and discussions

3.1. Results

In 2018–2019 (n = 1838) and 2019–2020 (n = 1928), a total of 3766 biopsy specimens (2613 female and 1153 male) were submitted to the pathology department. From 2613 female biopsy reports, 543 biopsy reports of cervical lesion specimens were extracted, of which 74 were excluded because they fulfilled the exclusion criteria. Cervical biopsy specimens accounted for 14.4% of the total biopsy specimens and 20.8% of the total biopsy specimens from female patients who submitted biopsy specimens during the study period.

3.1.1. Sociodemographic profiles

Of the 469 cervical lesion cases, 244 cases (52.03%) were from 2018–2019 and 225 (47.97%) cases were from 2019–2020 (Figure 2).

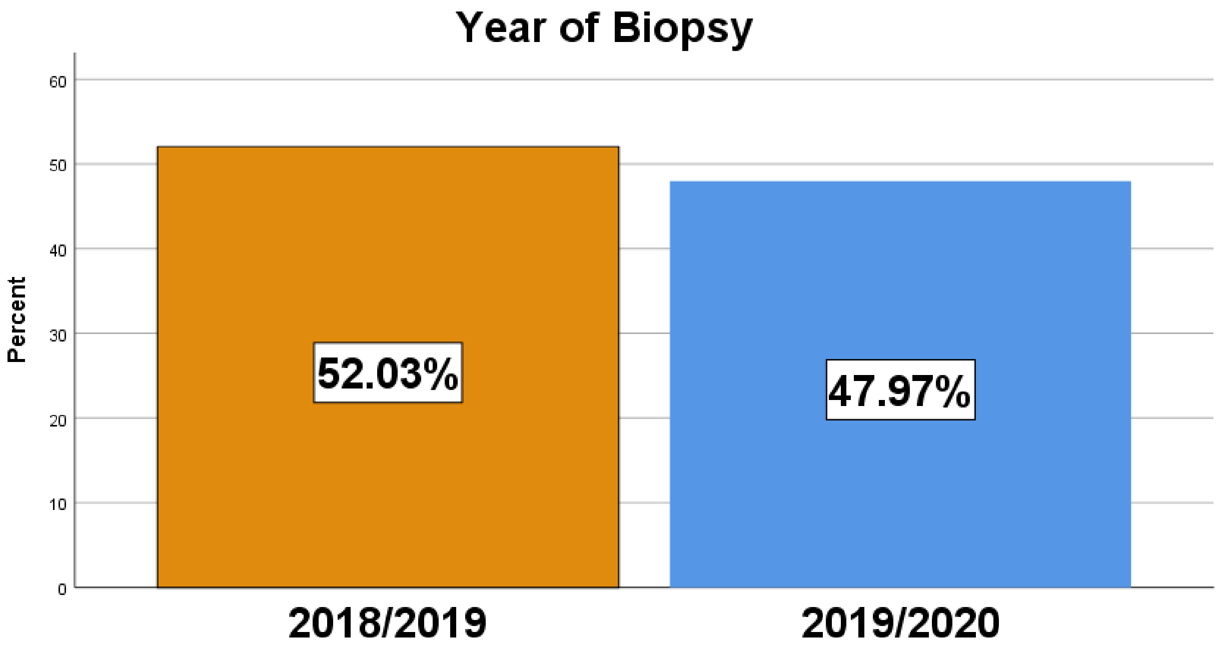

Figure 2: Bar graph showing the distribution of cervical lesions between 2018/2019 and 2019/2020

The age distributions have a minimum value of 22 years and a maximum value of 85 years, with a mean age of 47.06 years and a standard deviation of 11.019 years. The maximum age distribution of cervical lesions was in the 41–50-year age range, accounting for 154 (32.8%) biopsies, followed by 31–40 years with 143 (30.5%) biopsies, and 51–60 years with 102 (21.7%) biopsies.

More than half of precancerous and cancerous cervical lesions (65.46%) occurred after the age of 40 years, while benign lesions accounted for 47.5% after the age of 40 years. This was statistically significant. (X^2=23.54, DF =5 *p*=**0.001**).

As the patient's age increases, the risk of precancerous and cancerous cervical lesions increases, and the third and fourth decades of life were found to be associated with an increased risk of having precancerous and cancerous cervical lesions (Figure 3).

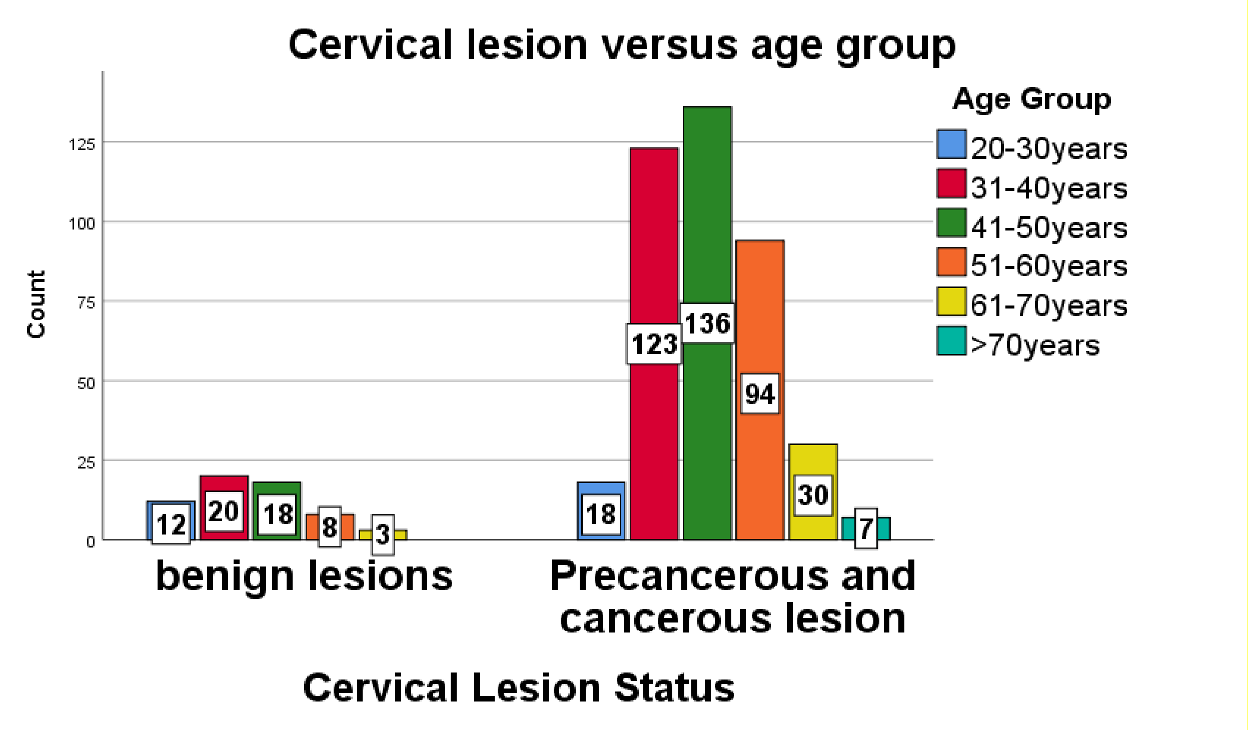

Figure 3: Bar graph showing cervical lesion status with age groups

Most of the patients (77.8%) were from surrounding areas with variable distances from Jimma town, while 104 (22.2%) were from Jimma town. The majority of patients coming from the periphery were from the Jimma zone, accounting for 58.4% (Figure 4). Most (88.8%) of the cases from the surrounding areas of Jimma were precancerous and cancerous cervical lesions, while 80.8% of the cases in Jimma town were precancerous and cancerous cervical lesions. This showed a strong association between precancerous and cancerous cervical lesions and residency. (X2 = 4.67, DF = 1, p = 0.027).

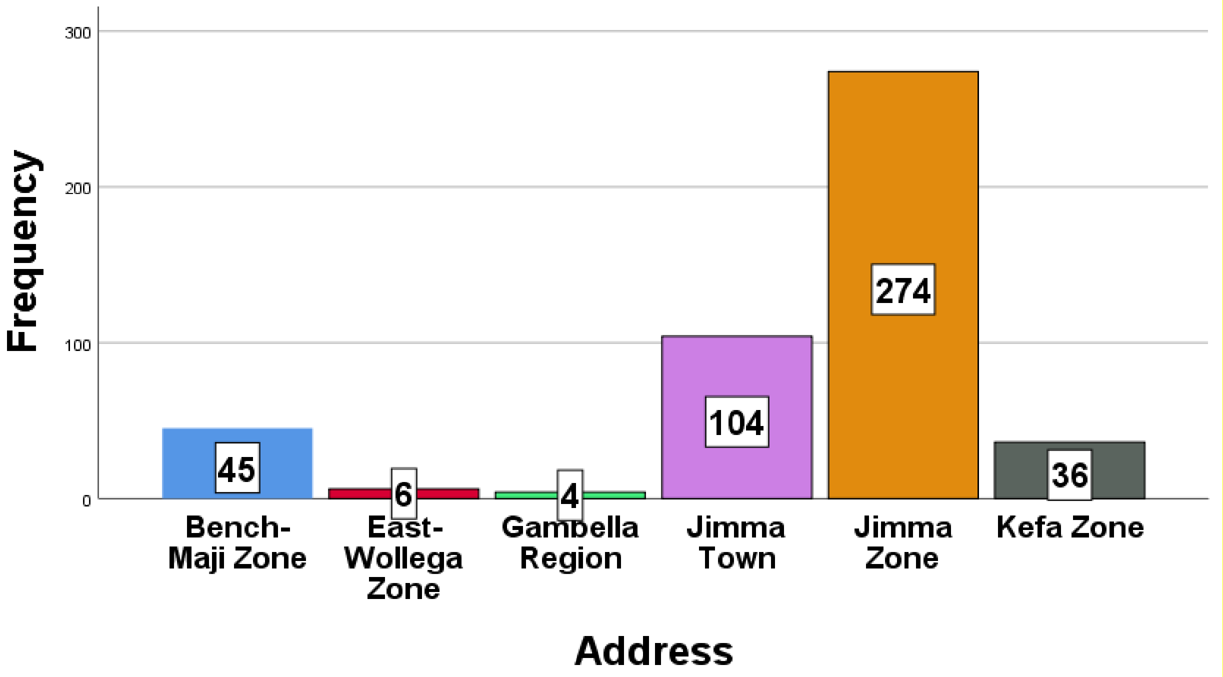

Figure 4: Bar graph showing the distribution of cervical lesions among residents of Jimma town and surrounding areas

3.1.2. Clinical data of patients with cervical lesions

The most common presenting symptom of patients with cervical lesions was vaginal bleeding alone, accounting for 206 (43.9%) patients, followed by vaginal bleeding with vaginal discharge (108 (23%) patients) and vaginal bleeding with lower abdominal pain (89 (19%) patients). (Table 1). The duration of symptoms in patients with cervical lesions ranged from four days up to sixteen years (Figure 5). The most common type of biopsy performed for patients with cervical lesions was a punch biopsy, accounting for 91.3% followed by abdominal hysterectomy, accounting for 6.4% (Table 2). Most (90%) patients with cervical complaints were clinically diagnosed with cervical cancer (Figure 6)

Table 1: Clinical features/presentation of patients with cervical lesions

Clinical features/presentation	Frequency	Percent
Vaginal Bleeding	226	48.2
Vaginal Bleeding and Vaginal Discharge	108	23.0
Vaginal Bleeding and Lower abdominal pain	89	19.0
Vaginal Discharge	44	9.4
Cervical CA Screening	2	0.4
Total	469	100.0

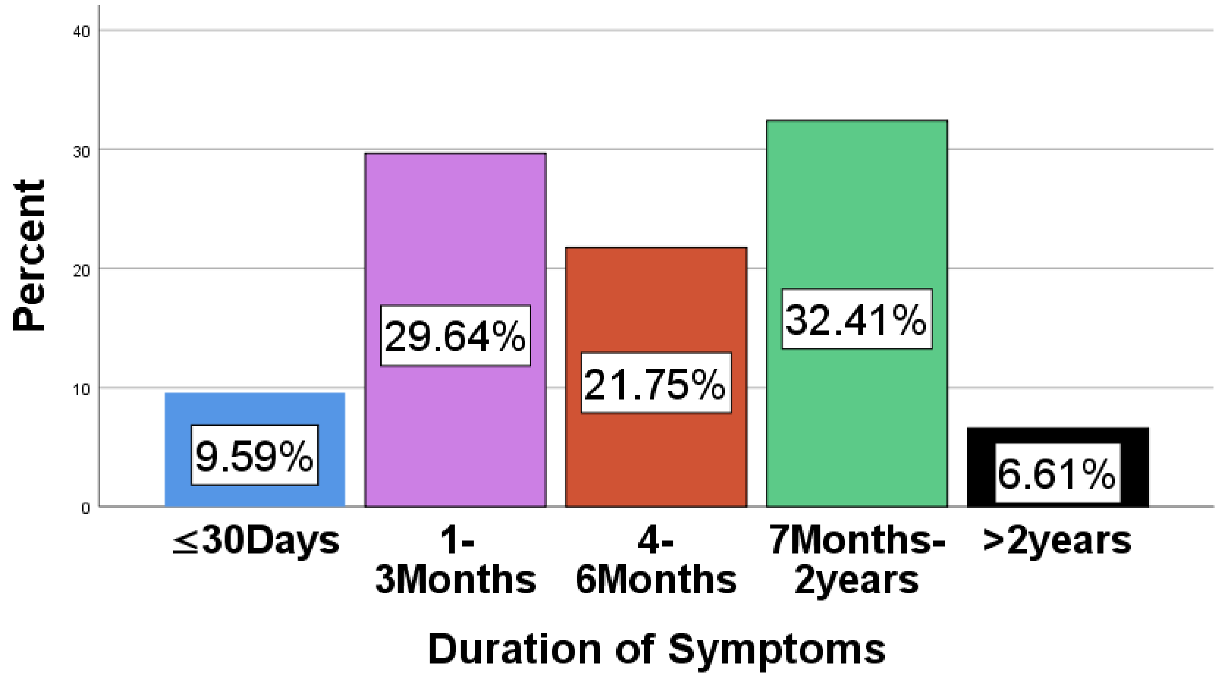

Figure 5: Bar graph showing the distribution of the duration of symptoms in patients with cervical lesions

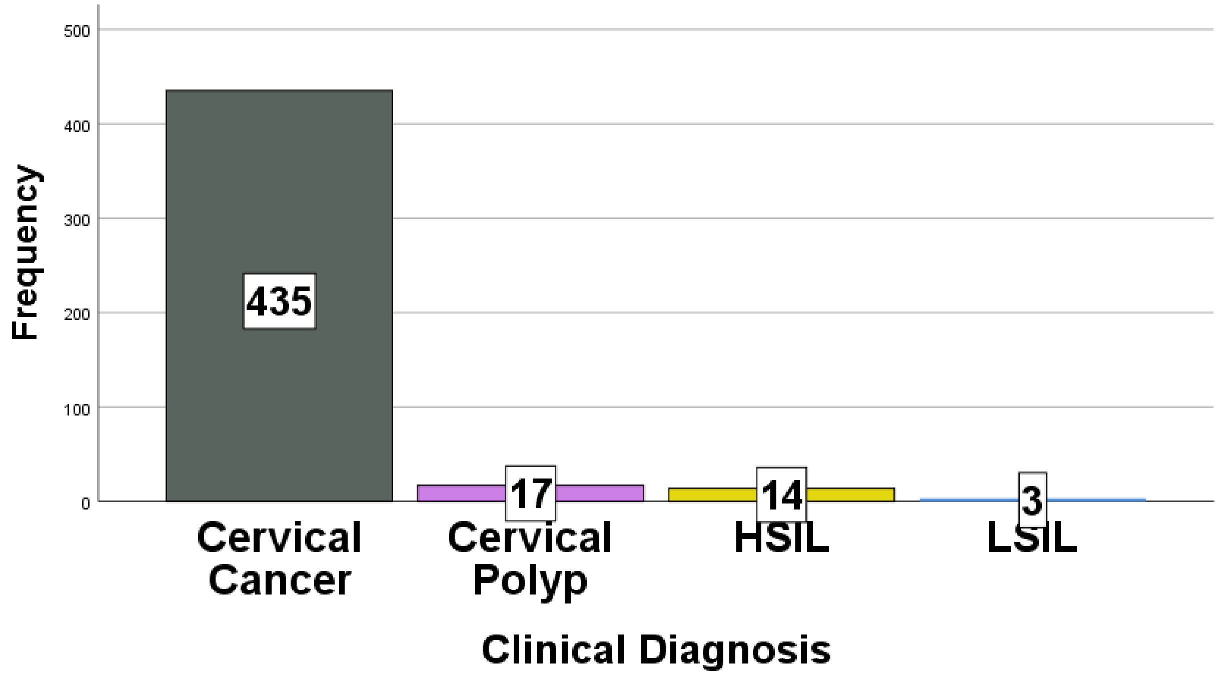

Figure 6: Bar graph showing the distribution of clinical diagnosis of patients with cervical lesions

Table 2: Type of biopsy of patients with cervical lesions

Type of Biopsy/Nature of specimen	Frequency	Percent
Cone Biopsy	3	0.6
Hysterectomy	30	6.4
LEEP	8	1.7
Punch Biopsy	428	91.3
Total	469	100.0

3.1.3. Histopathologic patterns of cervical lesions

Most (86.99%) of the specimens were precancerous and cancerous cervical lesions, while 13.01% were benign cervical lesions (Figure 7). The majority (71%) of cases were cancerous cervical lesions, followed by precancerous cervical lesions (16.4%) (Table 3). Squamous cell carcinoma (SCC) was the most frequent cervical cancer diagnosed during the study period, accounting for 96.4% (Table 4). HSIL was the most frequently diagnosed precancerous lesion, accounting for 68.4%. Endocervical polyps were the most commonly diagnosed benign lesions, accounting for 59.3% (Table 3). The most common histologic subtype of SCC was keratinizing SCC, accounting for 77.4% of all cases, while the remaining 22.6% were nonkeratinizing SCC (Figure 8).

Table 3: Numbers and percentages of cervical lesions

**Type of Cervical Lesion**	**Frequency**	**Causes**	**Percentage**
Precancerous cervical lesion	79	54(68.4%) HSIL<br>25(31.6%) LSIL	16.8%
Cancerous cervical lesion	331	See Table 4	70.6%
Noncancerous cervical lesion	59	35(59.3%) Endocervical polyp<br>18(30.5%) Squamous metaplasia<br>6(10.2%) Cervicitis	12.6%
Total	469		100%

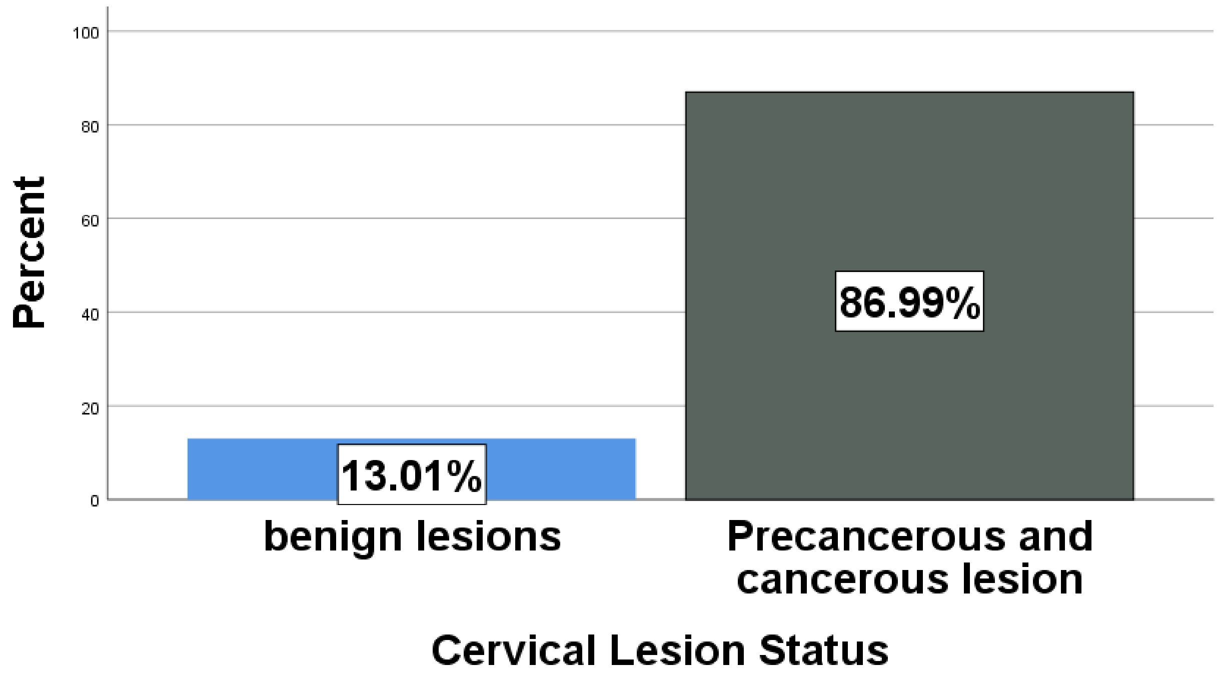

Figure 7: Bar graph showing the distribution of cervical lesion status

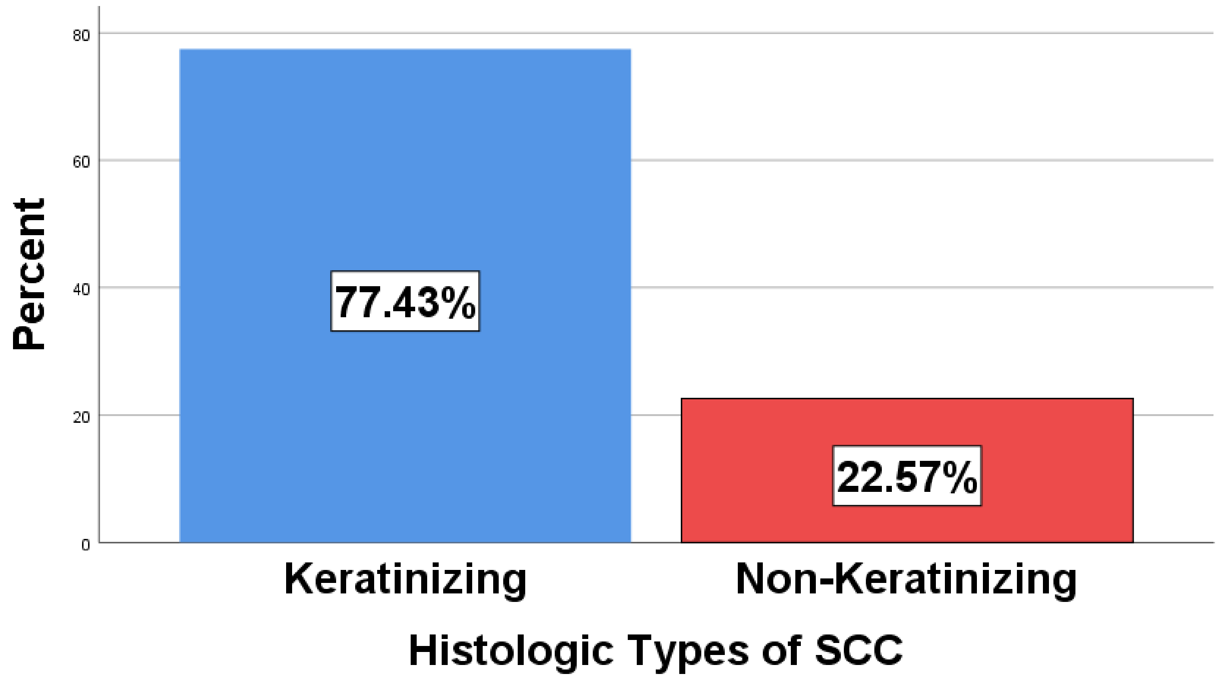

Figure 8: Bar graph showing histologic types of SCC

Table 4: Histopathological classification of cervical cancer

Histopathologic type	Frequency	Percentage
Squamous cell carcinoma	319	96.4%
Adenocarcinoma	11	3.3%
Adenosquamous carcinoma	1	0.3%
Total	331	100%

Cancerous cervical lesions tend to develop in older age groups, and benign conditions tend to develop in younger age groups. SCC occurred most frequently in the 41–50-year age group (N = 104), followed by the 31-40 year (N = 86) and 51–60-year age groups (N = 85). (Table 5)

Table 5: Pattern of the cervical lesion by age group

Age	Histopathologic Diagnosis								
Group	Cervicitis	Squamous Metaplasia	Endocervical Polyp	LSIL	HSIL	Squamous Cell Carcinoma	Adenocarcinoma	Adenosquamous Carcinoma	Total
20-30years	0	4	8	3	1	14	0	0	30
31-40years	2	6	13	11	23	86	1	1	143
41-50years	3	3	10	10	19	104	5	0	154
51-60years	0	3	4	1	6	85	3	0	102
>60years	1	2	0	0	5	30	2	0	40
Total	6	18	35	25	54	319	11	1	469

3.1.4. Factors associated with cervical lesions

Age (*p*=**0.001, COR=1.048**) and place of residence (*p*= **0.032, COR=0.531**) were tested at a P value less than 0.05 in bivariate logistic regression and selected as candidate variables for multivariate logistic regression. Cervical lesions were grouped into benign and HPV-related precancerous and cancerous lesions. Addresses were categorized into Jimma town, which is urban and surrounding areas, which were rural. Multivariate logistic regression analysis was performed on these variables using the step-wise method and the overall model was significant at a P value of 0.001. Age (*p*=**0.001, AOR=1.052**) and place of residency (*p*=**0.015, AOR=0.47**) were shown to be the independent predictors of precancerous and cancerous cervical lesions, hence older women are 1.052 times more likely to develop precancerous and cancerous cervical lesions than younger women and rural dwellers are 0.47 times more likely to develop precancerous and cancerous cervical lesions than urban dwellers which were statistically significant as shown in Tables 6,7 and 8.

Table 6: Bivariate logistic regression of cervical lesion status and age

Independent Variable	B-Coefficient	t-value	P-value	R square	COR
Age	0.154	3.369	0.001	0.024	1.048

COR: -Crude Odds Ratio

Table 7: Bivariate logistic regression of cervical lesion status and address

Independent Variable	Beta Coefficient	t-value	P-value	R square	COR
Address	0.099	2.145	0.032	0.010	0.531

COR: -Crude Odds Ratio

Table 8: Multivariate logistic regression of cervical lesion status and associated factors

Independent Variables	Beta Coefficient	t-value	P-value	AOR	R square
Age	0.162	3.561	0.001	1.052	0.036
Address	0.111	2.435	0.015	0.47	

AOR: - Adjusted Odds Ratio

3.2 Discussion

The most common type of biopsy performed for patients with cervical lesions was a punch biopsy, accounting for 91.3% followed by total abdominal hysterectomy accounting for 6.4%. In a study performed in India, of the total 110 cases, fifty-one were hysterectomy specimens including 25 Wertheim's hysterectomies, 16 total abdominal hysterectomies, and 10 vaginal hysterectomies. Fifty-nine specimens were cervical biopsies (19). Additionally, in a study done in Italy, charts of 224 "low-risk" patients were retrieved and, 50 patients undergoing radical hysterectomy were matched with 100 patients undergoing open radical hysterectomy(20) . Similarly, in a study done in Italy on precancerous cervical lesions, 2966 patients underwent conization for CIN2-3 during the study period (21). These and other studies performed in the best centers show a higher number of hysterectomies and conizations because of the well-established chemotherapy set-up for advanced cancers and early screening and the detection of precursor lesions. In the study area, there is a high number of punch biopsies than hysterectomies, because patients will be referred to black lion hospital, Addis Ababa, Ethiopia for further management once the diagnosis of cervical cancer is made by punch biopsies.

Cervical lesions tend to affect females across a wide age range. The age distributions have a minimum value of 22 years and a maximum value of 85 years, with a mean age of 47.06 years and a standard deviation of 11.019 years. This is consistent with Ameya G.'s study on 513 biopsies in Hawassa, Ethiopia, in which the age ranges were 17 to 85 years, with a mean and standard deviation of 42 and 11 years respectively (22). Our study is also similar to a study done on 306 cervical cancer cases in Nigeria, which showed patient ages diagnosed with cervical cancer to range from 18 to 85 years, with peak occurrence in the fifth decade (23). A study done on 1047 specimens in Lagos, Nigeria, also showed the age range of patients as 19–87 years, with a mean age of 49±13 (24). Another study done at TASH, A.A., Ethiopia on women with histologically verified cancer of the cervix uteri also showed that the mean age was 49 years (21–91 years) (25). Our findings are consistent with this and other similar studies.

Cervical lesions range from benign inflammatory conditions and polyps to malignant conditions. Of the 469 biopsies submitted to JMC, the department of pathology, most (87%) of the specimens were precancerous and cancerous cervical lesions, while 13% were benign cervical lesions. The majority (70.6%) of cases were cancerous cervical lesions, followed by precancerous cervical lesions (16.8%) and noncancerous cervical lesions (12.6%). A study performed in Malawi on 212 cervical lesions showed that 17% had precancerous lesions, 65% were cancerous, and 18% had both precancerous and cervical cancer, making a total of 83% cancerous lesions (26). Another study performed in India on 110 cases showed that a diagnosis of intraepithelial neoplasia was made in 32.7% of cases, and invasive carcinoma was found in 67.3% of cases (27). Therefore, the finding of our study has a slightly higher number of cancerous cases than this last finding and other similar studies. This is possibly because of the limited resources and services within the study area.

Squamous cell carcinoma (SCC) was the most frequent cervical cancer diagnosed during the study period, accounting for 96.4% of the 333 cancerous cases, followed by adenocarcinoma (3.3%) and adenosquamous carcinoma (0.3%). This is consistent with a study done at JUSH, Jimma, Ethiopia, on 154 cervical cancer cases, which showed that 91% of the cases were SCC, 5.84% were small cell carcinomas, 2.59% were adenocarcinomas, 0.64% were adenosquamous carcinomas (28). Our study is also consistent with a study performed in Cameron on 2078 cervical cancer cases, which showed that the most common cervical cancer was SCC (81.18%), followed by adenocarcinoma (12.95%) (29). In another study done at TASH, Addis Ababa, Ethiopia, cancer of the uterine cervix was found to be the most common malignancy among all gynecological malignancies (30). Another study done at A.A., Ethiopia on women with postmenopausal bleeding also showed that about 94% of the cervical malignancies were squamous cell carcinoma(31). A study done at Saint Paul Hospital Millennium Medical College (SPHMMC), Addis Ababa, Ethiopia, on breast and gynecologic malignancies also showed that SCC of the cervix was the most common type of cervical malignancy detected, accounting for 90% of all cases, while adenocarcinoma (3.85%) was the second most observed type of cervical cancer (32). Similarly, according to a study done in Bangladesh on gynecologic malignancy, SCC was the most common histopathologic type in cervical and vulvar cancers (33). Another study done in Sudan on 372 patients showed that SCC (NOS) was the most common subtype (76.6%) and adenocarcinoma was the second most common subtype (12.6%) (34).

On the contrary, according to a study performed in India over a period of 2 years, the most frequent condition diagnosed on cervical biopsies and hysterectomy specimens was benign cervical polyp (66.3%), followed by carcinoma (23.7%) and squamous intraepithelial lesions (0.2%)(35). These last studies and other studies performed in Western countries show a higher number of benign cervical lesions because of early screening and the detection of precursor lesions. Although cervical cancer can be prevented by vaccination and screening and cured if identified early, this is not yet fully implemented in the study area.

Of the 79 precancerous cervical conditions diagnosed during the study period, HSIL was the most frequently diagnosed precancerous lesion, accounting for 68.4%, with the other 31.6% being LSIL. This is consistent with a study performed in Hawassa, Ethiopia, on 513 biopsies, which showed that among precancerous cervical lesions, HSIL was the predominant lesion (39.8%) (22). Similarly, a study performed in India showed that cervical intraepithelial neoplasia (CIN) 1 was diagnosed in 36.1% of patients, followed by CIN 2 (33.3%) and CIN 3 (30.6%) (27).

Of the 59 benign cases, endocervical polyps were the most commonly diagnosed benign lesion, accounting for 59.3%, followed by squamous metaplasia (30.5%) and cervicitis (10.2%). A study performed in Nigeria on 176 cervical specimens showed that inflammatory lesions accounted for 55 cases (59.8%) of non-neoplastic cervical lesions, followed by endocervical polyps, which accounted for 15 cases (16.3%) of non-neoplastic cervical lesions. Among the inflammatory lesions, chronic nonspecific cervicitis was the most commonly encountered lesion, constituting 40 cases (72.2%) of all inflammation (36). In a study performed at TASH, Addis Ababa, Ethiopia chronic cervicitis was the commonest benign lesion (16.8%) (37). Contrary to this and other similar studies, our study has the lowest number of cervicitis cases, possibly because the majority of cervicitis cases are managed clinically without histopathological confirmation once a clinical diagnosis is made as per the National STD Guidelines.

4. Conclusion and recommendation

4.1. Conclusion

Cervical diseases include a wide range of disorders and occur across different age groups. The age distributions have a minimum value of 22 years and a maximum value of 85 years, with a mean age of 47.06 years. The most common presenting symptom in patients with cervical lesions was vaginal bleeding. The most common type of biopsy performed for patients with cervical lesions is a punch biopsy. Most (90%) patients with cervical complaints were clinically diagnosed with cervical cancer. Cervical lesions range from benign inflammatory conditions and polyps to malignant conditions. Most cervical lesions were precancerous and cancerous. Squamous cell carcinoma (SCC) was the most frequent type of cervical cancer. HSIL was the most frequently diagnosed precancerous cervical lesion. Endocervical polyps were the most commonly diagnosed benign cervical lesion. Precancerous and cancerous cervical lesions tend to increase with increasing age and in those patients who come from peripheral areas.

4.2. Recommendation

There was a rising burden of cervical cancer. This implies that there is a need to educate the community to improve health-seeking behavior and on possible preventive strategies for cervical cancer. It is also strongly recommended that JMC's Department of Pathology commence cervical cytological examinations to detect dysplasia before it turns into full-blown cancer. We recommend that the Federal Ministry of Health strengthen the ongoing nationwide HPV vaccinations, screenings, and interventions for cervical cancer at a much faster rate.

Acknowledgment

I thank Jimma University for allowing me to undertake the research. I extend my gratitude to Jimma Medical Center, Department of Pathology, for providing me with the necessary resources. I also thank my advisors and friends for giving me constructive comments and important information to prepare my proposal and thesis. Last but not least, my warm gratitude goes to all my family members for providing me with emotional support during this study.

References

1. John Goldberg LLJMcKenney. “Rosai and Ackerman’s Surgical Pathology” 11th eds. In: 11th ed. Philadelphia: Elsevier, 2018. p. 1260–93.
2. Iqbal B, Masood I, Aiman A, Yasir M, Mushtaq I, Verma V. Study of Metaplastic Lesions of Different Parts of The Female Genital Tract: A Prospective Study. Internet Journal of Pathology. 2010 Jul;
3. Kumar V., Abbas K. AJ. Robbins, and Cotrans Pathologic Basis of Diseases 9th ed. 9th edition. Philadelphia: Elsevier; 2015. 1001–1007 p.
4. Robboy SJ. Robboy’s Pathology of the Female Reproductive Tract [Internet]. Elsevier Health Sciences; 2009 [cited 2023 Jun 6]. 1001–1007 p. Available from: https://books.google.com.et/books?hl=en&lr=&id=ab545XL-MBEC&oi=fnd&pg=PR29&dq=3.+Robboy+S.,+Mutter+G.,Prat+J.,Et+al.+Robboy%27s+Pathology+of+Female+Reproductive+Tract.+2nd+Edition.+Philadelphia+:+Elsevier,+2009,+pp.+189-295.&ots=d4zyLlxZJo&sig=kp7dnNNhqn3zRa944GvDuN5EvYs&redir_esc=y#v=onepage&q&f=false
5. Fletcher CDM. Diagnostic Histopathology of Tumors. In: 4th ed. Philadelphia: Elsevier; 2013 [cited 2023 Jun 6]. p. 814–38. Available from: https://shop.elsevier.com/books/diagnostic-histopathology-of-tumors-2-volume-set/fletcher/978-0-323-42860-6
6. Herrington CS, Board WC of TE. Female Genital Tumours [Internet]. WHO Female Genital Tumours. 2020 [cited 2023 Jun 6]. 632 p. Available from: https://www.research.ed.ac.uk/en/publications/who-classification-of-tumours-female-genital-tumours
7. Ikechebelu JI, Onyiaorah I V., Ugboaja JO, Anyiam DCD, Eleje GU. Clinicopathological analysis of cervical cancer seen in a tertiary health facility in Nnewi, south-east Nigeria. http://dx.doi.org/103109/01443610903531394 [Internet]. 2010 Apr [cited 2023 Jun 6];30(3):299–301. Available from: https://www.tandfonline.com/doi/abs/10.3109/01443610903531394
8. Ethiopian Federal Ministry of Health. FEDERAL MINISTRY OF HEALTH ETHIOPIA DISEASE PREVENTION AND CONTROL DIRECTORATE NATIONAL CANCER CONTROL PLAN [Internet]. 2016 [cited 2023 Jun 6]. Available from: https://www.google.com/url?sa=D&q=https://www.iccp-portal.org/sites/default/files/plans/NCCP%2520Ethiopia%2520Final%2520261015.pdf&ust=1686138720000000&usg=AOvVaw0eDMP3rQOPzm9DhCUtnBPJ&hl=en&source=gmail
9. Ferlay J, Shin HR, Bray F, Forman D, Mathers C, Parkin DM. Estimates of worldwide burden of cancer in 2008: GLOBOCAN 2008. Int J Cancer [Internet]. 2010 Dec 15 [cited 2023 Jun 6];127(12):2893–917. Available from: https://onlinelibrary.wiley.com/doi/full/10.1002/ijc.25516
10. Ferlay J, Colombet M, Soerjomataram I, Mathers C, Parkin DM, Piñeros M, et al. Estimating the global cancer incidence and mortality in 2018: GLOBOCAN sources and methods. Int J Cancer. 2019 Apr 15;144(8):1941–53.
11. Ferlay J, Soerjomataram I, Dikshit R, Eser S, Mathers C, Rebelo M, et al. Cancer incidence and mortality worldwide: Sources, methods and major patterns in GLOBOCAN 2012. Int J Cancer. 2015 Mar 1;136(5): E359–86.

12. Information Centre on HPV and Cancer. Human Papillomavirus and Related Diseases Report WORLD [Internet]. Available from: www.hpvcentre.net
13. Deressa BT, Cihoric N, Badra EV, Tsikkinis A, Rauch D. Breast cancer care in northern Ethiopia – cross-sectional analysis. BMC Cancer [Internet]. 2019 Apr 25 [cited 2023 Jun 6];19(1). Available from: /pmc/articles/PMC6485046/
14. Memirie ST, Habtemariam MK, Asefa M, Deressa BT, Abayneh G, Tsegaye B, et al. Estimates of cancer incidence in Ethiopia in 2015 using population-based registry data. J Glob Oncol. 2018 Mar 1;2018(4):1–11.
15. Ntekim A, Ntekim A. Cervical Cancer in Sub Sahara Africa. Topics on Cervical Cancer with an Advocacy for Prevention [Internet]. 2012 Mar 2 [cited 2023 Jun 6]; Available from: https://www.intechopen.com/chapters/30747
16. Census 2007. Stastical Oromia [Internet]. [cited 2023 Jun 6]. Available from: http://www.csa.gov.et/census-report/complete-report/census-2007?start=5
17. Jimma University. Home – JU Official Website [Internet]. [cited 2023 Jun 6]. Available from: https://ju.edu.et/
18. Mapcarta. Jimma University Map - University - Ethiopia - Mapcarta [Internet]. [cited 2023 Jun 6]. Available from: https://mapcarta.com/33099124
19. Gupta M, Basavaraj P. Histopathological Spectrum of Premalignant and Malignant Lesions of Uterine Cervix. National Journal of Laboratory Medicine [Internet]. 2018; 7:8. Available from: http://files/346/Gupta y Basavaraj - 2018 - Histopathological Spectrum of Premalignant and Mal.pdf
20. Di Donato V, Bogani G, Casarin J, Ghezzi F, Malzoni M, Falcone F, et al. Ten-year outcomes following laparoscopic and open abdominal radical hysterectomy for "low-risk" early-stage cervical cancer: A propensity-score based analysis. Gynecol Oncol. 2023 Jul; 174:49–54.
21. Giannini A, Di Donato V, Sopracordevole F, Ciavattini A, Ghelardi A, Vizza E, et al. Outcomes of High-Grade Cervical Dysplasia with Positive Margins and HPV Persistence after Cervical Conization. Vaccines (Basel). 2023 Mar 18;11(3).
22. Ameya G, Yerakly F. Characteristics of cervical disease among symptomatic women with histopathological sample at Hawassa University referral hospital, Southern Ethiopia. BMC Womens Health [Internet]. 2017 Sep 29 [cited 2023 Jun 6];17(1):1–7. Available from: https://link.springer.com/articles/10.1186/s12905-017-0444-5
23. Silas OA, Yakubu D, Jegede OO, Ajetunmobi OI, Mosugu O. A histopathologic review of cervical cancers in Jos University Teaching Hospital, Jos, Nigeria. Annals of Tropical Pathology [Internet]. 2018 [cited 2023 Jun 6];9(2):135. Available from: https://www.atpjournal.org/article.asp?issn=2251-0060;year=2018;volume=9;issue=2;spage=135;epage=138;aulast=Silas
24. Faduyile F, Soyemi S, Wright K, Osuolale F. Histopathological study of surgical cervical biopsies in Lagos, Nigeria. Trop J Obstet Gynaecol. 2017;34(2):124.
25. Kantelhardt EJ, Moelle U, Begoihn M, Addissie A, Trocchi P, Yonas B, et al. Cervical Cancer in Ethiopia: Survival of 1,059 Patients Who Received Oncologic Therapy. Oncologist [Internet]. 2014 Jul 1 [cited 2023 Jun 6];19(7):727–34. Available from: https://academic.oup.com/oncolo/article/19/7/727/6399365
26. Bangara FF, Marimo C, Kaile T, Simakando M, Tomoka T, Arabi G, et al. Histological Characterization of Invasive Cervical Cancer and Precursor Lesions in Malawian Women

Presenting at Queen Elizabeth Central Hospital (QECH): A Cross-Sectional Study. J Virol Antivir Res. 2017;06(01).
27. Mamta Gupta PB. Histopathological Spectrum of Premalignant and Malignant Lesions of Uterine Cervix. National Journal of Laboratory Medicine [Internet]. 2018 [cited 2023 Jun 6];7(1). Available from: DOI: 10.7860/NJLM/2018/32340:2276
28. Saleem A, Bekele A, Fitzpatrick MB, Mahmoud EA, Lin AW, Velasco HE, et al. Knowledge and awareness of cervical cancer in Southwestern Ethiopia is lacking: A descriptive analysis. PLoS One [Internet]. 2019 Nov 1 [cited 2023 Jun 6];14(11): e0215117. Available from: https://journals.plos.org/plosone/article?id=10.1371/journal.pone.0215117
29. Paul ENJ, Charlotte TN, Jules OML, Godefroy S, Daniel ESJ, Michelle EFA, et al. Epidemiological and Histological Profile of Cervical Cancer in Cameroon: About 2078 Cases. Open J Obstet Gynecol [Internet]. 2016 [cited 2023 Jun 6];06(04):232–9. Available from: http://file.scirp.org/Html/
30. Tigeneh W, Molla A, Abreha A, Assefa M. Pattern of Cancer in Tikur Anbessa Specialized Hospital Oncology Center in Ethiopia from 1998 to 2010. Int J Cancer Res Mol Mech [Internet]. 1998;1. Available from: http://dx.doi.org/10.16966/2381-3318.103
31. Ergete W, Tesfaye A, Health EJ. The Ethiopian Journal of Health Development Histopathological findings of post-Menopausal bleeding in Ethiopian women.
32. Hailu HE, Mondul AM, Rozek LS, Geleta T. Descriptive Epidemiology of breast and gynecological cancers among patients attending Saint Paul’s Hospital Millennium Medical College, Ethiopia. PLoS One [Internet]. 2020 [cited 2023 Jun 6];15(3): e0230625. Available from: https://journals.plos.org/plosone/article?id=10.1371/journal.pone.0230625
33. Rahman MA, Siddika ST, Mazid M. Gynaecological Cancers in Surgical Specimens – A Hospital Based Analysis. Med Today. 2015 Jul 16;26(2):78–82.
34. Rayis DAM, Zulfu A, Yassin K, Merghani A. Histopathological pattern of gynecological malignancies: National Health Laboratory (NHL), Sudan. GPH - International Journal of Health Sciences and Nursing [Internet]. 2018 Aug 30;1(1). Available from: http://gphjournal.org/index.php/hsn/article/view/45
35. Atul J. RJ, IE al. Histopathological study of tumors of Cervix at MGM Medical College. Medical College of India. 2014;1(1).
36. Forae G, Nwachokor F. Morphological spectrum of non-neoplastic lesions of the uterine cervix in Warri, South-South, Nigeria. Niger J Clin Pract. 2013;16(4):429.
37. Nigatu B, Gebrehiwot Y, Kiros K, Ergete W. Number 1 Ethiopian Journal of Reproductive Health.

Title: Histopathologic patterns and factors associated with cervical lesions at Jimma Medical Center, Jimma, Southwest Ethiopia: A two-year cross-sectional study

Corresponding Author: -
Dr Birhanu Hailu Tirkaso (birhanuhailu@mtu.edu.et**)**
Co-Authors: -
Dr. Tesfaye Hurgesa Bayisa (tesfayehurgesa@ju.edu.et**)**
Dr. Tewodros Wubshet Desta (theddo04@gmail.com)
Date: 31 July 2023

Author's Response to Editor and Reviewers

Dear PLOS ONE Academic Editor and Reviewers,

We would like to thank you for the in-depth review of the manuscript and constructive comments. We, the authors of the manuscript entitled "**Histopathologic patterns and factors associated with cervical lesions at Jimma Medical Center, Jimma, Southwest Ethiopia: A two-year cross-sectional study**" want to appreciate PLOS ONE editorial policy and peer review process in the publication of a quality scientific paper. The responses to the comments and requests are indicated as follows and the revised manuscript is uploaded as a separate highlighted manuscript labeled 'Revised Manuscript with Track Changes' and a clean revised document labeled 'Manuscript' as requested.

Responses

1. The manuscript is now revised according to PLOS ONE's style requirements in accordance with the formatting guidelines provided. We have updated the title page and renamed the chapters as outlined in the PLOS ONE guidelines. Figures were also formatted according to the guidelines and included within the main document as before. The figures will also be uploaded as a separate file in case needed during manuscript processing.
2. Regarding the minor occurrence of overlapping text with previous publications, we revised the specific paragraphs and rephrased the text to avoid plagiarism and we have cited the paper mentioned by the academic editor. Please see pages 1-3. The revised manuscript is checked for plagiarism using online checkers and checked for similarity index using Turnitin before resubmission. The majority of the similarity index came from the same thesis of the corresponding author put on the institutional website by Jimma University to avoid topic redundancy for future graduates but was not formally published. One thing, we want to clarify is that this manuscript is the refined version of the manuscript posted on Jimma University's website: https://repository.ju.edu.et/handle/123456789/4080.
3. Concerning dual publications, the manuscript was not peer-reviewed and formally published anywhere. As a part of second-degree completion, Jimma University put the thesis of postgraduate graduates on its website so that each graduate student selects a unique research topic.

We put the link to the institutional repository to indicate that our work is based on the material posted on the website. Because of this, this work does not constitute a dual publication.

4. The other issue raised was the data availability statement. All relevant data are within the manuscript and its Supporting Information files. Please update this for us.
5. In response to Reviewer #1, Pie charts have been replaced by bar graphs. Pages 9 and 11
6. In response to Reviewer #2, the discussion part has been expanded now to include the type of biopsies (Nature of specimen) by incorporating additional references. Page 17
