# Supplementary material for: Histopathologic patterns and factors associated with cervical lesions at Jimma Medical Center, Jimma, Southwest Ethiopia: A two-year cross-sectional study
Source: PLoS One. 2024 Apr 18;19(4):e0301559. doi: 10.1371/journal.pone.0301559 (PMC11034979; doi:10.1371/journal.pone.0301559)
Supplement: Attachment — Submitted filename: Response to Reviewers.docx [file pone.0301559.s005.docx]

**Title:** **Histopathologic patterns and factors associated with cervical lesions at**

**Jimma Medical Center, Jimma, Southwest Ethiopia: A two-year cross-sectional study**

**Corresponding Author: -**

**Dr Birhanu Hailu Tirkaso (**birhanuhailu@mtu.edu.et**)**

**Co-Authors: -**

**Dr. Tesfaye Hurgesa Bayisa (**tesfayehurgesa@ju.edu.et**)**

**Dr. Tewodros Wubshet Desta (**[**theddo04@gmail.com**](mailto:theddo04@gmail.com)**)**

**Date: 31 July 2023**

**Author's Response to Editor and Reviewers**

Dear PLOS ONE Academic Editor and Reviewers,

We would like to thank you for the in-depth review of the manuscript and constructive comments. We, the authors of the manuscript entitled “**Histopathologic patterns and factors associated with cervical lesions** **at Jimma Medical Center, Jimma, Southwest Ethiopia: A two-year cross-sectional study**” want to appreciate PLOS ONE editorial policy and peer review process in the publication of a quality scientific paper. The responses to the comments and requests are indicated as follows and the revised manuscript is uploaded as a separate highlighted manuscript labeled 'Revised Manuscript with Track Changes' and a clean revised document labeled 'Manuscript' as requested.

Responses

1. The manuscript is now revised according to PLOS ONE's style requirements in accordance with the formatting guidelines provided. We have updated the title page and renamed the chapters as outlined in the PLOS ONE guidelines. Figures were also formatted according to the guidelines and included within the main document as before. The figures will also be uploaded as a separate file in case needed during manuscript processing.
2. Regarding the minor occurrence of overlapping text with previous publications, we revised the specific paragraphs and rephrased the text to avoid plagiarism and we have cited the paper mentioned by the academic editor. Please see pages 1-3. The revised manuscript is checked for plagiarism using online checkers and checked for similarity index using Turnitin before resubmission. The majority of the similarity index came from the same thesis of the corresponding author put on the institutional website by Jimma University to avoid topic redundancy for future graduates but was not formally published. One thing, we want to clarify is that this manuscript is the refined version of the manuscript posted on Jimma University's website:  <https://repository.ju.edu.et/handle/123456789/4080>.
3. Concerning dual publications, the manuscript was not peer-reviewed and formally published anywhere. As a part of second-degree completion, Jimma University put the thesis of postgraduate graduates on its website so that each graduate student selects a unique research topic.

We put the link to the institutional repository to indicate that our work is based on the material posted on the website. Because of this, this work does not constitute a dual publication.

4. The other issue raised was the data availability statement. All relevant data are within the manuscript and its Supporting Information files. Please update this for us.
5. In response to Reviewer #1, Pie charts have been replaced by bar graphs. Pages 9 and 11
6. In response to Reviewer #2, the discussion part has been expanded now to include the type of biopsies (Nature of specimen) by incorporating additional references. Page 17
