# Supplementary material for: Histopathologic patterns and factors associated with cervical lesions at Jimma Medical Center, Jimma, Southwest Ethiopia: A two-year cross-sectional study
Source: PLoS One. 2024 Apr 18;19(4):e0301559. doi: 10.1371/journal.pone.0301559 (PMC11034979; doi:10.1371/journal.pone.0301559)
Supplement: Attachment — Submitted filename: Response to Reviewers.docx [file pone.0301559.s007.docx]

**Title:** **Histopathologic patterns and factors associated with cervical lesions at**

**Jimma Medical Center, Jimma, Southwest Ethiopia: A two-year cross-sectional study**

**MANUSCRIPT ID: PONE-D-23-17869**

**Corresponding Author: -**

**Dr Birhanu Hailu Tirkaso (**birhanuhailu@mtu.edu.et**)**

**Version: -3**

**Date: 7 February 2024**

Dear PLOS ONE Academic Editor and Reviewers,

We would like to express our gratitude to all the reviewers and the editor for their thorough evaluation of our manuscript and constructive feedback. As the corresponding author of the manuscript entitled **" Histopathologic patterns and factors associated with cervical lesions at Jimma Medical Center, Jimma, Southwest Ethiopia: A two-year cross-sectional study**," I would like to confirm that we have addressed all the queries and comments provided by your team to the best of our ability. I have incorporated the necessary changes and made revisions accordingly, which can be found in the separate highlighted manuscript labeled 'Revised Manuscript with Track Changes' and a clean revised document labeled 'Manuscript' as requested.

Responses

1. We would like to thank the editor for inviting additional reviewers for an in-depth review of the manuscript. We updated our reference list papers by removing papers that have been retracted (Atul J. RJ, IE al. Histopathological study of tumors of Cervix at MGM Medical College. Medical College of India. 2014;1(1) and Nigatu B, Gebrehiwot Y, Kiros K, Ergete W. Number 1 Ethiopian Journal of Reproductive Health) and replacing them with relevant current references which are highlighted on the revised manuscript, to ensure that it is complete and correct.
2. Thank you very much Reviewer 2 for acknowledging the improvement and recommending the manuscript for publication, I am eternally grateful.
3. Thank you, Reviewer 3, for your evaluation and confirming that our manuscript falls within the aim of the Journal and is well-written. I am extremely grateful.
4. Thank you, Reviewer 4, for your review. I am grateful and have addressed your comments below.

- The findings of our study are quite similar to those from developing countries. This is mainly because the patients in our study tended to present late and were not vaccinated against the Human Papillomavirus (HPV). To establish detailed causal relationships between the findings and other factors, further studies involving genetic and molecular testing would be required. However, such studies are beyond the scope of our present research.
- Although it is generally accepted that a larger sample size of patients would lead to more reliable results, in this particular case, we only had access to well-documented hard-copy biopsy reports that were two years old at the time of data collection. Therefore, all 469 eligible reports were included in the study.
- The document underwent a thorough review for grammar and punctuation by both language software and a colleague who is proficient in written English.

5. Thank you, Reviewer 5, for your detailed review of the manuscript. I am grateful and have outlined my responses below.

- The idea of using representative sampling is valid and initially, we planned to use simple random sampling to select a representative sample of biopsy reports from the available 543 reports. The expected sample size was 226, after adjusting using a correction formula, but we later decided to include all 469 eligible reports (see Figure 1, page 6) in the study based on the advice of our colleagues from the Epidemiology side and a review of the literature, as outlined in our manuscript's references. This approach gave us a margin error of 1.67%, which we deemed acceptable. We were aware that having a sample that is too big or too small can waste resources or lead to unreliable results. We also needed to include small but significant factual information like the two cases of cervical screening, which made the sample size of 469 feasible for resource management. However, reviewer number 4 suggested increasing the study units, which we agree with, but there were no well-documented biopsy reports other than 2017/18 and 2018/19 at the time of the study.
- The previous dates mentioned in our manuscript have been corrected. The period of 12th September 2018 to 11th September 2020 has been adjusted to 12th September 2017 to 12th September 2019, and the data collection period has been corrected from May 1 to June 30, 2019, to May 1 to June 30, 2020. I apologize for any confusion this may have caused. The mistake occurred when I converted the Ethiopian calendar into the Gregorian calendar. I also want to clarify that the data was collected from two-year reports. It is important to note that the Ethiopian calendar lags behind the Gregorian calendar by 7 to 8 years, depending on the month, and that all activities of the pathology department are renewed in September, which marks the beginning of a new year in Ethiopia. Thank you for bringing this to our attention.
- I have noted your request regarding the issue of calculating the sample size for the third specific objective. Please note that our study is descriptive and we are not establishing causality. We are simply observing that certain factors that have been well-studied in the literature and textbooks could influence the frequency of cervical cancer, which varies with age group. This pattern has been reported in several studies, including one conducted by Ameya and his colleague from Hawassa, Ethiopia, which matches our report (Ameya & Yerakly, 2017). Another study conducted in Jimma, Ethiopia, also revealed that women above 50 years old were more likely to have advanced cervical lesions (Tesfaw et al., 2020). We have cited these papers as examples from the references list in our manuscript (**see reference numbers 22 and 26**) because they have been published in well-known journals. We encourage the comparison of the methodology parts of the second article, as it includes bivariate and multivariate logistic regressions, and was done on the same study area of our paper.
- Our research is based solely on the pathology report data. We were unable to collect clinical data from patient cards as we do not have access to their medical records without consent from the medical record service areas. However, the biopsy request format of Jimma Medical Center, which is included as supporting documents, contains all the variables necessary for our study. I created the questionnaire based on the biopsy requests and reports. It is important to note that we did not include all aspects of cervical cancer, such as parity, marital status, sexual history, HIV status, etc. This is why the title of our study is Histopathologic Patterns, rather than Clinicopathologic Patterns. **See Biopsy Form of JMC.**
- I understand that there is a comment regarding the statistical analysis of our paper that needs major revision. However, we have already undergone a rigorous review process and have presented refined versions of the statistical output. Additionally, the methodology section of our report is now expanded to include the logistic regression model and the formula we used. To run the regression, we utilized SPSS software. However, we did not include detailed statistical and analytical steps in the manuscript, as we presumed that readers understand how the output results are obtained. Also, we received feedback from reviewer 8 who suggested that we contextualize the model instead of providing a technical explanation. Our findings indicate that age and place of residency are independent predictors of precancerous and cancerous cervical lesions. This is clearly stated on page 16 of our paper, and all SPSS values have been included in the tables. In addition, we have added more references to the discussion section. If you could provide us with specific guidance on statistical issues such as P-values, COR, and AOR, we would be happy to make changes or provide explanations for our interpretations. Thank you. **See pages 6,7,16, 17, and 19**
- We have acknowledged some limitations of our study to ensure that readers consider them when interpreting the results. However, we have emphasized the strength of the study by providing a detailed description of the study area, including its large catchment area and the availability of pathology services. During the planning phase, we conducted a SWOT analysis and developed a conceptual framework, but we have only included selected concepts in this article based on the journal's requirements.
- I appreciate your recognition of the fact that only two out of the 469 patients came for screening purposes in our study. This was because PAP smear tests weren't available in the study area at the time of data collection. However, our study findings have been submitted to JMC's Department of Pathology with a strong recommendation to commence cervical cytological examinations to detect dysplasia before it turns into full-blown cancer. As a result, the department has started providing PAP services now, and we expect the situation to be different in future research in the study area. **See page 19**

6. Thank you, Reviewer 6, for appreciating the objective of the study and for your insightful comments, the responses and explanations are provided below.

- The idea of using sampling and sampling formula is valid and initially, we planned to use Yamane’s formula and simple random sampling to select a representative sample of biopsy reports from the available 543 reports. The expected sample size was 226, after adjusting using a correction formula, but we later decided to include all 469 eligible reports (see Figure 1, page 6) in the study based on the advice of our colleagues from the Epidemiology side that most of the published papers on medical literature tend to include all eligible cases as the study unit if it is feasible as outlined in our manuscript's references. We have cited these papers as examples from the references list in our manuscript (**see reference numbers 22 and 26**) because they have been published in well-known journals. This approach gave us a margin error of 1.67%, which we deemed acceptable. We were aware that having a sample that is too big or too small can waste resources or lead to unreliable results. We also needed to include small but significant factual information like the two cases of cervical screening, which made the sample size of 469 feasible for resource management. However, reviewer number 4 suggested increasing the study units, which we agree with, but there were no well-documented biopsy reports other than 2017/18 and 2018/19 at the time of the study.
- The methodology section of our report is now expanded to include the logistic regression model and the formula we used. To run the regression, we utilized SPSS software. However, we did not include detailed statistical and analytical steps in the manuscript, as we presumed that readers understand how the output results are obtained. Additionally, we received feedback from reviewer 8 who suggested that we contextualize the model instead of providing a technical explanation. **See page 7**
- In the conclusion part, the phrase ‘‘those coming from peripheral areas’’ is replaced with ‘‘rural dwellers’’ for clarity. **See page 20**

7. Thank you very much Reviewer 7 for acknowledging the improvement and recommending the manuscript for publication, I am eternally grateful.
8. Thank you, Reviewer 8, for evaluating the manuscript and for your insightful comments, and the responses and explanations are provided below.

- The problem is stated in the rationale of the study and the significance of the study subheadings of the introduction. **See pages 3 and 4**
- The methodology section of our report is now expanded to include the regression model we used and the reference for it. **See page 7**
